# Widespread recessive effects on common diseases in a cohort of 44,000 British Pakistanis and Bangladeshis with high autozygosity

**DOI:** 10.1016/j.ajhg.2025.03.020

**Published:** 2025-04-29

**Authors:** Teng Hiang Heng, Klaudia Walter, Qin Qin Huang, Juha Karjalainen, Mark J. Daly, Henrike O. Heyne, FinnGen, Daniel S. Malawsky, Georgios Kalantzis, Genes & Health Research Team, Sarah Finer, David A. van Heel, Hilary C. Martin

**Affiliations:** 1 Wellcome Sanger Institute, Wellcome Genome Campus, Hinxton CB10 1SA, UK; 2 Broad Institute, 415 Main Street, Cambridge, MA 02142, USA; 3 Hasso Plattner Institute, 14482 Potsdam, Germany; 4 Wolfson Institute for Population Health, Queen Mary University of London, London E1 4NS, UK; 5 Blizard Institute, Queen Mary University of London, London E1 2AT, UK

**Keywords:** recessive effects, GWAS, common diseases, complex traits, autozygosity, non-European ancestry, imputation

## Abstract

Genetic association studies have focused on testing additive models in cohorts with European ancestry. Little is known about recessive effects on common diseases, specifically for non-European ancestry. Genes & Health is a cohort of British Pakistani and Bangladeshi individuals with elevated rates of consanguinity and endogamy, making it suitable to study recessive effects. We imputed variants into a genotyped dataset (*n* = 44,190) by using two reference panels: a set of 4,982 whole-exome sequences from within the cohort and the Trans-Omics for Precision Medicine (TOPMed-r2) panel. We performed association testing with 898 diseases from electronic health records. 185 independent loci reached genome-wide significance (*p* < 5 × 10^−8^) under the recessive model, with *p* values lower than under the additive model, and >40% of these were novel. 140 loci demonstrated nominally significant (*p* < 0.05) dominance deviation *p* values, confirming a recessive association pattern. Sixteen loci in three clusters were significant at a Bonferroni threshold, accounting for multiple phenotypes tested (*p* < 5.4 × 10^−12^). In FinnGen, we replicated 44% of the expected number of Bonferroni-significant loci we were powered to replicate, at least one from each cluster, including an intronic variant in patatin-like phospholipase domain-containing protein 3 (*PNPLA3*; rs66812091) and non-alcoholic fatty liver disease, a previously reported additive association. We present evidence suggesting that the association is recessive instead (odds ratio [OR] = 1.3, recessive *p* = 2 × 10^−12^, additive *p* = 2 × 10^−11^, dominance deviation *p* = 3 × 10^−2^, and FinnGen recessive OR = 1.3 and *p* = 6 × 10^−12^). We identified a novel protective recessive association between a missense variant in *SGLT4* (rs61746559), a sodium-glucose transporter with a possible role in the renin-angiotensin-aldosterone system, and hypertension (OR = 0.2, *p* = 3 × 10^−8^, dominance deviation *p* = 7 × 10^−6^). These results motivate interrogating recessive effects on common diseases more widely.

## Graphical abstract

**Figure:**
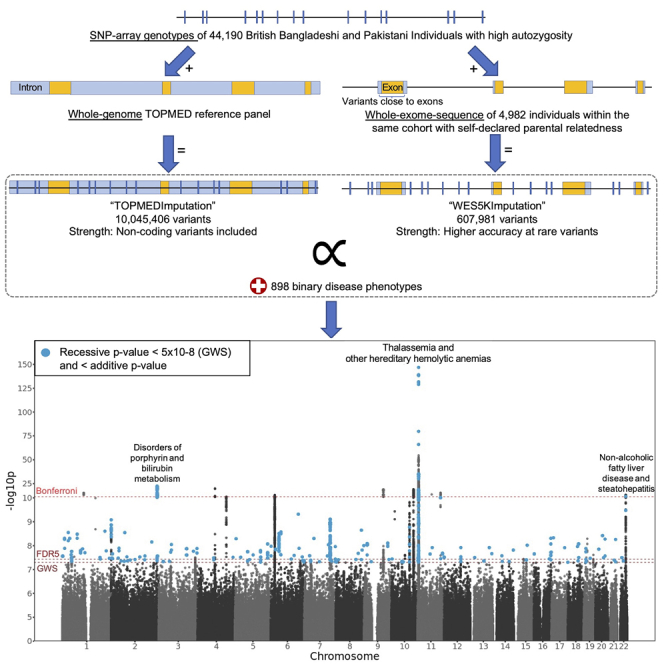

## Teaser

We leveraged the increased homozygosity in the Genes & Health cohort to perform recessive association testing between 898 diseases and 10,045,406 whole-genome imputed variants, as well as 607,981 whole-exome imputed variants. We identified 185 independent loci and described several examples. This motivates interrogating recessive effects on common diseases more widely.

## Introduction

Recessive effects in humans have been primarily studied in the context of rare, monogenic disorders, and little is known about recessiveness in common diseases and complex traits.^1^ Identifying variants with recessive associations with diseases could improve polygenic risk scoring, provide better insight into gene and variant function, improve understanding of disease pathophysiology, and allow for the identification of novel drug targets.^2^^,^^3^^,^^4^

The effects of genetic variation on common complex phenotypes are typically discovered through genome-wide association studies (GWASs),^5^^,^^6^^,^^7^ where an additive model is predominantly tested. However, applying a recessive model has allowed for the discovery of associations that would have been otherwise missed under conventional additive testing. For example, Heyne et al.^8^ performed recessive tests on 44,370 variants and 2,444 diseases in the FinnGen project from Finland and identified 31 loci at genome-wide significance (GWS; *p* < 5 × 10^−8^) where the associations were more significant in the recessive model than in the additive model. Notably, of the 20 findings further validated, 13 loci would have been missed with the additive model alone. Similarly, Guindo-Martinez et al.^9^ performed non-additive association testing in 62,281 subjects across 22 age-related diseases, and among 26 novel loci, four were identified only with the recessive model. Palmer et al.^1^ systematically quantified the contribution of dominance deviations (deviation from the additive pattern of inheritance) to heritability across 1,060 common traits in the UK Biobank (UKBB; *n* = 361,194). They identified non-additive effects of 183 phenotype-locus pairs across the phenotypic spectrum but concluded that, overall, non-additive effects contribute very little to heritability. Collectively, these results suggest that many recessive associations on common traits still remain to be found through the application of non-additive testing, including with rare variants, where the additive model would be more likely to miss recessive effects.

With the construction of large-scale biobanks, it is now possible to study the recessive contribution to common diseases.^8^^,^^10^ However, in outbred populations, very large sample sizes are required for adequate power to test recessive effects rather than additive effects, particularly for rare variants. The power to detect recessive effects is expected to be increased in bottlenecked populations such as Finland, where recessive variants may rise in frequency due to founder effects,^8^^,^^11^ or in populations enriched for consanguinity and, therefore, increased homozygosity. We showed in simulations that the power to find recessive effects is boosted both by explicit testing of a recessive model and by increased homozygosity (Note S1).

Genes & Health (G&H) is a community-based cohort of, at present, ∼60,000 individuals of British Bangladeshi and Pakistani ancestry with genetic data and linked electronic health records (EHRs).^12^ The cohort has a high rate of consanguinity (32% are offspring of second cousins or closer).^13^ Furthermore, British Pakistanis, who comprise 40% of the cohort, have been previously found to have high levels of endogamy as a result of the biraderi system, and multiple bottlenecked subpopulations display elevated levels of identity-by-descent (IBD) sharing, more than 10–20 times the level found in the Finnish population.^13^^,^^14^ We therefore hypothesized that the increased IBD sharing within these subgroups in G&H might allow reasonable quality imputation of variants even from a relatively small sample size of individuals from the same cohort. From this imputation, we would be able to take advantage of the increased homozygosity to test for recessive effects.

We leveraged genotype chip data on 44,000 G&H individuals, of whom around 5,000 also had whole-exome sequencing (WES) data. First, we imputed variants from the exome-sequenced individuals into the larger genotyped G&H cohort. This was inspired by Barton et al.,^15^ who boosted the power for association testing of rare coding variants by building a within-cohort reference panel from 49,960 WES samples in the UK Biobank and imputing variants into the larger genotyped cohort (*n* = ∼500,000). We also used an additional imputation reference panel, the Trans-Omics for Precision Medicine (TOPMED-r2) panel (97,256 individuals, including 644 with South Asian ancestry)^16^ to perform whole-genome imputation to study recessive effects in the noncoding regions. Using these two imputed datasets, we performed association testing with binary phenotypes curated from the EHRs, focusing on detecting recessive effects. We then characterized the recessive associations that we identified, systematically testing for replication and searching for literature support for each finding. In order to be objective when deciding which of our findings are “novel,” we have applied a standardized set of criteria, which are summarized in Note S10.

## Methods

### Summary of data collection

Participants were recruited into the G&H cohort with individual written informed consent, and data analysis is compliant with the General Data Protection Regulation (GDPR). The study has ethical approval (14/LO/1240) from the London South East NRES Committee of the Health Research Authority. More details are described in the supplemental acknowledgments. G&H data are available for analysis in a secure trusted research environment. Application can be made to the G&H executive: https://www.genesandhealth.org/researchers/apply-for-access/. Information on how to access FinnGen data can be found here: https://www.finngen.fi/en/access_results.

### Preparation of genetic data

#### Preparation of the genotyped data

Detailed quality control (QC) of the genetic data is described in Note S2. Genome-wide genotyping was performed with the Illumina Global Screening Array (GSAv3EAMD, build 38), and these data were used as the imputation backbone. Initial QC has been described in Huang et al.^17^ From the genotyped 44,396 individuals, we inferred 44,190 individuals to be of either Bangladeshi or Pakistani genetic ancestry (Note S3), and downstream analyses were restricted to these individuals. Another round of variant filters was applied to include only autosomal, bi-allelic SNPs with a ≥99% call rate. The Pakistani subgroup has high autozygosity and strong population structure, whereas the Bangladeshi subgroup has minimal structure and much less autozygosity,^18^ so to avoid excluding too many high-quality variants as a result of failure on a standard test for Hardy-Weinberg equilibrium (HWE), we performed the HWE test (with PLINK 1.9)^19^ only in the Bangladeshi subgroup, and the variants that failed a *p* value threshold of 10^−6^ in Bangladeshis were then excluded from the entire dataset. Variants with a minor-allele frequency (MAF) > 0.1% were included from the imputation backbone, which resulted in 469,678 variants that were then phased with EAGLE2 (Algorithm option Kpbwt = 20,000).^20^

#### Preparation of the within-cohort imputation panel

Exome sequencing was performed with Agilent v.5 capture kits on a subset of 5,236 individuals who self-declared as having consanguineous parents. Mapping, calling, and initial QC included excluding samples with sex discrepancies and <10× on-target coverage and applying the following variant filters by using bcftools^21^: “QD < 2.0 || FS > 30 || MQ < 40.0 || MQRankSum < −12.5 || ReadPosRankSum < −8.0” for SNPs and “QD < 2.0 || FS > 30 || ReadPosRankSum < −20.0” for insertions or deletions (indels; referenced from the G&H September 2019 summary files, please see web resources). We restricted the analysis to the 5,073 individuals who were genetically inferred to be of Bangladeshi or Pakistani ancestry from their array data. Then, we set genotypes that had a genotype quality (GQ) < 20, a *p* value from a binomial test for allele depth at heterozygous sites (binomAD) < 10^−2^, or a depth ≤ 7 to missing. Variants were excluded if they had a post-genotype QC call rate < 70%. 91 samples with high missingness or high discordance with their array data were excluded, leaving 4,982 samples (Note S4). The WES data were then merged with the SNP-array data and phased with EAGLE2 (Kpbwt = 20,000) to form a reference panel for imputation.

#### Imputation

We imputed the G&H data against two different imputation panels. Firstly, variants were imputed from the within-cohort whole-exome reference panel (described above) into the individuals without WES data with Minimac4.^22^ To assess the imputation accuracy and determine an imputed r^2^ cutoff, ten “leave-10%-out” trials were performed (Note S4). We retained variants with an imputed r^2^ ≥ 0.5 and at least three individuals with a homozygous genotype. This is referred to as the “WES5Kimputation” dataset. The SNP-array data were also submitted to the TOPMed-r2 Minimac4 1.5.7 Imputation Server^16^^,^^22^^,^^23^ for whole-genome imputation against the TOPMED-r2 panel. The same post-imputation filters were applied, and this is referred to as the “TOPMEDimputation.”

#### Variant annotation

Variants were annotated with Ensembl’s Variant Effect Predictor (VEP) v.107. For each variant, the worst consequence for any transcript was extracted for subsequent analyses.

### Phenotype curation

Two lists of phenotypes were curated from participants’ EHRs, and phenotype information was encoded as a binary, with “1” coding for a case and “0” coding for a control. A list of 237 custom phenotypes was compiled manually, and a second set of 1,281 phenotypes were defined based on International Classification of Disease (ICD10) codes. Further detail is described in Malawsky et al. (methods section phenotypic data harmonization and preparation for G&H).^18^ We retained phenotypes with ≥30 cases and also classified them into those that affected both sexes or were sex-specific (i.e., occurred only in females or males). For sex-specific phenotypes, the cohort was filtered to the relevant sex for testing. This resulted in 898 phenotypes.

### Association testing

We performed association testing by using the two-step pipeline of REGENIE.^24^ In step 1, we fit the model by using variants from the SNP array; we used the leave-one-out cross-validation (LOOCV) scheme and a genotype block size of 1,000. Step 2 was performed under the additive model and the recessive model at a genotype block size of 1,000 and a *p* value threshold of 0.05, below which the approximate Firth correction was applied. The covariates included age, sex, age^2^, age × sex, age^2^ × sex, and the first ten principal components (PCs) from the principal-component analysis (PCA) on unrelated G&H individuals (Note S3).

We defined significant recessive associations as tests with *p* < 5 × 10^−8^ (GWS) and with a *p* value lower under the recessive model than under the additive model. We excluded the human leukocyte antigen (HLA) region as a result of complex linkage disequilibrium (LD; chr6: 25–35 Mb) and then defined independent loci with the following steps: (1) for each phenotype with significant tests, we identified the test with the most significant recessive *p* value as the lead variant. (2) We calculated the LD r^2^ by using PLINK 1.9 between the lead variant and variants within a ±1.5 Mb window of it. Variants with an LD r^2^ ≥ 0.25 with the lead variant were defined as being part of the same locus, according to previous work from FinnGen.^8^^,^^11^ (3) We then identified the next most significant variant among the remaining variants that are not part of the locus defined in the previous steps and repeated the loop.

We calculated dominance deviation *p* values to assess the evidence that our significant associations detected under the recessive model were really recessive. Specifically, for the lead variants of the significant recessive associations, we ran logistic regression in R, controlling for the same covariates, performing genotypic tests with 2 degrees of freedom as well as additive and recessive tests for comparison. A description of how this was performed and detailed comparisons of the results with those from REGENIE are described in Note S8. We also noted that pairs of individuals carrying the same homozygous genotype at these significant variants were significantly more likely to be first-degree relatives than expected by chance (Note S3). However, repeating the association testing using only unrelated individuals found similar results (Figure S5), suggesting REGENIE is adequately controlling for this relatedness.

### Testing for replication in FinnGen and GERA

FinnGen is a public-private collaboration to profile the genomic and digital healthcare data of ∼500,000 Finnish individuals, with the goal of uncovering novel biological and therapeutic insights into human diseases. As a recessive testing pipeline has been built in the cohort before,^8^ we used it as an independent cohort to assess replication. More details of the cohort are described in Note S12. Phenotypes were matched manually to FinnGen binary phenotypes curated from Finnish health registers (Table S7). The variants from FinnGen release 10 were then tested with the phenotypes using REGENIE under the recessive model, controlling for the covariates sex, age, 10 PCs, and genotyping batch, as described for release 10 on https://www.finngen.fi/.^11^ For each significant locus in G&H, we first identified proxy variants as variants that are within the window described above (±1.5 Mb from the lead variant with an LD r^2^ ≥ 0.25. For a more stringent assessment of replicability, we applied the locus definitions from Huang et al.^17^ and repeated the analysis (see Note S9). We defined a locus as replicating (or “transferable”) in FinnGen if any of the lead or proxy variants had the same direction of effect as observed in G&H at a nominally significant *p* < 0.05 when tested in a recessive model with a similar trait. To compare the number of loci that replicated to what we might have expected to replicate given the power in FinnGen, we calculated a power-adjusted transferability (PAT) ratio.^17^ Specifically, for each locus, we used the effect size of the lead variant in G&H, the allele frequency (AF) of the lead variant in FinnGen, and the case rate and sample size in FinnGen to estimate the power for the test with the genpwr R package.^25^ The expected number of transferable loci was calculated as the sum of the power estimates across all the loci. The PAT ratio was then calculated by dividing the observed number of transferred loci by the expected number.

Publicly available summary statistics from recessive testing in the Genetic Epidemiology Research on Aging (GERA) cohort by Guindo-Martinez et al.^9^ were accessed on December 14, 2023. Phenotypes were matched manually (Table S8), and the PAT ratio was calculated as described above, through the use of AFs and case rates in GERA.

## Results

### Recessive association testing identifies 185 loci

**Figure 1:**
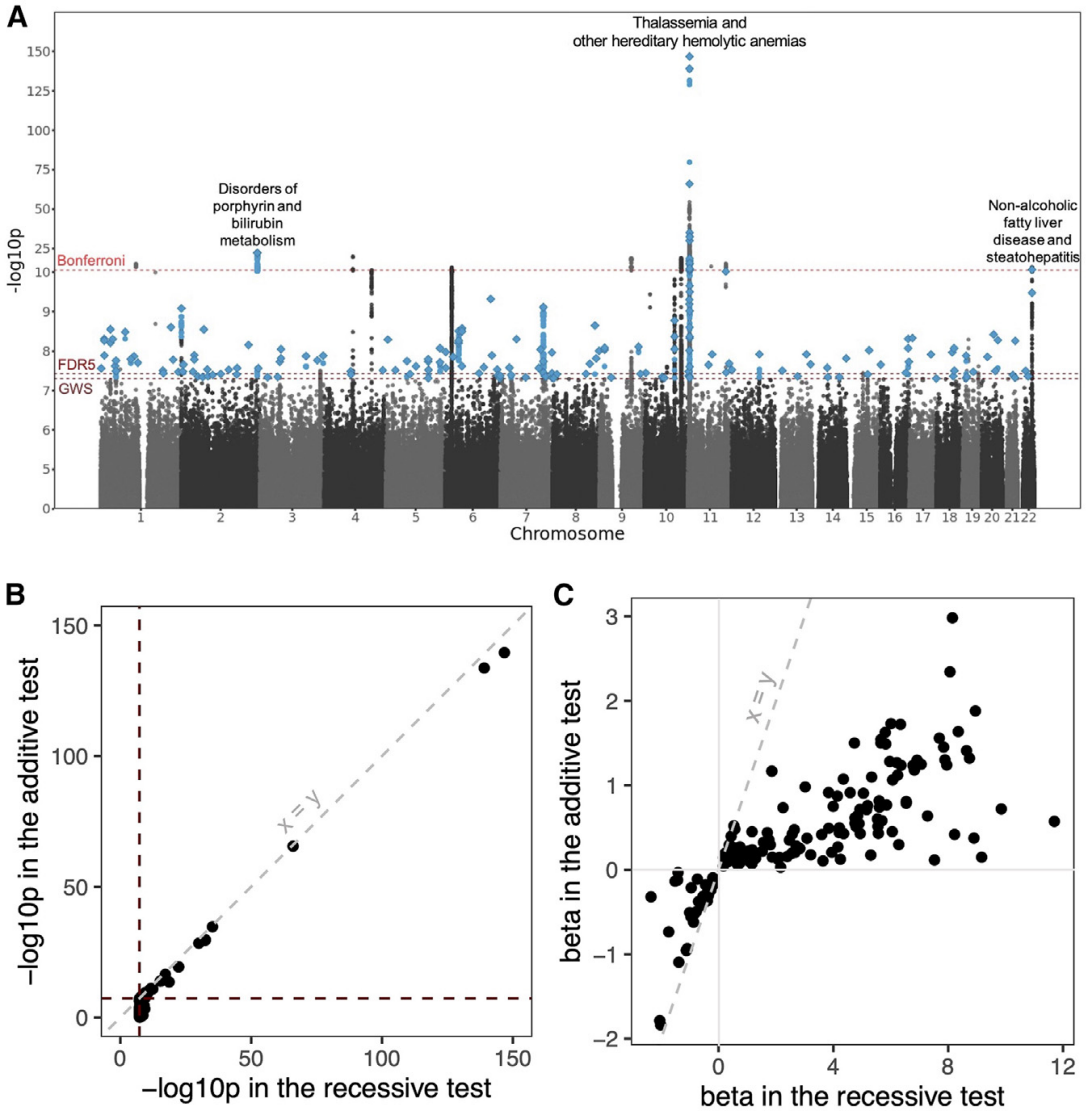
The 185 loci identified at genome-wide significance in recessive association tests (A) Manhattan plot of the results from recessive tests performed. Dashed horizontal lines represent the various *p* value cutoffs: genome-wide significance (GWS; *p* < 5 × 10^−8^), for a false discovery rate of 5% (FDR5; *p* < 3.7 × 10^−8^), and the Bonferroni-corrected cutoff (Bonferroni; *p* < 5.5 × 10^−12^). Blue points represent recessive tests passing the genome-wide significance threshold where the recessive *p* value is more significant than the additive *p* value, and diamonds represent lead variants defined as described in the methods. The clusters containing Bonferroni-significant associations are labeled with the phenotypes with which they are associated. (B) For the lead variants, the −log10 *p* from additive testing against −log10 *p* from recessive testing. The horizontal line indicates the genome-wide significance threshold, and the diagonal line is *y* = *x*. (C) The effect size (beta) under the additive versus recessive model for the lead variants. The additive beta is the log of the change in odds for carrying one alternate allele, while the recessive beta is the log of the change in odds for the homozygous carriers of the alternate allele compared to the rest of the cohort (reference homozygotes and heterozygotes combined).

Using exome-sequencing data from 4,982 individuals from G&H, we generated a reference panel, which allowed us to impute 605,263 variants, including rare exonic variants, into the larger cohort of 44,186 individuals from that cohort. Simultaneously, we carried out a whole-genome imputation of 10,045,406 variants using the TOPMED panel. We then tested these variants for recessive associations with 898 phenotypes. We identified 185 unique loci where the lead variant had a genome-wide-significant recessive *p* value (<5 × 10^−8^) that is smaller than the additive *p* value (Figure 1; Table S5). At a stringent Bonferroni cutoff (0.05/9,197,933,046 tests = 5.4 × 10^−12^; Note S5), 16 loci remained. 144 loci passed a more lenient Benjamini-Hochberg cutoff for a false discovery rate of 5% (FDR 5%) (*p* < 3.7 × 10^−8^, so close to the GWS threshold). Notably, the 16 Bonferroni-significant findings can be found in three clusters (Figure 1; Table 1), corresponding to (1) non-alcoholic fatty liver disease and steatohepatitis (NAFLD) (one locus; lead SNP chr22:43,939,790 [GRCh38]), (2) disorders of porphyrin and bilirubin metabolism (one locus, found with both imputation panels; lead SNP chr2:233,763,993), and (3) thalassemia and other hereditary hemolytic anemias (13 loci, one of which was found with both imputation panels; lead SNPs at chr11:4,908,482–5,544,800).

**Table 1:** Recessive findings that passed Bonferroni significance

**Gene**	**Lead variant**	**Consequence**	**Phenotype**	**Genes & Health**								**FinnGen**							
				**Imputation**	**AF**	**Recessive**		**Additive**		**Δ log10 *p***	**DD *p***	**Power**	**Recessive**				**Additive**		
						**OR**	***p***	**OR**	***p***				**OR**		***p***		**OR**	***p***	
*UGT1A6*	chr2:233763993G>T	intron	E80 (disorders of porphyrin and bilirubin metabolism)	WES5K	0.53	6.4	5 × 10^−23^	3.2	4 × 10^−20^	2.9	4 × 10^−3^	1	5.8		2× 10^−74^		3.0	4 × 10^−65^	
*UGT1A6*	chr2:233763993G>T	intron	E80 (disorders of porphyrin and bilirubin metabolism)	TOPMED	0.53	6.4	5 × 10^−23^	3.2	4 × 10^−20^	2.9	4 × 10^−3^	1	5.8		2× 10^−74^		3.0	4 × 10^−65^	
*UGT1A6*	chr11:5216054A>G	intergenic	D56 (thalassemia)	TOPMED	0.76	0.6	5 × 10^−16^	0.7	1 × 10^−14^	1.4	3 × 10^−3^	1	0.7^a^		4 × 10^−3^^a^		not reported		not reported
*OR52D1*	chr11:5489111C>T	synonymous	D56 (thalassemia)	WES5K	0.81	0.6	4 × 10^−13^	0.7	1 × 10^−14^	1.5	5 × 10^−3^	0.9	not replicated	not replicated			not reported		not reported
*HBE1*	chr11:5478732G>C	intron	D56 (thalassemia)	TOPMED	0.81	0.6	2 × 10^−16^	0.7	1 × 10^−14^	1.7	6 × 10^−4^	1	0.7^b^		8 × 10^−5^^b^		not reported		not reported
*HBE1*	chr11:5330439C>T	intron	D56 (thalassemia)	TOPMED	0.71	0.4	2 × 10^−12^	0.6	7 × 10^−12^	0.6	1 × 10^−2^	1	not replicated			not replicated	not reported		not reported
*HBE1*	chr11:5367606A>C	intron	D58 (other hereditary hemolytic anemias)	TOPMED	0.98	0.3	6 × 10^−36^	0.4	2 × 10^−35^	0.5	2 × 10^−3^	1	not replicated			not replicated	not reported		not reported
*HBG1*	chr11:5247392T>G	downstream gene	D58 (other hereditary hemolytic anemias)	TOPMED	0.61	0.4	2 × 10^−19^	0.6	3 × 10^−14^	5.1	9 × 10^−5^	1	not replicated			not replicated	not reported		not reported
*MMP26*	chr11:4931228C>A	intron	D58 (other hereditary hemolytic anemias)	TOPMED	0.79	0.4	6 × 10^−18^	0.6	3 × 10^−17^	0.7	4 × 10^−3^	1	0.8		3 × 10^−2^		not reported		not reported
*OR51A7*	chr11:4908482G>A	3′ UTR	D58 (other hereditary hemolytic anemias)	TOPMED	0.95	0.3	1 × 10^−66^	0.3	3 × 10^−66^	0.5	2 × 10^−8^	1	not replicated			not replicated	not reported		not reported
*OR51V1*	chr11:5197578AAT>A	downstream gene	D58 (other hereditary hemolytic anemias)	TOPMED	0.55	0.2	1 × 10^−30^	0.5	4 × 10^−29^	1.6	7 × 10^−7^	1	not replicated			not replicated	not reported		not reported
*OR52E1*	chr11:5067264A>G	upstream gene	D58 (other hereditary hemolytic anemias)	TOPMED	0.99	0.1	9 × 10^−140^	0.2	2 × 10^−134^	5.4	2 × 10^−14^	1	0.1^c^		1 × 10^−129^^c^		not reported		not reported
*OR52E2*	chr11:5058716C>T	synonymous	D58 (other hereditary hemolytic anemias)	WES5K	0.99	0.1	2 × 10^−147^	0.2	3 × 10^−140^	7.2	8 × 10^−19^	1	not replicated			not replicated	not reported		not reported
*OR52H1*	chr11:5544800A>G	missense	D58 (other hereditary hemolytic anemias)	WES5K	0.99	0.3	4 × 10^−33^	0.4	3 × 10^−30^	2.9	4 × 10^−4^	1	not replicated			not replicated	not reported		not reported
*OR52H1*	chr11:5544800A>G	missense	D58 (other hereditary hemolytic anemias)	TOPMED	0.99	0.3	3 × 10^−33^	0.4	2 × 10^−30^	2.7	4 × 10^−4^	1	not replicated			not replicated	not reported		not reported
*PNPLA3*	chr22:43939790TGG>T	intron	non-alcoholic fatty liver disease and steatohepatitis	TOPMED	0.61	1.3	2 × 10^−12^	1.2	2 × 10^−11^	0.9	3 × 10^−2^	0.9	1.4		6 × 10^−12^		not reported		not reported

The allele frequency (AF) and odds ratio (OR) presented are for the alternate allele (second allele in the lead variant ID). Footnotes are added to those that replicated by proxy variant. We checked whether additive results in FinnGen were reported in https://r10.finngen.fi/. We derived the power-adjusted transferability (PAT) ratio for the replication of Bonferroni-significant loci in FinnGene by summing the number of loci that replicated (7) and dividing it by the sum of the expected power for replication at each loci (sum of the “power” column: ∼16); this gave us a PAT of 44%. OR, odds ratio, converted from beta in the test output; Δ log10 *p*, the difference between the −log10 *p* in the recessive test and the −log10 *p* in the additive test; DD *p*, dominance deviation *p* value.

a Proxy variant that replicated is chr11:5212606C>G (LD r^2^ = 0.3).

b Proxy variant that replicated is chr11:5483160T>C (LD r^2^ = 0.4).

c Proxy variant that replicated is chr11:5011706A>G (LD r^2^ = 0.9).

Of the lead variants at the 185 recessive loci, 152 (82%) were not genome-wide significant in the additive test (Figure 1B), suggesting that they would have been missed in conventional additive GWASs. The associations that were not genome-wide significant in the additive test tended to be with rarer variants (Figure S15A). The effect sizes in the recessive tests for these lead variants also tend to have a larger magnitude than in the additive tests (Figure 1C). 76% (140) of these lead variants had nominally significant dominance deviation *p* values.

**Figure 2:**
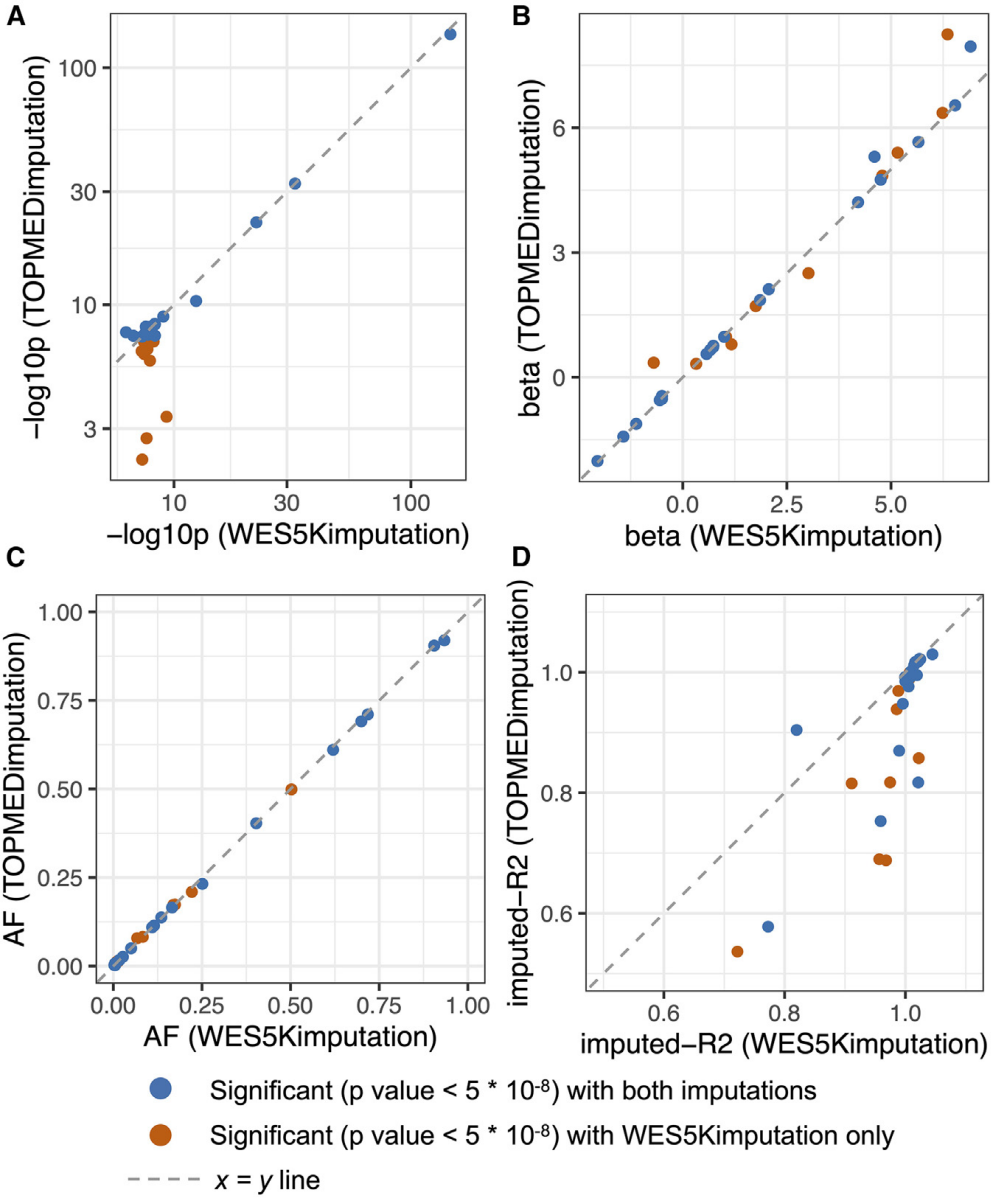
Comparison of results for variants tested using both imputation panels and that are significant in one or both Scatterplots show the (A) *p* values (−log10 *p*), (B) betas, (C) allele frequencies (AFs), and (D) imputed r^2^ values of lead variants.

These 185 recessive loci include loci that were significant in either or both of the imputation panels. Twenty-nine of the lead variants were successfully tested in both imputation sets. At these, the effect sizes (Figure 2B) and AFs (Figure 2C) correlated well between the two imputation panels, though interestingly, a small subset of findings significant with the WES5Kimputation had much less significant *p* values when their TOPMEDimputation genotypes were tested (Figure 2A). These findings corresponded to variants with lower imputed r^2^ values in the TOPMEDimputation compared to the WES5Kimputation (Figure 2D), suggesting that higher confidence in the imputation when using the in-house panel improved the sensitivity of the testing through more accurate prediction of the genotypes.

### Replication of significant findings in other cohorts

We sought to test how many of the 185 loci could be replicated in two other cohorts suitable for recessive association testing, FinnGen and GERA. 133 loci could be found in FinnGen, meaning the lead variant of the locus could be tested with a comparable phenotype in FinnGen. Of these 133 lead variants, 82 had the same direction of effect in the recessive tests of both cohorts. This is significantly more than the number expected by chance (one-sided exact binomial test, *p* = 0.0045).

Next, we calculated the PAT ratio,^17^ a quantitative measure of how many loci can be replicated in FinnGen relative to the power in this independent cohort; it takes into account AFs, case rates, and sample sizes. We expected to replicate 129 of the 133 examined loci at *p* < 0.05; of these, 30 loci replicated in FinnGen, resulting in a PAT ratio of 23%. This suggests that a *p* < 5 × 10^−8^ cutoff for significance in G&H is too lenient, given the number of phenotypes tested. Restricting to the Bonferroni-significant findings, the PAT ratio increased to 44%. The loci associated with NAFLD and with disorders of porphyrin and bilirubin both replicated. For the cluster of loci associated with thalassemia and other hereditary hemolytic anemia, four out of 13 loci replicated, while eight of the remaining nine loci had the same direction of effect in both cohorts (although we note that we were not considering identical phenotypes in FinnGen since they were not available; see Table S8). We also assessed replication with a variety of more stringent cutoffs, such as applying stricter locus definitions (which would inflate the number of independent findings), considering only lead variants for replication, and changing *p* value thresholds, as shown in Note S9. The broad conclusions remain the same: about ∼40% to ∼70% of loci replicate by their lead SNP alone, depending on the replication criteria (Table S11), and Bonferroni-significant loci had a higher percentage of replication than genome-wide-significant loci and were more robust to more stringent thresholds for replication.

We also assessed replication in the GERA cohort. The 22 phenotypes tested by GERA restricted the number of phenotypes we could match, so only 11 GWS loci could be evaluated. After accounting for the AF, case rate, and sample size differences, we expected to replicate around eight loci. Four loci replicated, resulting in a PAT ratio of 48%.

Finally, we looked up our significant recessive associations in the genome-wide scans performed in Palmer et al.^1^ that tested for deviation from the additive effect across traits in ∼361,000 unrelated individuals of European ancestry in the UK Biobank. Among our 185 significant associations, 71 were also tested in the UK Biobank (i.e., the leading variant was tested for dominance deviation from the additive effect on a phenotype related to the one tested in G&H). Nine of them showed nominally significant dominance testing results with a concordant direction of effect (Table S5). Of these nine loci, seven replicated in FinnGen, and three of four loci that replicated in GERA replicated here as well.

### The Bonferroni-significant recessive associations

The Bonferroni-significant lead variants are listed in Table 1. We summarize these significant associations below.

Firstly, we found a recessive association between rs66812091 (chr22:43939790TGG>T), an intronic variant in *PNPLA3* (patatin-like phospholipase domain-containing protein 3), and NAFLD, with an odds ratio (OR) of 1.3 at a *p* value of 2 × 10^−12^. This *p* value is an order of magnitude more significant in the recessive test than the additive test (2 × 10^−11^). The relationship also had a significant dominance deviation *p* value of 3 × 10^−2^ and was replicated in FinnGen with a recessive test OR of 1.3 and a *p* value of 6 × 10^−12^. *PNPLA3* is involved in lipid and fatty acid metabolism and has high expression in adipose and liver tissues.^26^^,^^27^ This gene’s role in fatty acid metabolism and liver function has been further supported by functional knockout studies in mice.^27^^,^^28^ This variant has also been shown in additive GWASs to be associated with NAFLD (in the FinnGen release 6 dataset) and deranged liver enzymes (a marker of hepatitis).^29^ However, to our knowledge, this is the first study demonstrating that, in fact, one or more of the variants near this gene are likely to have recessive rather than additive effects on NAFLD.

Secondly, an intronic SNP in *UGT1A6*, rs6742078 (chr2:233763993G>T), identified with both imputation panels, was found to be associated with disorders of porphyrin and bilirubin metabolism. *UGT1A6* encodes a uridine 5′-diphospho-glucuronosyltransferase (UDP-glucuronosyltransferase [UGT]). UGTs are enzymes responsible for the glucuronidation of lipophilic molecules, including bilirubin, into the conjugated, hydrophilic form that can be excreted in the urine.^30^ *UGT1A6* has been shown to be associated with bilirubin levels in the GWASs on liver traits and bilirubin levels.^31^^,^^32^ Mutations in UGTs, specifically *UGT1A1*, cause the well-known autosomal recessive conditions Gilbert’s syndrome^33^ and Crigler-Najjar syndrome,^33^^,^^34^ in which affected individuals present with excess unconjugated bilirubin. The findings presented here demonstrate novel recessive associations between another UGT not previously reported and dysfunctional bilirubin clearance.

Thirdly, we found multiple Bonferroni-significant recessive hits in the genomic region chr11:4,908,482–5,544,800 associated with thalassemia and other hereditary hemolytic anemias. Although many variants in this region have been known to associate with these phenotypes (Note S11), among the four hits that replicate in FinnGen, two (chr11:4931228C>A and chr11:5067264A>G) have no reported associations in the literature, additive or otherwise, and can be considered novel; the remaining two hits (chr11:5216054A>G and chr11:5478732G>C) have been associated with multiple anemia traits in additive GWASs,^35^^,^^36^ and we present support that their underlying association pattern may be recessive instead (Table 1; Note S10). The variants span two hemoglobin genes, hemoglobin epsilon locus (*HBE1*) and hemoglobin gamma a (*HGB1*), as well as the gene *MMP26* and a cluster of olfactory receptor genes. The two hemoglobin genes *HBE1* and *HGB1* encode embryonic and fetal hemoglobin (HbF) subunits, respectively. The gene hemoglobin subunit gamma 2 (*HBG1*) is well established to be associated with β-thalassemia and hemoglobin E disease.^37^ β-thalassemia is an inherited anemia caused by dysfunctional production of the β-globin subunit of adult hemoglobin (HbA)^38^; therefore, the persistence of elevated HbF past infancy as a compensatory mechanism is occasionally observed.^39^ Genes encoding embryonic hemoglobin and HbF, including *HBE1* and *HGB1*, have been targeted as part of possible therapies in thalassemias, where reactivating these alternative forms of hemoglobin expression may help to supplement low HbA production.^40^^,^^41^^,^^42^ With regard to the variants in olfactory receptor genes, there is some evidence in the literature reporting on the relationship between olfactory genes and thalassemia. The β-globin gene *HBB*, in which pathogenic variants cause β-thalassemia, is sandwiched between olfactory receptor gene clusters,^43^ and β-globin deletions that cause thalassemia have been known to extend to these olfactory genes.^44^^,^^45^ In addition, one of the olfactory genes in this region, *OR52A1*, contains an enhancer for γ-globin, and variants that disrupt the function of this enhancer may exacerbate anemia in individuals with thalassemia.^46^ As the variants we have reported are in close proximity to multiple hemoglobin genes, including *HBB*, we tested if they were in LD with variants in hemoglobin genes but found no such evidence within G&H. We think it is likely that these variants are expression quantitative trait loci (eQTLs) or splice QTLs (sQTLs) for hemoglobin genes. Indeed, we found one additional eQTL, chr11:5489111C>T, the synonymous variant in *OR52D1*, that is an eQTL associated with changes in the expression of *HBG2* in blood, as reported in eQTLGen.^47^ However, we cannot robustly test this in the absence of large-scale single-cell eQTL and sQTL datasets because the QTLs might be specific to certain cell types or states.

### Recessive findings implicating coding variants provide insight into gene functions

We observed a significant enrichment of recessive associations among coding variants (Note S7). Out of the 10,045,406 variants of the TOPMEDimputation that were tested, 0.044% of coding variants had significant recessive associations compared to 0.011% in noncoding variants (chi-squared test *p* = 8.3 × 10^−20^). This is consistent with the expectation that protein-coding variants are more likely to impact gene function and health outcomes and that they have larger effect sizes, leading to better power for detection. Since these coding variants seem likely to be the causal variants at those loci, we discuss several examples here.

Firstly, we see a recessive association between a missense variant (chr1:11796321G>A, rs1801133; c.677C>T [p.Ala222Val]) in the gene encoding methylenetetrahydrofolate reductase (MTHFR) and folate deficiency (OR = 2.1, *p* = 5 × 10^−9^, dominance deviation *p* = 10^−3^). This finding was also replicated in FinnGen (OR = 2.1, *p* = 0.02). MTHFR is an enzyme involved in folate and homocysteine metabolism. After folate is converted to 5,10-methylenetetrahydrofolate (5,10-MTHF), MTHFR reduces 5,10-MTHF to 5-methyltetrahydrofolate (5-MTHF), which is then required as a cosubstrate for the conversion of homocysteine to methionine.^48^ The missense variant we report (c.677C>T) has been shown to cause instability in MTHFR, resulting in the accumulation of homocysteine.^49^ Indeed, there are reports that this c.677C>T mutation has a recessive effect on homocysteine levels but with a mild heterozygous effect: individuals heterozygous for the c.677C>T mutation have mildly elevated homocysteine levels, while the homozygous individuals have significantly higher levels.^50^^,^^51^^,^^52^ Functional studies have shown that the enzyme’s reduced efficiency from instability caused by the c.677C>T transition can be compensated with additional folate.^51^^,^^53^^,^^54^^,^^55^ This implies that the reduced enzyme efficiency from the c.677C>T transition results in low folate levels, consistent with our association. Multiple additive GWASs have reported associations between this variant and folate deficiency anemia (in the FinnGen release 6 dataset) or being on folate supplements (in the UK Biobank GWAS round 2 results). However, our study suggests that the underlying pattern of inheritance may be recessive instead. Folate is essential for DNA, RNA, and protein methylation, and fetal neural tube defect is an established consequence of low folate levels in pregnancy.^56^ Germane to this, there are several reports (although conflicting) of recessive associations between variants (including c.677C>T) in *MTHFR* and neural tube defects.^57^^,^^58^^,^^59^^,^^60^

Secondly, we have found a protective recessive association between a missense variant in solute carrier (SLC) family 5 member 9 (*SLC5A9*) (chr1:48228922G>A, rs61746559) hypertension (OR = 0.2, *p* = 3 × 10^−8^, dominance deviation *p* = 7 × 10^−6^). SLC5A9 is also known as sodium-glucose transporter 4 (SGLT4) and is a member of the SLC superfamily. Specifically, it is a sodium-dependent glucose transporter of mannose, 1,5-anhydro-D-glucitol, and fructose.^61^ Another member of this family is the well-studied SGLT2, a glucose transporter largely expressed in the kidney and which is a target of gliofozins, drugs used for lowering serum glucose levels in individuals with type 2 diabetes.^62^ It has been shown that a concomitant benefit of inhibiting SGLT2 in type 2 diabetes is lowering blood pressure,^63^^,^^64^^,^^65^ possibly from hemodynamic changes in kidney glomeruli^66^ and regulating the renin-angiotensin-aldosterone system.^67^ SGLT4 may function in a similar manner, as, like SGTL2, it is expressed in the kidneys.^61^ Missense mutations might inhibit its function, explaining the relationship we detect with hypertension. Although there is limited information about this gene’s function currently^68^ to support this hypothesis, there is an additive association between this variant and renin levels,^69^ suggesting it might indeed be involved in the renin-angiotensin-aldosterone system. Multiple widely used classes of antihypertensives work on the renin-angiotensin-aldosterone system, which plays a key role in regulating blood pressure.^70^

### Quantifying novelty in the recessive findings

For the 185 recessive loci reported, we next systematically searched for associations between the lead variant and the same or related trait in the literature. In an effort to quantify the novelty in our findings, we developed a “literature evidence score” to characterize the strength of the literature support for each finding (the higher the score, the stronger the evidence). The details of the approach are described in Note S10. In summary, ∼40%–80% of our reported associations are novel, depending on whether a strict (no reported association to a related trait) or lenient (no reported association to the same trait) definition of novelty is used. We found that the strength of the support in the literature was associated with a smaller difference in the recessive and additive *p* values for the test (linear regression of delta log10 *p* against our literature evidence score; slope: −0.32, *p* = 5.4 × 10^−5^; Figure S18)—in other words, associations that seem more likely to be truly recessive are less likely to have support already in the literature. This reiterates the importance of performing recessive testing on top of just additive testing and in cohorts with better power for the recessive tests, as these findings would potentially have been missed from additive testing alone.

## Discussion

After performing recessive association testing on variants imputed from both a within-cohort exonic reference panel and the whole-genome TOPMED reference panel, 185 unique loci were identified at GWS, where the recessive association was more significant than the additive association. After Bonferroni multiple testing correction, 16 loci in three clusters remained. After adjusting for changes in case rates, AFs, and sample size in the independent cohort FinnGen, we replicated 44% of the expected number of Bonferroni-significant loci we would be powered to replicate, including at least one locus from each cluster. We also identified recessive associations at loci previously thought to be additive. Examples include the association between rs66812091 and NAFLD and that between rs1801133 and folate deficiency. Notably, we report a novel recessive association between a missense variant in *SGLT4* (rs61746559) and a reduced risk of hypertension.

In modeling binary traits, one usually assumes a liability threshold model, in which an individual develops the disease once they pass a certain threshold on a continuous, quantitative trait (the liability) that follows a normal distribution in the population.^71^ Under this model, it is possible that a variant may have an additive effect on the underlying liability but a recessive effect on disease status. For example, it may be that rs66812091 in *PNPLA3* has an additive effect on liver enzyme levels, leading to the accumulation of fatty acids that results in hepatic inflammation (i.e., the underlying quantitative trait), but a recessive effect on NAFLD (*p* = 2.4 × 10^−12^). Supporting this, Barton et al.^72^ showed in the UK Biobank that variants with known recessive associations to disease can have milder heterozygous effects in related quantitative traits. For our recessive findings, we searched for additive associations to relevant quantitative traits in Jacobs et al.,^73^ where additive GWASs on 42 blood-based quantitative traits had been performed in G&H. We found that out of the 54 loci that we expected to show a quantitative trait association, 19 loci, or 35% of the findings, indeed had relevant significant additive quantitative-trait associations (Note S10). Therefore, characterizing the underlying inheritance pattern in greater detail could improve our understanding of disease pathophysiology and highlight homozygous individuals as a higher-risk group to target during screening.

By extension, the patterns of association between a variant and a disease may be context specific and may differ across ancestries. These complex traits are multifactorial, and differing disease pathophysiologies and environmental effects result in different effect sizes^74^ and may affect inheritance patterns as well. This might contribute to the differences in heritability across ancestries^75^ and the difficulty in transferring polygenic risk scores to other ancestries.^17^ This further emphasizes the importance of performing genetic analyses in diverse cohorts, such as G&H.^76^

With G&H, we were able to perform imputation with a within-cohort reference panel and demonstrated the advantage of using this to improve sensitivity, as previously reported by Barton et al.^15^ We saw a subset of recessive findings with *p* values that were only significant with the WES5Kimputation and not the TOPMEDimputation. We hypothesize from our evaluation of imputation accuracy (Note S4) that this might be due to more accurate imputation of these variants with the within-population reference panel. However, the TOPMEDimputation provided a significantly larger set of variants to work with, which probably explains why most of our associations are from that panel.

Our recessive testing highlights the value of detecting novel recessive associations that would have been missed under the additive model. For example, the rs61746559 missense variant affecting *SGLT4* was previously reported to be additively associated with renin levels, and together with our novel finding that the homozygous genotype is protective for hypertension, it may provide a basis for considering the gene as a drug target. The majority of the GWS findings (152/185) were not GWS under the additive model, illustrating the importance of applying the recessive model of testing to find recessive effects. For example, we showed that an intronic variant (chr13:109179501G>C, rs2038707) in myosin 16 (*MYO16*), a gene involved in the musculoskeletal system,^77^^,^^78^ had a recessive association with the ICD10 code M85, “other disorders of bone density and structure” (OR = 3.2, *p* = 2 × 10^−8^, dominance deviation *p* = 10^−5^). It would likely have been missed under the additive model (OR = 1.3, *p* = 9 × 10^−3^). This example also illustrates the value of applying recessive testing to a cohort enriched for homozygosity. The AF for this variant in G&H is 0.17, which is similar to the AF in non-Finnish Europeans (NFE) of 0.16.^79^ The NFE population has low levels of consanguinity; therefore, at a sample size of 44,000, one would expect, on the basis of the HWE, that the number of homozygous individuals would be ∼1,126 and thus that the power to perform this recessive test in this population would be 57%. As a result of increased autozygosity, the observed number of homozygotes in G&H is 1,408, giving 82% power. Therefore, it is possible that additive tests in larger non-consanguineous cohorts may have missed this recessive association. An exome-based study of 394,841 UK 3Biobank individuals and 4,529 phenotypes detected a nominally significant gene-based additive association between putative loss-of-function variants in *MYO16* with the same ICD10 code (OR = 1.1, *p* = 5 × 10^−4^).^80^ Furthermore, additive tests with FinnGen (release 6) report nominally significant associations between our lead variant and related phenotypes such as fibroblastic disorders (OR = 1.1, *p* = 10^−3^) and benign neoplasms in the scapula and long bones of the upper limb (OR = 0.7, *p* = 10^−3^; FinnGen release 6 dataset).

Another study within Genes & Health, Malawsky et al.,^18^ demonstrated that increased homozygosity (higher F_ROH_) was associated with multiple common diseases. The primary hypothesis for these associations is that homozygous regions of the genome contained causal variants with recessive effects on these phenotypes.^81^ From the meta-analysis of highly consanguineous subsets of Genes & Health and UK Biobank in that study, F_ROH_ was found to be associated with 12 ICD10 subchapters (passing the FDR 5% multiple testing threshold). Five of the 12 (42%) phenotypes significantly associated with F_ROH_ had underlying single-variant recessive associations reported in this study, compared to 13 of the remaining 49 (27%) phenotypes tested in that paper, which were not significantly associated with F_ROH_ (Fisher’s exact test *p* = 0.31). Manually relaxing the phenotype matching for the 12 phenotypes associated with F_ROH_, we found variants with significant (*p* < 5 × 10^−8^) recessive associations to three more closely related phenotypes (Table S10), although none passed our more stringent Bonferroni correction threshold. This supports the hypothesis that the association between increased autozygosity and the prevalence of some common diseases is due to underlying genetic variants with recessive effects.

There are several limitations to the project. Firstly, we have not carried out fine-mapping of these recessive associations because, to our knowledge, there are no established methods for fine-mapping that would help disentangle, for example, a recessive hit that is in LD with a strongly additive hit. However, it is worth noting also that LD diminishes with distance between non-additive variants at a rate that is squared of the rate between additive variants,^1^ which should, in theory, make it easier to pinpoint causal recessive variants due to the lower LD. Secondly, there is limited replication of findings with *p* values slightly below the GWS threshold. This might be because this *p* value cutoff is not stringent enough, given the number of tests we performed. Thirdly, our power calculations have various limitations. They were performed on a simple logistic regression model, so may have overestimated the power of the model fitted by SAIGE and REGENIE. Furthermore, effect sizes used in the power calculation might be overestimated in the discovery GWAS (winner’s curse), which could also lead to overestimated power. Additionally, differences in the granularity of disease classification and differing methods for phenotype curation are not accounted for in the power calculation. For example, there is no thalassemia phenotype available for testing in FinnGen (or GERA), and the G&H phenotypes of “thalassemia” and “other hereditary hemolytic anemia” were matched to “other anemia” and “hemolytic anemia” in FinnGen, which may have contributed to the poorer replication in that cluster of loci compared to the other Bonferroni-significant loci. Lastly, the Finnish cohort and the GERA cohort are composed of very different ancestries from British South Asians, and the differences in LD patterns between populations could be significant. In particular, the relatively poor replication of Bonferroni-significant variants associated with thalassemia and hereditary anemias in FinnGen might be because selection for resistance to malaria in South Asia has produced complex LD patterns in that genomic region^82^^,^^83^ that differ from the LD patterns in European-ancestry samples; these are not accounted for in our power calculation. Finally, another limitation is that we only carried out single-variant tests. In the future, exome sequencing of the full G&H cohort will allow us to identify rare variants that can be aggregated within a gene to try to boost the power to find genes with recessive effects and reduce the need for fine-mapping.

In conclusion, with our whole-exome and whole-genome imputation sets, we profiled the recessive landscape at single variants in this cohort of 44,000 British South Asians across a broad spectrum of clinical phenotypes and identified 185 recessive associations. It is likely that many recessive findings remain to be found, and this project provides a sound argument for expanding the search to other cohorts and phenotypes.

## Consortia

The consortium members that make up the G&H Research Team are Ahsan Khan, Amna Asif, Ana Angel, Annum Salman, Asma Qureshi, Benjamin M Jacobs, Bill Newman, Caroline Morton, Caroline Winckley, Ceri Durham, Chris Griffiths, Claudia Langenberg, Dan Mason, Daniel MacArthur, Daniel Stow, David A Van Heel, David Collier, Eamonn Maher, Elizabeth Owor, Emily Mantle, Fabiola Eto, Georgios Kalantzis, Gerome Breen, Grainne Colligan, Hanifa Khatun, Hilary Martin, Iaroslav Popov, Ishevanhu Zengeya, Jessry Russell, Joanne Harvey, John Solly, John Wright, Joseph Gafton, Julia Zöllner, Kamrul Islam, Karen A Hunt, Karen Tricker, Klaudia Walter, Matt Hurles, Michael Simpson, Miriam Samuel, Mohammed Bodrul Mazid, Moneeza K Siddiqui, Nishat Safa, Omar Asgar, Panos Deloukas, Raymond Chung, Richard C Trembath, Rohini Mathur, Sabina Yasmin, Saeed Bidi, Sam Hodgson, Samina Ashraf, Sang Hyuck Lee, Sarah Finer, Shaheen Akhtar, Shabana Chaudhary, Shapna Hussain, Sheik Dowlut, Stuart Rison, Teng Heng, Vladimir Ovchinnikov, Vivek Iyer, and Jan Whalley. https://docs.google.com/spreadsheets/d/1D9HLbc_m0KdOUN-gS0hymTLewJ36e8ETSEb8Tu_CqWY/edit?gid=0#gid=0.

## Acknowledgments

The acknowledgments are provided in the supplemental information.

## Author contributions

T.H.H. helped plan the project, performed the data analysis, and drafted the manuscript. K.W. supervised the association testing. Q.Q.H. supervised the QC and, with D.A.v.H., performed the TOPMED-r2 imputation. J.K. and M.J.D. ran the recessive testing in FinnGen. H.H. advised on FinnGen data and recessive association testing. D.M. contributed to the power and F_ROH_ calculations. G.K. advised on imputation and association testing. D.A.v.H. supervised the collection and curation of G&H data and advised on association testing. H.C.M. planned and led the project and helped draft the manuscript. All authors reviewed the manuscript.

## Declaration of interests

The authors declare no competing interests.

## Web resources

FinnGen, https://r6.finngen.fi/
Genes & Health, https://www.genesandhealth.org/researchers/
UK Biobank, http://www.nealelab.is/uk-biobank/

## Supplemental information

Document S1. Figures S1–S18, Tables S1–S4, S6, S7, S10 and Notes S1–S12

Data S1. Tables S5, S8, S9, and S11

Table S12. FinnGen consortium members

Document S2. Article plus supplemental information
