# Supplementary material for: Widespread recessive effects on common diseases in a cohort of 44,000 British Pakistanis and Bangladeshis with high autozygosity
Source: Am J Hum Genet. 2025 Apr 29;112(6):1316–29. doi: 10.1016/j.ajhg.2025.03.020 (PMC12256797; doi:10.1016/j.ajhg.2025.03.020)
Supplement: Document S1. Figures S1–S18, Tables S1–S4, S6, S7, S10 and Notes S1–S12 [file mmc1.pdf]

The American Journal of Human Genetics, Volume *112*

Supplemental information

Widespread recessive effects on common diseases in a cohort of 44,000 British Pakistanis and Bangladeshis with high autozygosity

Teng Hiang Heng, Klaudia Walter, Qin Qin Huang, Juha Karjalainen, Mark J. Daly, Henrike O. Heyne, FinnGen, Daniel S. Malawsky, Georgios Kalantzis, Genes & Health Research Team, Sarah Finer, David A. van Heel, and Hilary C. Martin

Supplemental notes

Note S1: The statistical power needed for recessive analyses

We ran simulations to show that power to detect a recessive effect can be boosted firstly by fitting a recessive rather than an additive model, and secondly by increased homozygosity in the cohort.

We used the genpwr R package [1] for the simulations. We applied the following parameters for all calculations: sample size = 44,000 (mimicking G&H), p-value threshold = $p<5x10^{-8}$, model = recessive (or additive), regression = logistic, and assumed there was no gene-environment interaction. The power, allele frequency (AF), case rate and odds ratio (OR) were modified depending on what was being simulated. Since the package does not accept genotype frequencies, but rather, takes the AF as input and assumes the Hardy-Weinberg Equilibrium (HWE) to determine the genotype frequencies, to account for increased autozygosity in G&H, we calculated the frequency at which one would expect to see the number of homozygotes that we would actually see under HWE, denoted below as “$AF_{autozyg}$". Specifically, we calculated:

$$AF_{autozyg} = \sqrt{(1 - F) * AF_{out}^2 + F * AF_{out}}$$

AF_{out}: AF of the $variant$ in a $hypothetical$ $population$ at HWE

$F = The\ average\ inbreeding\ coefficient\ in\ the\ sample$

The parameter F (commonly known in the scientific literature as the “inbreeding coefficient”) corresponds to the average relatedness of parents of individuals in the sample with each other. It also corresponds to the average fraction of the genome homozygous in a given sample. [2]

We assessed if we would have power to detect recessive findings with similar ORs, AFs and case rates as those detected in FinnGen. The FinnGen recessive hits were collated from Table 1 of Heyne et al. (2023) [3]. We filtered the thirty-one findings to the eighteen findings (Table S3) that were validated in release 6 [4]. The ORs, AFs and case rates were then projected into the power calculations we performed.

Using an OR of 2 and a phenotype case frequency of 1%, we calculated the power of a recessive versus an additive test across the AF spectrum, simulating various levels of average autozygosity in the cohort, ranging from a cohort with no autozygosity (F = 0), to a consanguineous cohort with an average inbreeding coefficient of 10% (which corresponds to the average fraction of individuals homozygous at any position in the genome). In practice, the average fraction of the genome homozygous in individuals from G&H is ~2.2%. We first see that the additive model had less power for detecting a truly recessive effect than the recessive model, particularly at lower AFs (Figure S1A,B). Next, we see that power to detect this recessive effect increased with the average level of autozygosity, and for this set of parameters, this was most noticeable around the AF 0.2 - 0.5 range (Figure S1A). This sample size and levels of consanguinity simulated were not powered to detect an effect size of OR = 2 in rare variants, therefore we increased the OR to 5 and reran the simulations for

the rare and low frequency spectrum, and we still saw that power is higher with increasing F (Figure S1B).

We next wanted to evaluate the minimum OR we would be powered (80% power) to detect in G&H. To do that we simulated varying case rates from 0.1% to 25%. We projected the recessive findings reported in FinnGen by Heyne et al. (2023) onto our simulations, and found that the majority of the findings had ORs higher than the minimum that we are powered to detect at their corresponding case rates and AFs (Figure S1C). This suggested that G&H will be well powered to detect very large ORs at rare variants i.e. effectively Mendelian associations, and smaller recessive effects at common variants with common traits.

Note S2: QC of genetic data

Within this cohort, exome sequencing was performed using Agilent V5 capture kits on a subset of 5,236 G&H individuals who self-declared as having related parents following the protocol described in Narasimhan et al. (2016) (Agilent SureSelect Human All Exon V5, [5]). Sequencing was performed in batches, resulting in a bimodal distribution of read depth, with some individuals being sequenced to ~40X, and the rest ~20X (Figure S7A). Mapping was performed with the Burrow-Wheeler Aligner (specifically the BWA-MEM algorithm) [6]. Variants were then called using the GATK HaplotypeCaller [7] and annotated with Ensembl Variant Effect Predictor v95 [8]. This was released on September 2019: (https://www.genesandhealth.org/research/scientific-data-downloads/sept-2019-summary-files-exome-sequencing-loss-function-variant)

Subsequently, QC of the WES data relevant to this project was performed, as summarised in Table S1 below.

Note S3: Genetic inference of ancestry and analysis of relatedness

Ancestry inference

We genetically inferred ancestry for the 44,396 individuals by merging the SNP-array data with reference cohorts.

From the SNP-array had been through initial QC, we filtered to variants that were autosomal, common (defined as a minor allele frequency, MAF>0.01), had a call rate of ≥99%, and passed the HWE exact test ($p>10^{-6}$) in self-declared Bangladeshi individuals (Table S1). This filtered dataset was then merged with reference sequences of 3,433 individuals from the 1000 Genome Project (1000G) [9] and Central and South Asian individuals from the Human Genome Diversity Project (HGDP) [10]. We excluded palindromic variants, and variants with significant AF differences between G&H and 676 reference South Asians curated from 1000G and HGDP (since these represented likely genotyping errors). We defined variants with significant AF differences in the following manner: Firstly, we calculated the residuals from the linear regression between the AFs in both datasets. Next, we binned the variants into bins by frequency (in intervals of 0.01), and selected variants for which the residual was >5 standard deviations (5SD) away from the mean of the residuals in that frequency bin. (This choice seemed reasonable after testing various SD thresholds, Figure S3A). Lastly, we performed Fisher's exact tests to compare the genotype counts between the G&H data and the 676 reference South Asians at the variants selected above and excluded those with a $p<10^{-5}$. (Again,

we tested p-value thresholds ranging from 0.05 to a multiple testing correction of <0.05/349,632 variants, and felt that 10^{-5} was reasonable, Figure S3B). The various thresholds and the distribution of the outlier variants excluded are graphically represented in Figure S3C-D.

After merging of G&H with the reference samples, LD pruning was performed (window size 1000 kilobases (kb), step size 50, LD r^2 0.1) with PLINK1.9 and long LD regions were excluded. [11]

Principal component analysis (PCA) was performed with PLINK1.9 on the reference individuals, then the G&H individuals were projected into the reference PC space (PCA1). We calculated uniform manifold approximation and projection (UMAP) coordinates (umap R package) [12]. We found that the UMAP with 7 PCs was optimal to separate the reference individuals into superpopulations. 44,320 out of 44,396 G&H individuals were inferred to be South Asian at this stage (Figure S4A), and carried forward for the downstream analysis.

The PropIBD algorithm in KING [13] was run to estimate pairwise relationships up to fourth degree within G&H, and we removed a minimal set of 14,727 individuals who had at least one relative (3rd degree and closer) in the dataset, leaving 29,668 unrelated individuals.

We performed a second PCA (PCA2) on the unrelated G&H individuals, projecting the related G&H individuals who we had inferred to be South Asian into the PC space. The UMAP with 4 PCs identified distinct clusters that

corresponded well to self-declared Bangladeshi/Pakistani ancestry, and this was used to genetically classify individuals as genetically Bangladeshi or Pakistani (Figure S4B).

Relatedness

Next, we attempted to estimate the number of discrete families from the KING relationship inference (derived from the pairs defined in the '.kin0' output of the command KING –related –degree 3 (using PropIBD)). We identified 35,500 pairs involving 25,920 unique individuals that are inferred to be third degree relatives or closer: 7 identical twin pairs, 6,595 parent-offspring pairs, 5,863 full-sibling pairs, 11,639 second-degree pairs, and 11,396 third-degree pairs. From this, we estimated that these relationships cluster into 5,742 discrete families, with an additional 18,270 individuals with no inferred relationship (3rd degree or closer) to others in the cohort. Given the difficulty in accurately inferring more distant relationships in this endogamous population, we also inferred families considering only first degree relatives, namely parent-offspring and full-sibling pairs. In the cohort, 16,122 individuals have at least one first degree relative, and they were grouped into 6,122 families.

To investigate whether homozygotes are clustered within the same families, we focused on 52 rare variants (MAF <1%) with recessive associations and took all pairs of individuals who carry the same homozygous rare variant. Among the 848 pairs of such individuals, two were inferred as parent-offspring pairs, nine as full siblings, and three as 2nd degree relatives, while the remaining pairs were more distantly related than 3rd degree. In contrast, randomly-selected pairs are less likely to be related, within only one 2nd degree relative observed

in 100 simulations of 848 random pairs. This suggests that, as one would expect, homozygous carriers of rare variants that showed significant recessive associations are indeed more likely to be from the same family, especially as full siblings (who share a quarter of their genome IBD2), compared to randomly selected individuals. However, the majority of these homozygous carriers (98%) are more distantly related than 3rd degree, and we believe that the mixed-effect models implemented in REGENIE should sufficiently account for relatedness in association testing. Indeed, when we reran the association analysis in unrelated individuals (i.e. all pairwise relationships are more distant than 3rd degree), we found good correlation between the -log10p values from the full cohort and the unrelated subset (n = 26,579 in the largest set), though unsurprisingly there was less power (linear regression slope = 0.6, r2 = 0.93, Figure S5).

Note S4: Assessing genotyping and imputation accuracies with concordance analyses

Before merging the SNP-array and WES data to build the reference panel, we evaluated concordance of genotypes between them at overlapping sites. Furthermore, after imputation, we assessed imputation accuracy by comparing imputed genotypes to sequenced genotypes.

Evaluating concordance

Concordance was primarily evaluated by the non-reference discordance rate (NRD) calculated using the following formula:

$$NRD = \frac{xRR + xRA + xAA}{xRR + xRA + xAA + mRA + mAA}$$

$$R : reference\ allele^*$$

$$A : alternate\ allele^*$$

$$x : nMismatches$$

$$m : nMatches$$

*Note that the ‘truth’ was defined by the array genotype when comparing concordance between array and WES data, and defined by the sequenced genotype when comparing concordance between sequenced and imputed data. For example, “xRR” would mean that the truth dataset had a homozygous

reference genotype while the other dataset did not, and “mRA” means that both datasets had a heterozygous genotype.

When examining NRD stratified by allele frequency, the NRD was modified to calculate a “minor allele discordance” (MAD) rate:

$$MAD = \frac{xMajMaj + xMajMin + xMajMin}{xMajMaj + xMajMin + xMajMin + mMajMin + mMinMin}$$

$$Maj : major\ allele^{*}$$

$$Min : minor\ allele^{*}$$

Where relevant, Pearson correlation r^2 between genotypes / dosages were calculated as a secondary measure of concordance.

Concordance between the SNP-array and the WES data

QC on both the SNP-Array and WES datasets improved the overall NRD from 5.02% to 0.40%, with the WES GQ filter making the biggest difference (Table S2). We anticipated that the genotyping accuracy would be low for rare variants in the SNP-array, but surprisingly the array data continued to demonstrate good MADs of ~0.7-0.9% with the WES for variants with minor allele counts (MACs) on the array of 3-6 (equivalent to MAF 0.03-0.06%, Figure S6). We decided to use variants with MAF>0.1% from the SNP array for the imputation backbone.

Since minimac3 (Which is used to build the reference panel for imputation with minimac4) does not tolerate missingness in the imputation reference panel, we needed to use the WES data without any genotype-level QC to minimise

missingness. To select for variants with a majority of high-quality genotypes, we retained those that had <30% missing genotypes after applying the genotype-level QC. Using the raw genotypes at those sites increased the NRD to 1.26% (Table S2). Note that this value is inflated by the discordance at sites with MAC 0-2 (Figure S6), but variants this rare were filtered out of the SNP-array when building the imputation backbone, and instead retained in the WES data, since we assume these are likely to be more accurate than the array genotypes given the difficulties of genotyping uber-rare variants on arrays [14].

Based on the concordance analysis, we decided to include only array variants with MAF>0.1% in the imputation backbone. Overlapping positions with the WES were resolved as follows:

- For SNPs at overlapping positions with matched alleles and with MAF>0.1%, we retained these in the GSA data but removed them from the WES.
- We excluded overlapping common palindromic variants (MAF>0.4) where the strand could not be confidently determined.
- We excluded overlapping positions with unmatched alleles (including all indels).

This resulted in 469,678 variants, which were then phased with EAGLE2 (Kpbwt=20,000) [15].

Based on the concordance of the SNP-array and WES, 91 samples that had high missingness and/or high non-reference discordance (NRD) values were

excluded (Figure S7B, Figure S7C), leaving 4,982 samples. Specifically, we excluded individuals who had any of the following:

- a raw WES call rate <4SD from the mean raw WES call rate,
- a post-genotype QC WES call rate <2SD from the mean post-genotype QC WES call rate,
- an NRD based on the post-genotype QC WES and post-QC SNP-array data of >4SD from the mean

To build the reference panel, we filtered the WES data to a subset of high-quality variants that had ≥70% call rate after genotype-level QC, and removed singletons. Since Minimac3 does not cope with missing genotypes, we used the genotypes at those sites from the raw data (i.e. pre-genotype QC), and replaced missing genotypes (~0.02% of the total genotypes, Figure S8A) with 0/0 if the reference allele was the major allele and 1/1 otherwise. The distribution of raw per-variant missingness at sites that pass a post-genotype QC call rate ≥70% is shown in Figure S8B, with most variants having a raw missingness of <1%.

Variants with MAF>0.1% that were also present on the GSA array and had been included in the the imputation backbone were removed from the reference panel, since they tended to have higher call rates in the array data. The cleaned WES and cleaned SNP-array data from the 4,982 samples were then merged to form the reference panel and phased with EAGLE2 (Kpbwt=20,000). The reference panel consisted of 1,385,942 variants.

Evaluating imputation accuracy

After imputation, to assess the imputation accuracy, the imputed genotypes for the 4,982 individuals with WES data were compared to their sequenced genotypes. Genotype concordance was evaluated between the SNP-array and the WES data at overlapping sites, and between the sequenced and imputed variants.

Ten trials of imputation were performed using the WES5K panel, with each trial leaving 10% of the WES samples out of the reference panel against which we then evaluated concordance between the sequenced and imputed genotypes.

The total number of variants in each imputed dataset ranged from 1,325,855-1,327,697 across the 10 trials. Although the genotyped backbone is imputed by Minimac4 as well, to evaluate imputation accuracy purely at positions with no prior information, we excluded backbone SNPs resulting in 855,697-857,537 variants to compare in each trial. The overall NRD of the imputed genotypes compared to the sequenced genotypes ranged from 7.44-7.69% across 10 trials (Figure S9A).

The confidence of the imputation is quantified by Minimac4's imputed R^2. By convention, a minimum cutoff of ≥0.3 is applied to QC imputed data, which should sufficiently filter out poorly-imputed variants based on the distribution of imputed R^2 scores (Figure S9B). However the overall NRD was not found to improve at this cutoff (Figure S9A) (probably because we are including so many rare variants) so we applied a more stringent cutoff of ≥0.5. As expected, the rarer MAF bins had more variants with a lower imputed R^2 (Figure S9C).

MAD also increases with decreasing allele frequencies, as expected. Many of these extremely rare variants will not be submitted for association testing as they are both poorly imputed and not powered enough for recessive tests.

After applying imputed R^2 ≥0.5 and number of homozygotes (N_{Hom}) ≥3 to prepare for association tests, and including the positions of the SNP-array backbone (which will be included in association tests), the overall NRD improved to 1.78-1.84% across the trials (Figure S10).

The TOPMEDimputation genotypes of the 4,982 samples with WES were compared to their sequenced genotypes. The same cutoffs (imputed R^2 ≥0.5 and N_{Hom} ≥3) were applied to result in 10,045,406 variants. Of these variants, 523,018 variants overlapped with the WES and at these, the overall NRD was 1.19%.

We then considered NRD at variants stratified by the number of homozygotes as defined in the WES5Kimputation, the rationale being that the WES should be considered “truth” in this case, and that the power of the recessive association testing depends on the number of homozygotes rather than directly on the allele frequency. First, we observed that the within-cohort WES5K reference panel allowed for more variants to be imputed at the chosen level of accuracy, especially at lower MAFs (Table S3). Second, for N_{Hom} ≥9 up to a homozygous frequency of 5%, the MAD is lower for the WES5KImputation compared to the TOPMEDImputation, but the opposite is true for the lowest and highest MAF bins. Thirdly, the lower overall NRD of the TOPMEDimputation (1.19% versus ~1.7% for the WES5K Panel) is driven by the improved accuracy of common

variant imputation with the TOPMED reference panel. (Figure S10) As both imputation sets carried their own strengths and weaknesses across the frequency spectrum, it was decided to bring both sets forward to association testing.

Note S5: On multiple testing and the independence of phenotypes

The 898 phenotypes tested are not completely independent of each other, and there may be significant correlation between phenotypes, particularly as some diseases are repeated in the custom list and the ICD10 codes. We sought to calculate Pearson correlation r^2 between phenotype pairs to quantify the degree of correlation in our phenotypic data. This serves two purposes - to evaluate if phenotypes we would expect to be highly correlated indeed have a high r^2 (meaning that they have been curated correctly), and secondly, to try to eliminate highly-correlated phenotypes to reduce the multiple-testing burden.

Of the 402,753 pairs of phenotypes generated, 153 pairs had a correlation r^2 of ≥ 0.5. Manually inspecting these pairs, the majority were between conditions one would expect to be highly correlated; for example, the ICD10 encoding for sarcoidosis and for multiple sclerosis fully correlated ($r^2 = 1$) with the respective custom encodings for these conditions. There were also correlations between biologically similar phenotypes, such as pulmonary heart disease and pulmonary hypertension ($r^2 = 0.89$), and correlations between pairs for which one trait was a subset of the other, such as acute pancreatitis and pancreatitis ($r^2 = 0.90$). By reviewing these highly-correlated pairs, we estimated that only about 80-90 phenotypes could be excluded due to being highly correlated, as the rest of the pairs had differences in their definitions that warranted the inclusion of both phenotypes.

We therefore tested all 898 phenotypes available, and to be stringent, the Bonferonni cutoff we used accounted for all tests as if they were independent.

The number of phenotypes, individuals, and variants tested is summarised in Table S4. A small minority of tests failed on REGENIE; a total of 9,197,933,046 tests produced a p-value, out of a possible 9,374,352,233 tests (98% success rate).

Note S6: On covariates included in the association testing

The covariates included were age (at year of phenotype curation, 2022), sex, age^2, age x sex, age^2 x sex and the first ten principal components (PCs) from the principal component analysis on unrelated G&H individuals described above.

We controlled for ten genetic PCs derived from common variants. These top ten PCs explained more than 85% of the variance explained by the top 50 PCs (Figure S11A).

For the 56 out of 185 recessive loci that involved rare variants (AF <1%), we further investigated if the common PCs were adequately controlling for population structure by checking that the results held when additionally controlling for PCs based on an IBD sharing matrix that should capture more subtle, recent population structure. We used KING to call IBD segments and calculate the length of IBD sharing across pairs of individuals in the cohort, and performed a PCA on the matrix of IBD sharing. We then repeated the association analyses for the 56 recessive loci involving rare variants, adding the first 15 PCs from the IBD PCA to the existing covariates (since these explained most of the variance in the PCA; Figure S11B). Reassuringly, the betas (i.e. the effect sizes, linear regression slope 1.03, r2 0.98, p-value $<2x10^{-16}$, Figure S11C), and the p-values (converted to -log10p, linear regression slope 0.67, r2 0.25, p-value $5.4x10^{-5}$, Figure S11D), of the associations correlated well between the association tests performed controlling for IBD PCs and the

association tests performed controlling for common PCs, with the exception of a single outlier.

Previous work in the lab by [16] demonstrated that increased runs of homozygosity (ROHs) in the genome was associated with several conditions such as anxiety and type 2 diabetes. We sought to explore if the fraction of the genome in ROHs (F_{ROH}) is a possible confounder for these recessive findings, by adding it as a covariate and rerunning the association tests for the 42 lead SNPs in the WES5Kimputation dataset. ROHs were calledby PLINK1.9 on the SNP-array data with the following specifications: maximum inverse density 50kb/SNP, maximum internal gap 1000kb, minimum SNP count 50, maximum 1 heterozygous in scanning window hit, maximum 4 missing calls in scanning window hit and a scanning window sizeof 50. The total length of ROHs (in kb) was then divided by the length of the autosome (approximately 2700000kb) to obtain F_{ROH}. After controlling for F_{ROH}, the recessive p-values correlate well with those obtained without controlling for it, suggesting that this additional covariate is unnecessary (Figure S12).

Note S7: Characteristics of loci identified as significant in the recessive association testing

Information about the 185 lead variants passing $p<5x10^{-8}$ are tabulated in Table S5. The frequency and consequence distributions of the lead variants are shown in Figure S13. Despite applying an exome reference panel including many rare protein-coding variants, many of the findings from the WES5K imputation were still common and intronic, implicating variants near exonic regions that happened to be captured with WES, or common SNPs from the GSA backbone. Similarly, the majority of the significant hits from the whole genome TOPMEDimputation were within the non-coding regions. This is expected as common variants have better power and 99% of the human genome is non-coding.

As an example for plotting and visualisation purposes, we plotted the recessive tests performed between the TOPMEDImputation and D58[Other hereditary haemolytic anaemias]. The Quantile-quantile (QQ) plot (Figure S14A) suggests that the tests are underpowered, as we see substantial deflation of the test statistics below what is expected under the null (lambda = 0.56). When we split the variants contributing to these tests into common (AF > 5%) and low frequency variants (AF ≤ 5%), indeed we see that the lambda of 0.94 for the common variants is close to 1 (Figure S14BC), while the lambda for the rare variants is low at 0.21 (Figure S14C), indicating that the deflation is likely due to reduced power for rare variants.

Next, we tested whether significant recessive associations were more likely to be coding variants than non-coding ones. In our association testing, we evaluated 10,045,406 variants that were imputed with TOPMED, among which 91,930 were coding variants, and identified 1,216 variants with significant recessive associations with any phenotype. We observed significant enrichment of recessive associations amongst coding variants (Chi-square test P-value = 8.3×10^{-20}), with 0.044% having significant associations compared to 0.011% of non-coding variants. As expected, given the coding regions only cover ~1% of the genome, the vast majority of significant recessive effects we detected are non-coding (96.5%). However, the significant recessive associations are enriched among coding variants compared to the total set of variants tested for association: 3.5% of recessive variants are coding while only 0.9% of all tested variants are coding, as shown in Table S6.

To assess whether the enrichment varied by MAF, we stratified the analysis by MAF bins. We observed significant enrichment for coding variants amongst common variants (MAF > 5%; P-value = 2.3×10^{-16}) and low-frequency variants (MAF 1–5%; P-value = 2.4×10^{-8}) but not in rare (MAF 0.1–1%; P-value = 0.66) or ultra-rare variants (MAF < 0.1%; no coding variants reached significance in recessive testing). The enrichment analysis for the rare and ultra-rare groups is likely underpowered, since only 94 and 9 variants, respectively, showed significant recessive associations. These findings are consistent with the expectation that protein-coding variants are more likely to impact gene function and health outcomes, and that they have larger effect sizes leading to better power for detection.

Note S8: Evaluating the dominance deviation of the 185 recessive findings

For the lead variants of the 185 recessive loci, 152 lead variants were not GWS in the additive test. Looking at the distribution of AFs, recessive hits that were GWS in the additive test had higher AFs than hits that were insignificant in the additive test, suggesting that common variants simply had more power to be detected under the additive model even if the underlying pattern of inheritance might be recessive. (Figure S15A).

For the lead variants of the significant recessive associations, we also reran the additive test (step 2 of REGENIE) after removing homozygous individuals, to explore heterozygous effects. When doing this, all but two lead variants had p-values below GWS, demonstrating both the weight of these homozygotes on the significant results in the original additive tests and the loss of power by reducing the sample size of the tests. The remaining two lead variants likely have strong heterozygous effects that can be detected even at reduced power. (Figure S15D)

It is possible that the tests that dropped below GWS after homozygotes were removed may still have heterozygous effects. We compared the betas in the different models of testing. As expected, additive tests estimated betas that were smaller in magnitude compared to the recessive tests (Figure 2B). After dropping the homozygotes, 107 of these tests became insignificant (p-value > 0.05), but for tests that remained nominally significant, their betas correlated well with the full additive tests, though again, their estimated effect sizes were

smaller (Figure S15E). These nominally-significant additive tests performed without homozygous individuals demonstrate heterozygous effects despite reduced power.

When we removed the homozygous individuals for the additive tests, a small minority of tests (18/185 lead variants) could not be run in REGENIE when the homozygotes were excluded. These tests tend to have higher AFs greater than 0.7 (suggesting that the removal of homozygotes resulted in the test being too underpowered) (Figure S15B).

To further explore whether the recessive model was indeed the best fit for the variants that were significant on the recessive test in REGENIE, we performed logistic regression testing in R, using a genotypic model that included a dominance deviation encoding. For comparison, we also fitted a standard additive and recessive model in R. We re-coded the genotypes to perform additive, recessive and genotypic tests with 2 degrees of freedom, as shown in Table S7 and the equations below.

<u>Additive test:</u>

$$phenotype \sim \beta_{add} G_{additive} \; + \; BC$$

where $G_{additive}$ is the genotype of the SNP using the additive encoding (0/1/2), β_{add} is the effect size under an additive model, C is a matrix of covariates (defined below) and B is a vector of effect sizes for those covariates.

<u>Recessive test:</u>

$$phenotype \sim \beta_{rec} G_{recessive} + BC$$

where $G_{recessive}$ is the genotype of the SNP using the recessive encoding (0/0/1), and β_{rec} is the effect size under a recessive model.

<u>Genotypic (2 degrees of freedom) test, to extraction dominance deviation:</u>

$$phenotype \sim \beta_{add} G_{additive} + \beta_{domdev} G_{domdev} + BC$$

where G_{domdev} is the genotype of the SNP using the dominance deviation encoding (0/1/0), and β_{domdev} is the effect size of the dominance deviation under the genotypic model.

The tests were performed on the full cohort (i.e. the set of individuals used in REGENIE, sample sizes in Table S4), as well as on the subset of individuals genetically-inferred to be unrelated by KING (26,579 individuals in the largest cohort, subsetted accordingly depending on the imputation and phenotype tested).

<u>Covariates</u>

The covariates included were age, sex, age^2, age x sex, age^2 x sex and the first ten PCs.

In Figure S16, the results from the additive and recessive tests in R were plotted similarly to those in REGENIE in Figure 2 and gave similar conclusions to those noted earlier in the main text.

Before considering the results from the dominance deviation tests, we compared the results from the standard recessive model between R logistic regression and REGENIE. We found that the -log10(p-values) correlated well (Figure S17A, linear regression slope = 1.1, r^2 = 0.96). This correlation still held when restricting to a set of 26,579 unrelated individuals (in the largest set) in R, though unsurprisingly there was less power (linear regression slope = 0.6, r^2 = 0.93). Still, there were some REGENIE tests that were not significant (p-value > 0.05) in the R logistic regression, and these outliers had much larger effect size estimates in R (Figure S17B). Otherwise, for tests that were nominally significant in R, their betas correlated well with REGENIE (linear regression slope = 0.98, r^2 = 0.98). The outlier R tests that had insignificant p-values tended to involve rarer variants (Figure S17C, Wilcoxon two-sided p-value = 2×10^{-4}), although the distribution of phenotype case counts were similar (Figure S17C, Wilcoxon two-sided p-value = 0.27). They were excluded from subsequent analyses described below.

When fitting the genotypic model, 76% (or 140) of these lead variants had nominally significant dominance deviation p-values. These variants tended to be at least nominally significant in the R recessive test as well (chi-square test p-value = 1.3×10^{-11}, Figure S17D). For the tests that had an insignificant dominance deviation p-value, we cannot rule out that the underlying inheritance pattern may still be recessive, especially as some also had recessive tests with

insignificant p-values in R, suggesting that R is an imperfect model to replicate the results from the more complex model fitted by REGENIE.

Regardless, as expected, the recessive and dominance deviation p-values were correlated (linear regression of their log10-transformed p-values: slope = 4, $r^2 = 0.3$, p-value $<2.2 \times 10^{-16}$) (Figure S17E), with hits having a nominally significant dominance deviation tending to have lower recessive p-values (Wilcoxon two-sided p-value = 2.8×10^{-5}). We also saw that hits having a nominally significant dominance deviation also tended to have a larger difference between their recessive and additive p-values (Wilcoxon two-sided p-value = 3×10^{-9}) (Figure S17F). We did not find any difference in the recessive betas, distribution of AFs and case counts between the hits with nominally significant and insignificant dominance deviation p-values (Wilcoxon two-sided p-value 0.3, 0.7 and 0.2 respectively, Figure S17G-H).

In summary, from removing homozygotes and re-performing the additive tests, we demonstrated that several of our recessive findings may harbour mild heterozygous effects. In addition, fitting the genotypic model in R provided further support for at least three-quarters of our findings being truly recessive.

Note S9: Replication in other cohorts

Table S7 and Table S8 list the Genes & Health phenotypes being matched to FinnGen phenotypes and GERA phenotypes respectively.

The current locus definition (r^2>0.25 and within 1.5Mb of the lead variant) was chosen so as not to inflate the pairs of independent findings we report, but for the purpose of replication in other cohorts, they are not as stringent as the one described in Huang et al. 2022. For completeness, we repeated the replication calculations with the Huang et al. 2022 cutoffs, so as to calculate a PAT in a manner closer to what was described in the literature. For each significant locus in G&H, we first identified proxy variants as variants that are within a +/-50kb window from the lead variant with LD r^2 ≥0.8, and a p-value ≤100 times the p-value of the lead variant. The PAT in FinnGen reduced from 23% to 21% for genome-wide significant loci with this more stringent locus definition. Notably, the PAT for the Bonferroni-significant loci increased from 44% to 56%, perhaps reflecting the complex LD patterns in the chromosome 11 thalassemia and hereditary anaemia findings, and how different locus definitions would therefore affect the counting of the number of independent loci for these associations.

Additionally, we have performed PAT calculations while varying the replication criteria, trying various more stringent cutoffs, such as replication only by the lead variant, and considering a p-value cutoff of ≤0.01 or ≤0.05/number of tests in the replication cohort. We have presented the results of these different trials in Table S10. Firstly, at our original replication p-value threshold of 0.05, the percentage of loci that replicate by their lead variant alone was 53% for

genome-wide significant variants and 57% for Bonferroni significant variants. Next, at a replication p-value cutoff of ≤0.05/number of tests, the PAT for genome-wide-significant loci decreased from 23% to 14%, and the PAT for Bonferroni-significant loci changed to a smaller extent from 44% to 38%. Broadly, stricter definitions for replication do reduce the number of loci we have replicated, and this affects the genome-wide-significant findings more than the Bonferroni-significant findings.

Note S10: Systematically finding literature support for the recessive associations and quantifying the novelty in our findings

We attempted to identify literature support for the 185 recessive loci in our study with a systematic approach. In addition, we aimed to use the findings from this to quantify the novelty in our findings.

Additive analysis in external datasets

Firstly, we searched external, publicly available datasets, which are mostly additive tests. From 8 October 2024 to 21 October 2024, we searched each lead variant on genetics.opentargets.org. The portal compiled associations between variants and phenotypes reported in FinnGen, UK Biobank and the GWAS Catalog. Different datasets applied different p-value thresholds, and the highest p-value reported in the data within this analysis was 0.0049.

We tabulated relevant associations in Note S11, and assigned a "Literature.Evidence.Score" to the evidence found:

- 9 (None): Variant not found
- 0 (None): Variant is found, but no reported associations with related traits
- 1 (Weak): Association with 1 related trait in 1 dataset. FinnGen and UK Biobank are each defined as 1 dataset. The GWAS Catalog is also defined as 1 dataset (With several exceptions and caveats: Evidence in the GWAS catalog can come from different studies. Some GWAS results may have used UK Biobank or FinnGen as well. Therefore the specific

studies contributing to the scoring have been listed in Note S11 and can be further reviewed.)

- 2 (Weak): Association with 1 related trait in more than 1 datasets.
- 3 (Moderate): Associations with more than 1 related traits, in 1 dataset.
- 4 (Moderate): Associations with more than 1 related traits, in 2 datasets.
- 5 (Strong): Associations with more than 1 related traits across all 3 datasets.
- 6 (Strong): At least 1 association with the same trait in any of the 3 datasets (with or without associations with 1 or more related traits across 1 or more datasets).

We acknowledge that there is some subjectivity in deciding whether a trait is related, and whether different entries with different variations on a trait name count as different traits or the same trait. However, this would be a persistent concern with any attempt at phenotype matching.

Of the 185 lead variants, 166 (~90%) could be found in the Open Targets portal. The remaining ~10% of lead variants that were only reported in G&H tended to be rarer (ANOVA across all seven scores p-value 0.02, one-sided Wilcoxon between variants with score 9 compared to the others p-value 4.0×10^{-5}). This was expected as variants unique to G&H are likely to be rarer, and further supports the value of performing such genetic analyses in diverse cohorts. In addition, among the variants that could be found in external datasets, increasing AF was associated with stronger evidence (linear regression slope 0.036, r^2 0.05, p-value 0.003). This was also expected, as variants with higher

AF would have better power to be significant with the additive model even if the underlying pattern of association is recessive. (Figure S18A)

Of the 166 loci that could be evaluated in external datasets, 35 had strong support in the literature (27 with associations to the same trait, 8 with associations to multiple related traits across multiple datasets), 28 had moderate support (associations to multiple related traits), 35 had weak support (association to a related trait), and 68 had no associations to any related traits. Arguably, all but the 27 loci with reported associations to the same trait (158 loci remaining, 85%) are novel. With a stricter definition of novelty (i.e. if we define a novel locus as a locus with no reported associations with related traits), 87 loci (19 variants not found, 68 variants found but with no reported association with related traits), or ~47% of our findings, can be considered novel. As the FDR5 threshold of $3.7x10^{-8}$ is similar to the genome-wide-significant threshold of $5x10^{-8}$ we have used here, we can assume that these numbers are inflated by ~5% (assuming that all the false positives are amongst the ‘novel’ set), and that an estimated 42-80% of our findings are novel.

Interestingly, there was no difference in the distribution of recessive p-values across the 8 scores (ANOVA of -log10p across all scores p-value 0.87) (Figure S18B). Instead, the difference between the additive p-value and the recessive p-value (delta log10p) was associated with the strength of the evidence; the smaller the delta log10p, the higher the evidence score (linear regression of delta log10p across scores 0-6 slope -0.32, r^2 0.09, p-value $5.4x10^{-5}$) (Figure S18C). This implied that it was not the magnitude of the recessive p-value that was suggestive of whether the association had been detected before in additive

testing, but the difference in the p-values between the additive and recessive test that was more predictive. This makes sense, as it is possible that many of the recessive associations we report here were missed by other studies that performed additive testing alone, and in cohorts that did not have sufficient homozygous individuals to power recessive analysis.

Additive analysis of quantitative traits in G&H

Next, we also searched the summary statistics of the quantitative traits GWAS performed in G&H [17], which we downloaded on 8 October 2024. We searched for the lead variants representing the 185 loci in the summary statistics of the 42 quantitative traits tested. There were 242 associations at a nominal significance p-value cutoff 0.05. We then assigned a "Quantitative.GH.Score" to each loci:

- 0 (None): No significant associations to related quantitative traits
- 1 (Moderate): Significant associations to related quantitative traits
- 2 (Strong): Significant associations to quantitative traits directly involved in the diagnosis or presentation of the binary trait

(Again, we acknowledge that there is some subjectivity when deciding whether a quantitative trait is related to a binary trait.)

Of the 185 recessive loci reported in our study, 19 (~10%) had a score of 2, meaning that the lead variant had significant associations with quantitative traits directly related to the binary trait. Notably, 10 of these loci relate to the chromosome 11 associations with thalassaemia and hereditary anaemias, and

they were all significantly associated with red blood cell derangements related to anaemia (Note S11), which was expected.

An additional 14 loci (~8%) had a score of 1, demonstrating significant associations to quantitative traits that were related to the binary trait. They provide insight into possible underlying mechanisms that lead to the association between the variant and the binary trait. For example, we reported a recessive association between the variant chr1:31215264:T:C and increased likelihood of seborrhoeic dermatitis (recessive OR 1.3 and p-value 2.8×10^{-9}), and the additive GWAS reported an association between the variant and decreased vitamin D levels (beta -0.027 and p-value 3.5×10^{-2}). There is further evidence in the literature of decreased vitamin D levels being associated with seborrhoeic dermatitis [18–20], suggesting that vitamin D may play a role in the pathophysiology of seborrhoeic dermatitis. Indeed, this relationship has been hypothesised to be due to vitamin D's role in the inflammatory cascade [18,20,21]. As mentioned in the main text discussion, the liability threshold model is often applied to model binary traits. This finding provides additional support for this model - it is possible that this variant has an additive effect on vitamin D levels, and after a certain threshold, the vitamin D deficiency becomes disease-causing, and therefore the variant has a recessive association with seborrhoeic dermatitis.

About 82% of the recessive loci did not demonstrate significant associations to related quantitative traits. There are several reasons for this. Firstly, the quantitative blood traits were extracted from the electronic health records, and the context surrounding when these tests were taken is not accounted for, even

after the trait is normalised across the cohort. Therefore, for transient derangements in quantitative measurements that are related to the binary trait, we may not be able to pick them up in the quantitative trait GWAS. Secondly, many of these binary trait associations do not have known quantitative blood-based biomarkers for us to definitively call a significant quantitative trait association as an association to a “related trait”. For example, the variant chr5:5261658:G:A has a recessive association to the ICD10 code E14 (Unspecified diabetes mellitus). It also has additive associations to eosinophil and neutrophil levels. It is possible that an underlying immunologic cause is responsible for the diabetes association, however there is too little support from this analysis alone, and we have therefore assigned this finding a score of 0. Therefore, potentially with more relaxed definitions, or further investigation into these associations, we may be able to uncover more biological insight. As an extension to this, binary traits such as psychiatric conditions (e.g. Anxiety) may not have detectable changes in known, routinely-taken, blood-based biomarkers. Lastly, it is also possible that these variants may act recessively on not just the binary trait, but on the related quantitative trait as well. Therefore, they may still be missed out in the additive GWAS of quantitative traits.

In order to quantify the extent to which these reasons contribute to our findings (or lack thereof), we assign an additional score to the binary phenotypes, called “Phenotype with Relevant Quantitative Trait Score”, grading them based on the likelihood of them having a derangement in a quantitative blood-based measurement:

- 0: Unlikely to be associated with a quantitative blood-based measurement that has been tested. Examples of the binary phenotypes in this category include psychiatric disorders such as Anxiety, and mild dermatological conditions such as Melanocytic Naevi.
- 1: Possible association to a related blood-based measurement that has been tested, such as non-specific inflammatory markers. Or possible association to acute and transient derangements that may or may not be captured in the blood tests taken for the quantitative association analyses.
- 2: Association to a related blood-based measurement is expected. This includes binary phenotypes that require a blood-based measurement as part of the diagnosis (e.g. anaemia, diabetes), as well as chronic conditions that are likely to have blood-based derangements eventually captured in some of the testing used for quantitative association analyses.

Of the 185 recessive associations, 77 (42%) are associated with binary phenotypes with a “Phenotype with Relevant Quantitative Trait Score” of 0 and are not expected to have quantitative trait associations. Next, a further 54 are associated with binary phenotypes with a “Phenotype with Relevant Quantitative Trait Score” of 1, of which 12 (22% of the 54) have significant associations to related quantitative traits (“Quantitative.GH.Score” of 1), and 2 (4% of the 54) have a “Quantitative.GH.Score” of 2. Finally, in the remaining 54 associations that are associated with binary phenotypes that are expected to have an association with a related blood-based measurement (“Phenotype with

Relevant Quantitative Trait Score" of 2), 17 (31% of the 54) indeed have significant associations to quantitative traits directly involved in the diagnosis or presentation of the binary trait ("Quantitative.GH.Score" of 2) with an additional 2 (4% of the 54) having a "Quantitative.GH.Score" of 1.

In summary, about 35%, or 19 of 54 binary phenotypes expected to be associated with quantitative traits that have been tested do indeed reflect the relevant quantitative associations. As this categorisation is slightly subjective, we think that some of the reasons stated above for why the other associations did not demonstrate significant associations to related quantitative traits still apply. Nevertheless, from this analysis, we have managed to gain insight into the possible underlying biology contributing to these recessive binary trait associations (at least 18-35% of our findings) from evaluating the additive GWAS on quantitative traits.

A systematic review of the variants associated with thalassaemia and hereditary anaemias

On 22 October 2024, for the chromosome 11 variants associated with thalassaemia and hereditary anaemias, we also searched for expression quantitative trait loci (eQTL) results in genetics.opentargets.org. Positive results were collated in Note S11.

Note S11: A compilation of literature support for the 185 recessive loci

The document is available at

https://drive.google.com/file/d/1fxM1hmQPEozUAUIChQ-q1Md8ohyU2ZBA/view?usp=sharing

Note S12: FinnGen information, ethics statement, materials and methods

FinnGen was launched in 2017 (https://www.finngen.fi/), and it is a pre-competitive collaboration between biobanks in Finland and their supporting organisations such as universities and university hospitals. There is also involvement from international partners from the pharmaceutical industry, and the Finnish biobank cooperative (FINBB). All the FinnGen partners are listed here: https://www.finngen.fi/en/partners.

Patients and control subjects in FinnGen provided informed consent for biobank research, based on the Finnish Biobank Act. Alternatively, separate research cohorts, collected prior the Finnish Biobank Act came into effect (in September 2013) and start of FinnGen (August 2017), were collected based on study-specific consents and later transferred to the Finnish biobanks after approval by Fimea (Finnish Medicines Agency), the National Supervisory Authority for Welfare and Health. Recruitment protocols followed the biobank protocols approved by Fimea. The Coordinating Ethics Committee of the Hospital District of Helsinki and Uusimaa (HUS) statement number for the FinnGen study is Nr HUS/990/2017.

The FinnGen study is approved by Finnish Institute for Health and Welfare (permit numbers: THL/2031/6.02.00/2017, THL/1101/5.05.00/2017, THL/341/6.02.00/2018, THL/2222/6.02.00/2018, THL/283/6.02.00/2019, THL/1721/5.05.00/2019 and THL/1524/5.05.00/2020), Digital and population data service agency (permit numbers: VRK43431/2017-3, VRK/6909/2018-3,

VRK/4415/2019-3), the Social Insurance Institution (permit numbers: KELA 58/522/2017, KELA 131/522/2018, KELA 70/522/2019, KELA 98/522/2019, KELA 134/522/2019, KELA 138/522/2019, KELA 2/522/2020, KELA 16/522/2020), Findata permit numbers THL/2364/14.02/2020, THL/4055/14.06.00/2020, THL/3433/14.06.00/2020, THL/4432/14.06/2020, THL/5189/14.06/2020, THL/5894/14.06.00/2020, THL/6619/14.06.00/2020, THL/209/14.06.00/2021, THL/688/14.06.00/2021, THL/1284/14.06.00/2021, THL/1965/14.06.00/2021, THL/5546/14.02.00/2020, THL/2658/14.06.00/2021, THL/4235/14.06.00/2021, Statistics Finland (permit numbers: TK-53-1041-17 and TK/143/07.03.00/2020 (earlier TK-53-90-20) TK/1735/07.03.00/2021, TK/3112/07.03.00/2021) and Finnish Registry for Kidney Diseases permission/extract from the meeting minutes on 4th July 2019.

The Biobank Access Decisions for FinnGen samples and data utilized in FinnGen Data Freeze 10 include: THL Biobank BB2017_55, BB2017_111, BB2018_19, BB_2018_34, BB_2018_67, BB2018_71, BB2019_7, BB2019_8, BB2019_26, BB2020_1, BB2021_65, Finnish Red Cross Blood Service Biobank 7.12.2017, Helsinki Biobank HUS/359/2017, HUS/248/2020, HUS/150/2022 § 12, §13, §14, §15, §16, §17, §18, and §23, Auria Biobank AB17-5154 and amendment #1 (August 17 2020) and amendments BB_2021-0140, BB_2021-0156 (August 26 2021, Feb 2 2022), BB_2021-0169, BB_2021-0179, BB_2021-0161, AB20-5926 and amendment #1 (April 23 2020)and it´s modification (Sep 22 2021), Biobank Borealis of Northern Finland_2017_1013, 2021_5010, 2021_5018, 2021_5015, 2021_5023, 2021_5017, 2022_6001, Biobank of Eastern Finland 1186/2018 and

amendment 22 § /2020, 53§/2021, 13§/2022, 14§/2022, 15§/2022, Finnish Clinical Biobank Tampere MH0004 and amendments (21.02.2020 & 06.10.2020), §8/2021, §9/2022, §10/2022, §12/2022, §20/2022, §21/2022, §22/2022, §23/2022, Central Finland Biobank 1-2017, and Terveystalo Biobank STB 2018001 and amendment 25th Aug 2020, Finnish Hematological Registry and Clinical Biobank decision 18th June 2021, Arctic biobank P0844: ARC_2021_1001.

Supplemental figures

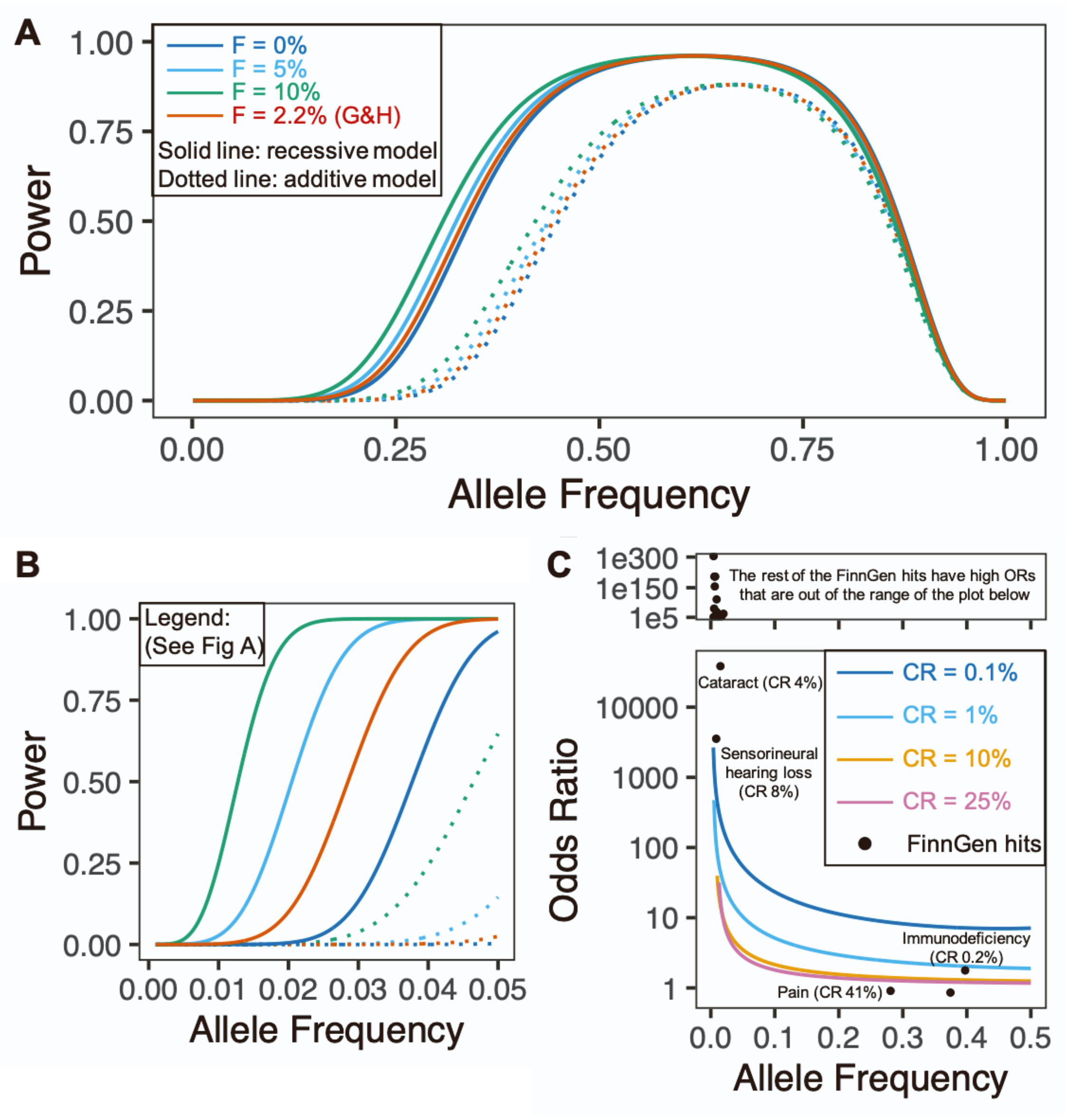

Figure S1

Simulations to evaluate power to detect a recessive effect in G&H when using a recessive versus additive model. A: Calculating power to detect an association at $p<5x10^{-8}$ for a range of allele frequencies and values for average inbreeding coefficient (F). Sample size = 44,000, OR = 2, CR = 1%. B: Calculating power for a range of rarer allele frequencies and values for F. Parameters as per A, except OR = 5. C: Calculating the minimum odds ratio

G&H would be powered to detect at 80% power, and F = 2.2%. ”CR” : Case rate. Recessive hits reported in FinnGen [3] are indicated on the plot, showing their OR and AF estimated in FinnGen.

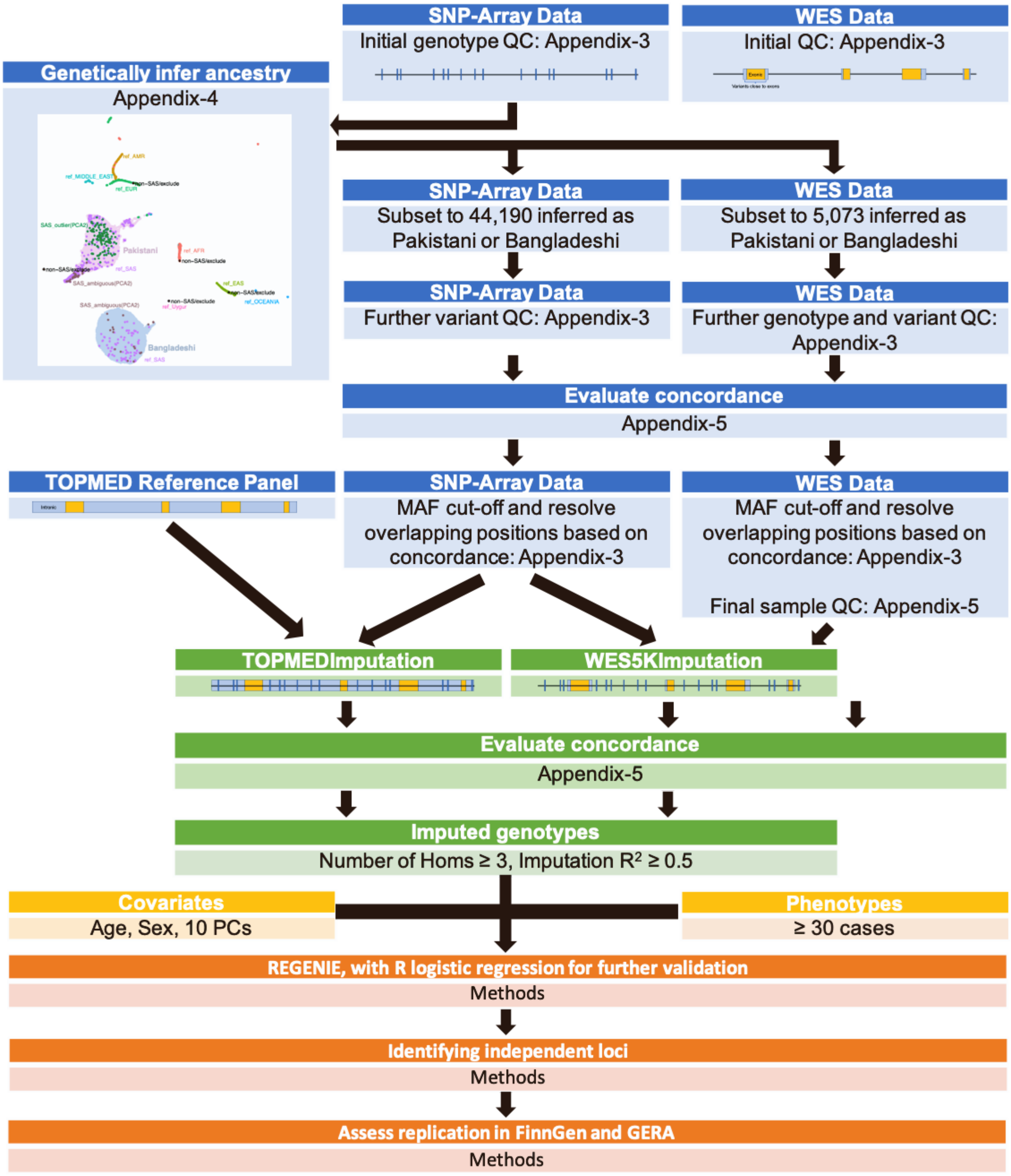

Figure S2

A flowchart to visualise the methods of this project. This is intended to serve as a directory to relevant sections.

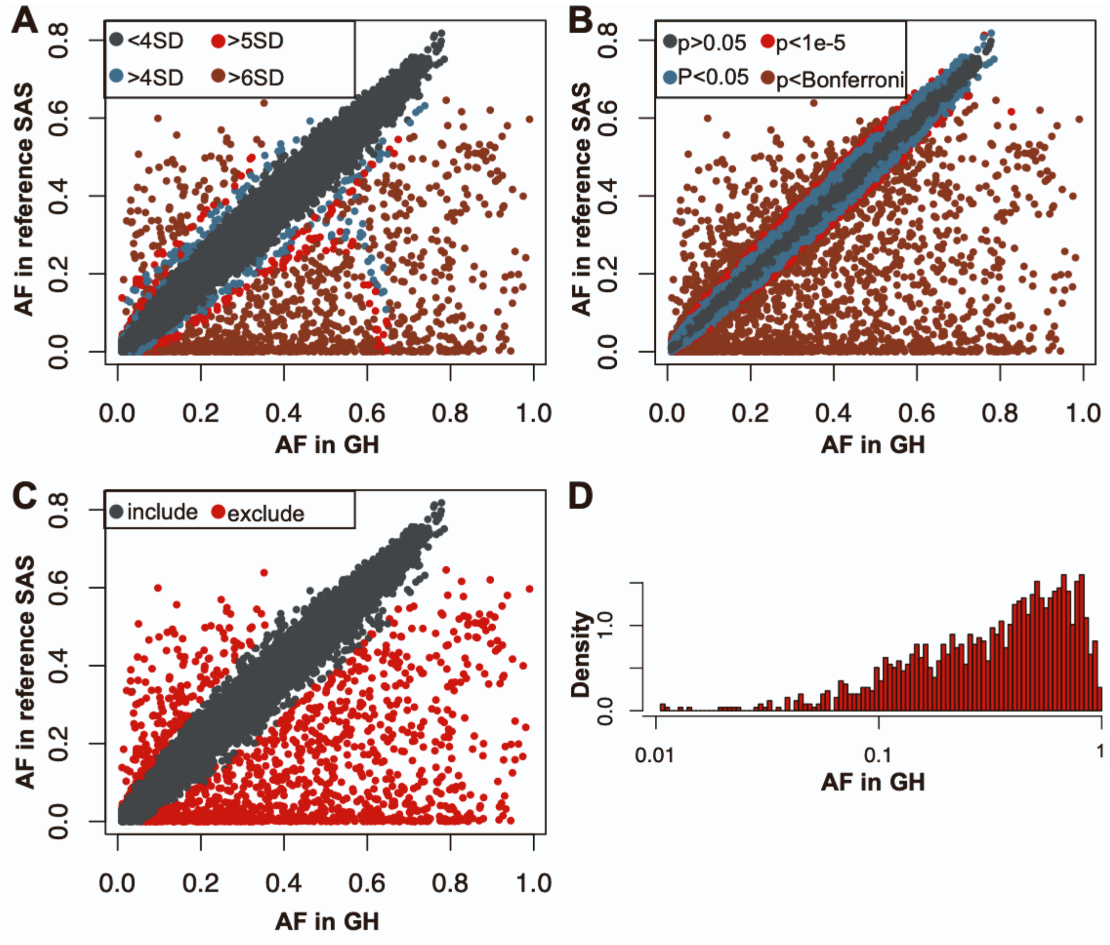

Figure S3

Excluding variants with significantly different AF in Genes and Health (GH) and 676 South Asian samples from 1000 Genomes and HGDP (reference SAS). Plotting the AFs in both cohorts for each variant, colour-coding represents in A: the different standard deviation (SD) thresholds applied to the mean of the residuals in 0.01 frequency bins, in B: the different p-value thresholds for Fisher's exact tests on the genotypes, and in C: the variants that were included and excluded from the merged panel of variants. D: The AF distribution of the variants excluded.

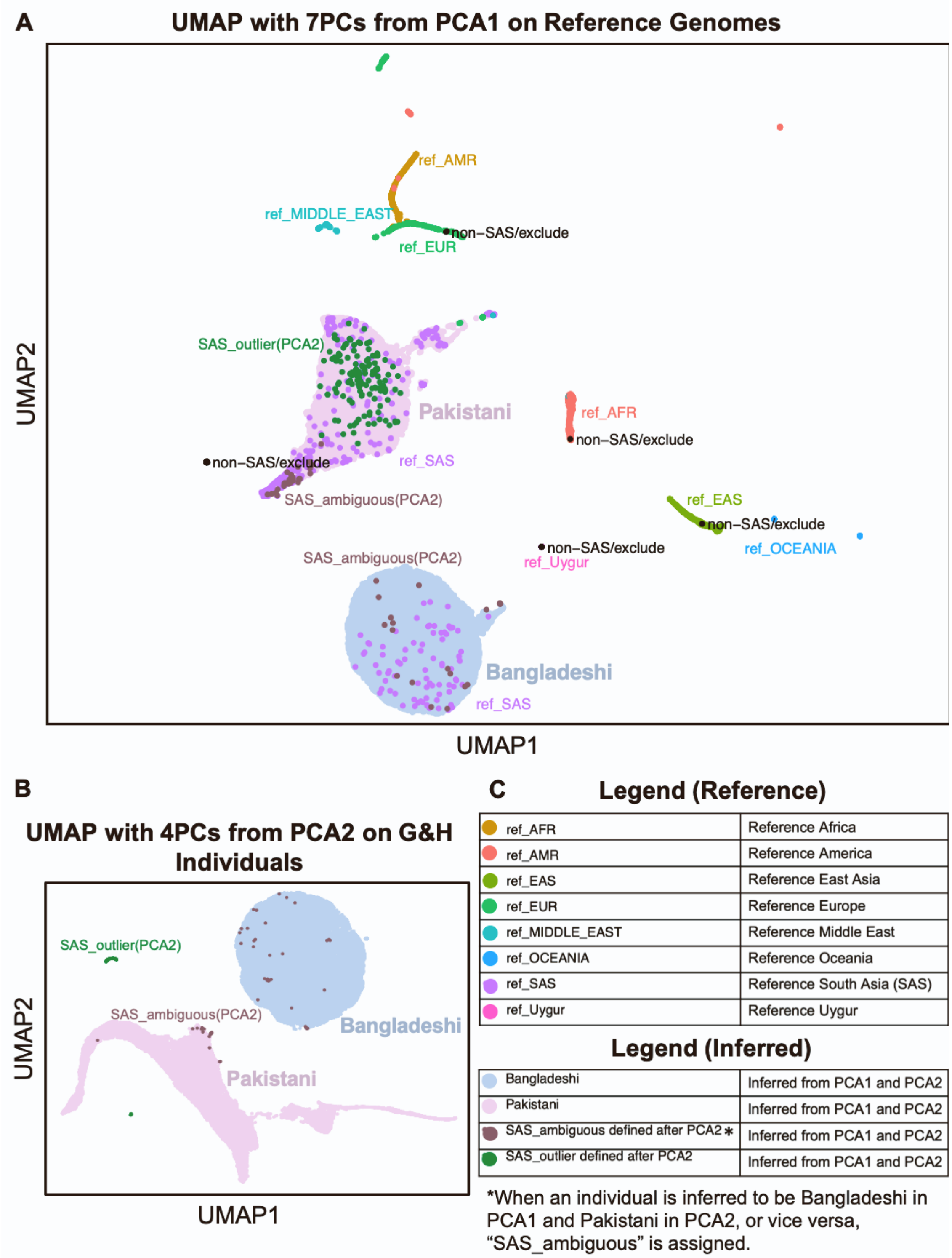

Figure S4

Genetic inference of ancestry using the G&H SNP-array data. A: UMAP with 7 PCs from the PCA of reference individuals (PCA1), with G&H individuals

projected into the PC space. B: UMAP with 4 PCs from the PCA of unrelated G&H individuals (PCA2), with related G&H individuals projected into the PC space. C: Legends for both UMAPs.

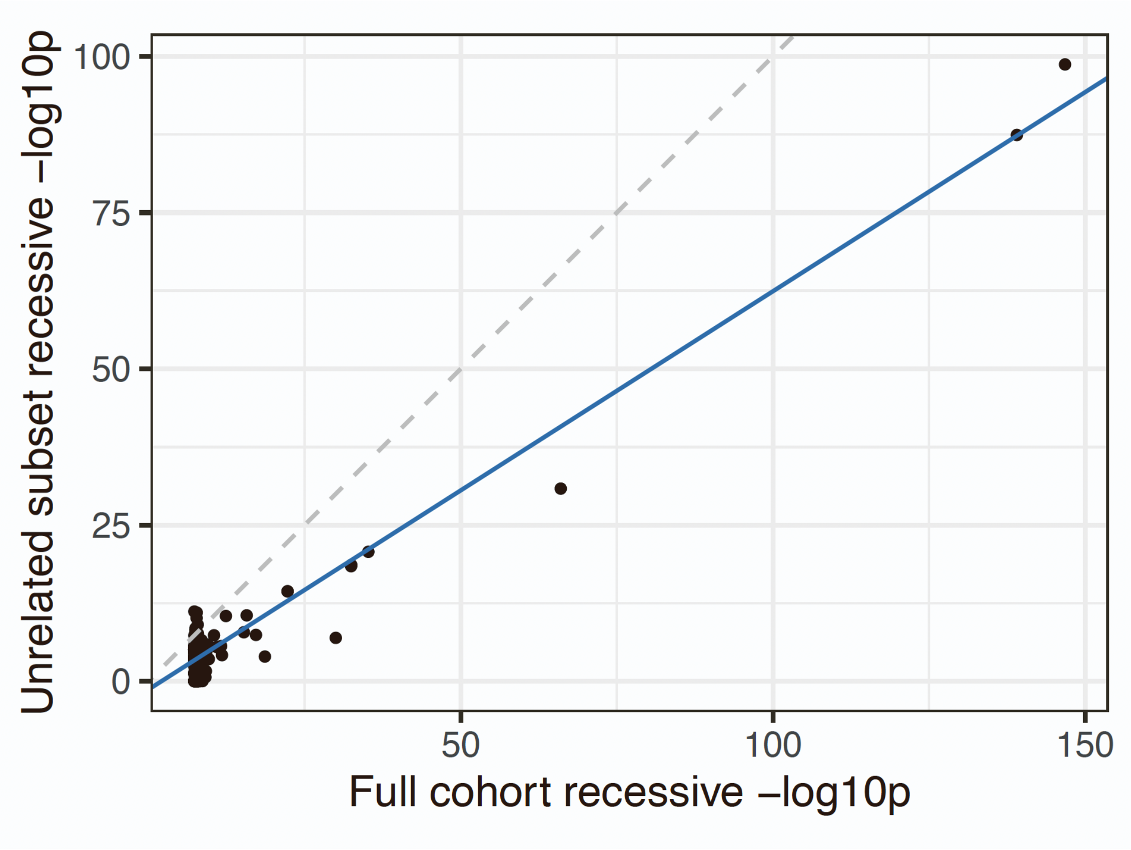

Figure S5

P-values (in -log10p) of the 185 lead variants from the recessive analysis performed on the unrelated subset (y-axis) compared to the recessive analysis performed on the full cohort (x-axis). The blue solid line represents the line of best fit, while the grey dashed line represents the x=y line.

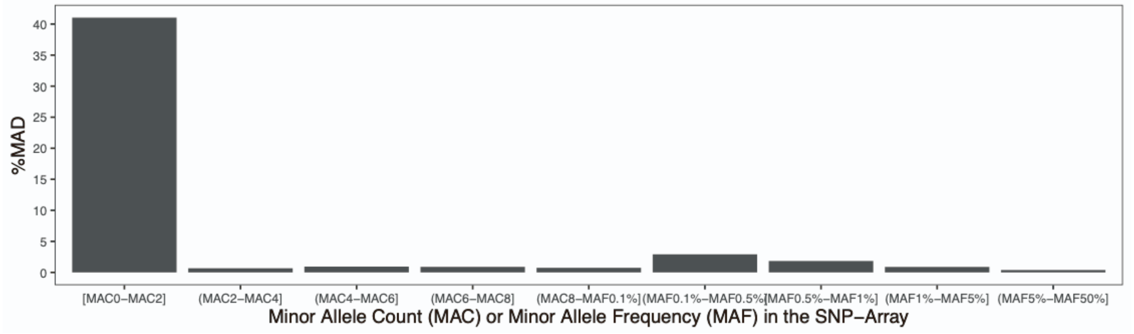

Figure S6

The Minor Allele Discordance (MAD) between the SNP-Array and the WES at overlapping variants, stratified by the frequency on the array. Array filters: After initial genotype QC + Call rate ≥99% + HWE pval ≥10^{-6}. WES filters: Call rate ≥70% after GQ ≥20 + binomAD ≥10^{-2} + DP>7. Here, the array genotypes were treated as “truth”. As N~5,000, a MAC of 2 converts to ~$2x10^{-4}$ (or ~0.02%) in MAF.

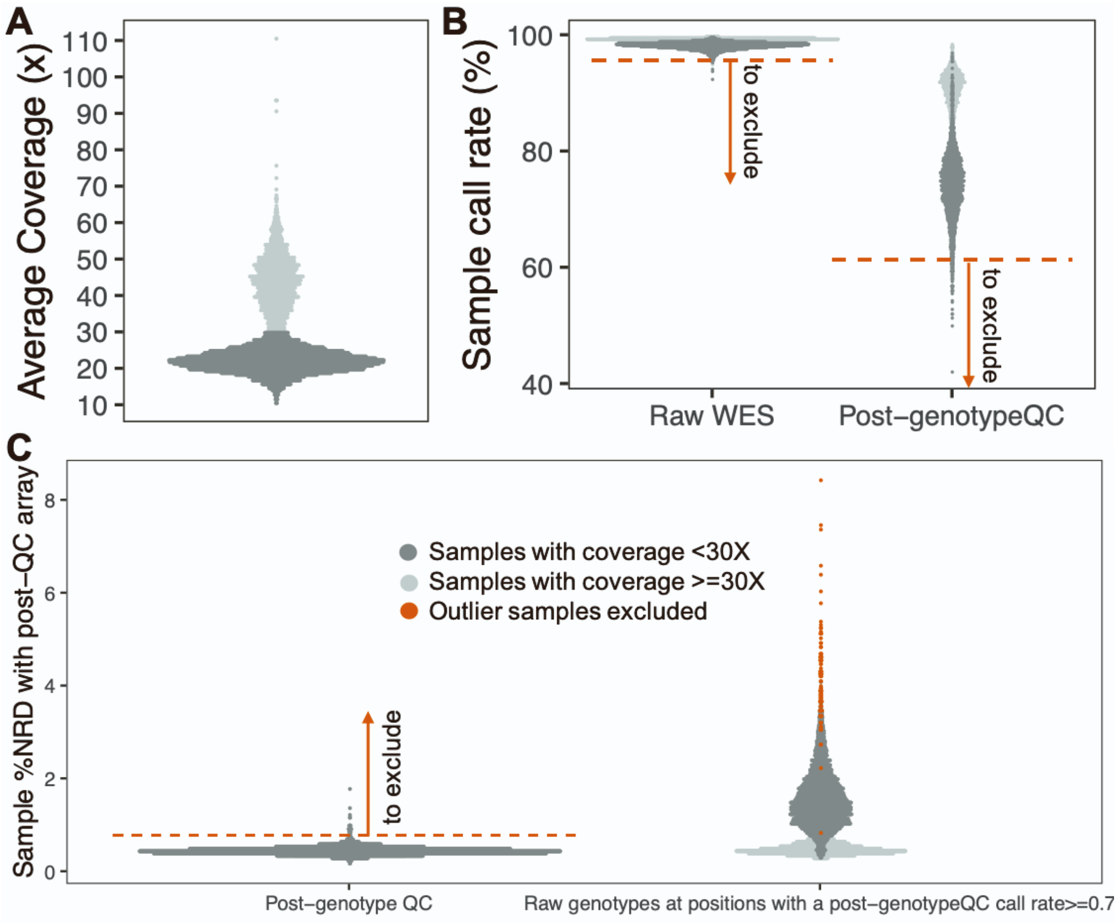

Figure S7

Per-sample coverage, call rate, and non-reference discordance (NRD) before and after QC of WES data. A: Distribution of average on-target coverage across samples. The bimodality observed is because different batches of samples were sequenced to either ~20X or ~40X. B: The distribution of WES sample call rates pre-genotype QC (left) and post-genotype QC (right). C: The distribution of NRDs across samples in the post-genotype QC WES. In B and C, the bimodality is again likely due to the different sequencing coverage.

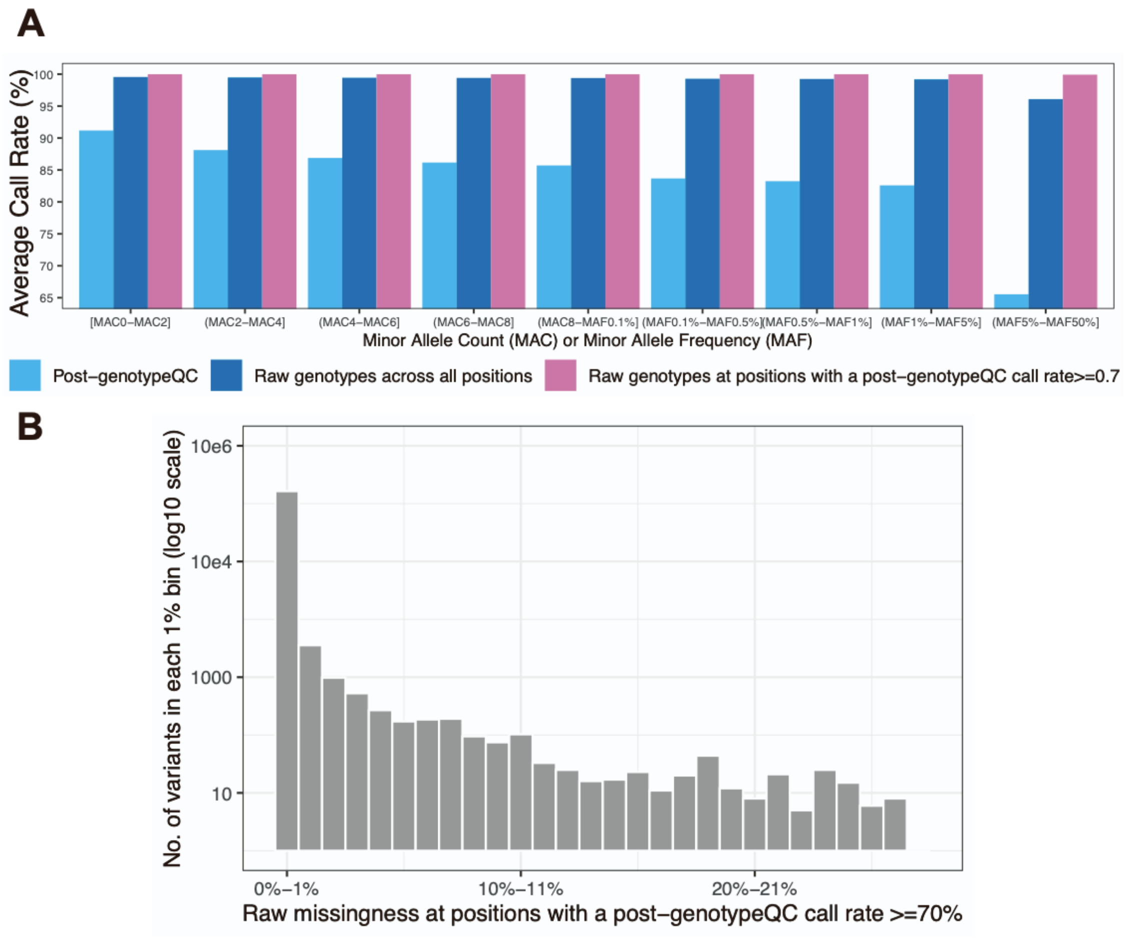

Figure S8

Distribution of call rates pre and post QC in the G&H WES data. A: Average call rate stratified across MAF bins. As the missingness is low in the raw WES, when filtering to positions with a post-genotypeQC call rate ≥70%, the call rate of the raw genotypes is >99% across the frequency spectrum. As N~5,000, a MAC of 2 converts to ~2×10^{-4} (or ~0.02%) in MAF. B: Distribution of per-variant raw missingness at positions with a post-genotype QC call rate ≥70%. The y-axis has been transformed to a log 10 scale as an overwhelmingly large number of variants have a raw missingness of 0-1%.

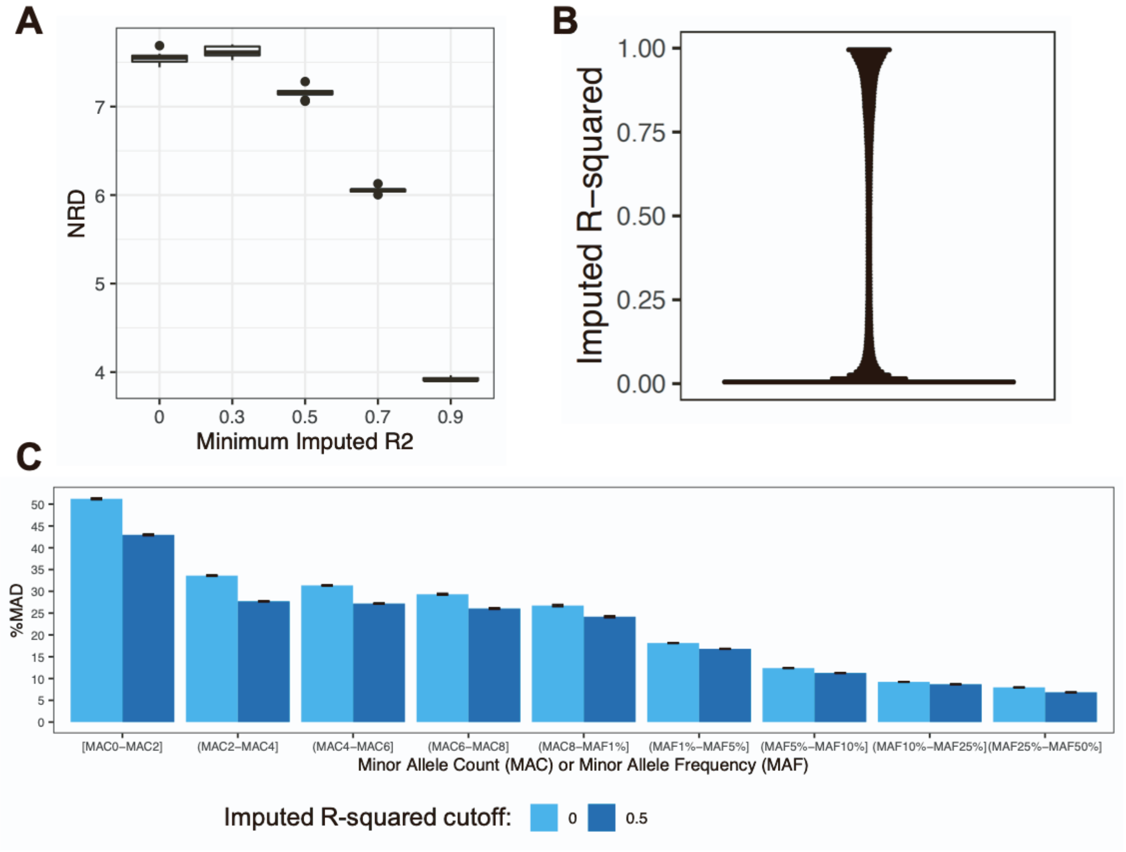

Figure S9

Results of the leave-10%-out trials. A: The distribution of NRD(%) across 10 trials when different minimum imputed r^2 cutoffs are applied. B: The distribution of imputed r^2 scores for a representative trial. C: The MAD before and after applying an imputed r^2 ≥0.5 cut-off, stratified by MAF. (Error bars are the standard errors in %MAD across the 10 trials.) As the sample size is 10% each time, i.e. N~500, the MAC of 2 converts to ~$2x10^{-3}$ (or ~0.2%) in MAF.

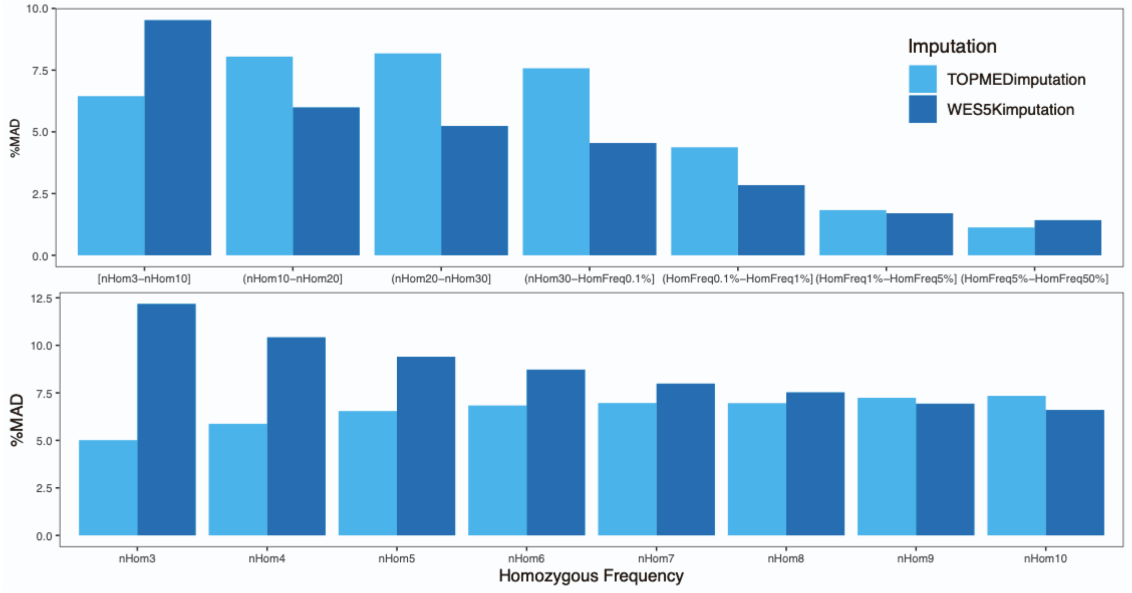

Figure S10

Minor allele discordance (MAD) of the TOPMEDimputation compared to the WES, and the mean MAD across ten leave-10%-out trials compared to the WES (represented as the WES5Kimputation for simplicity), stratified by the number or frequency of homozygous genotypes in the WES data. The variant counts contributing to each bin is tabulated below.

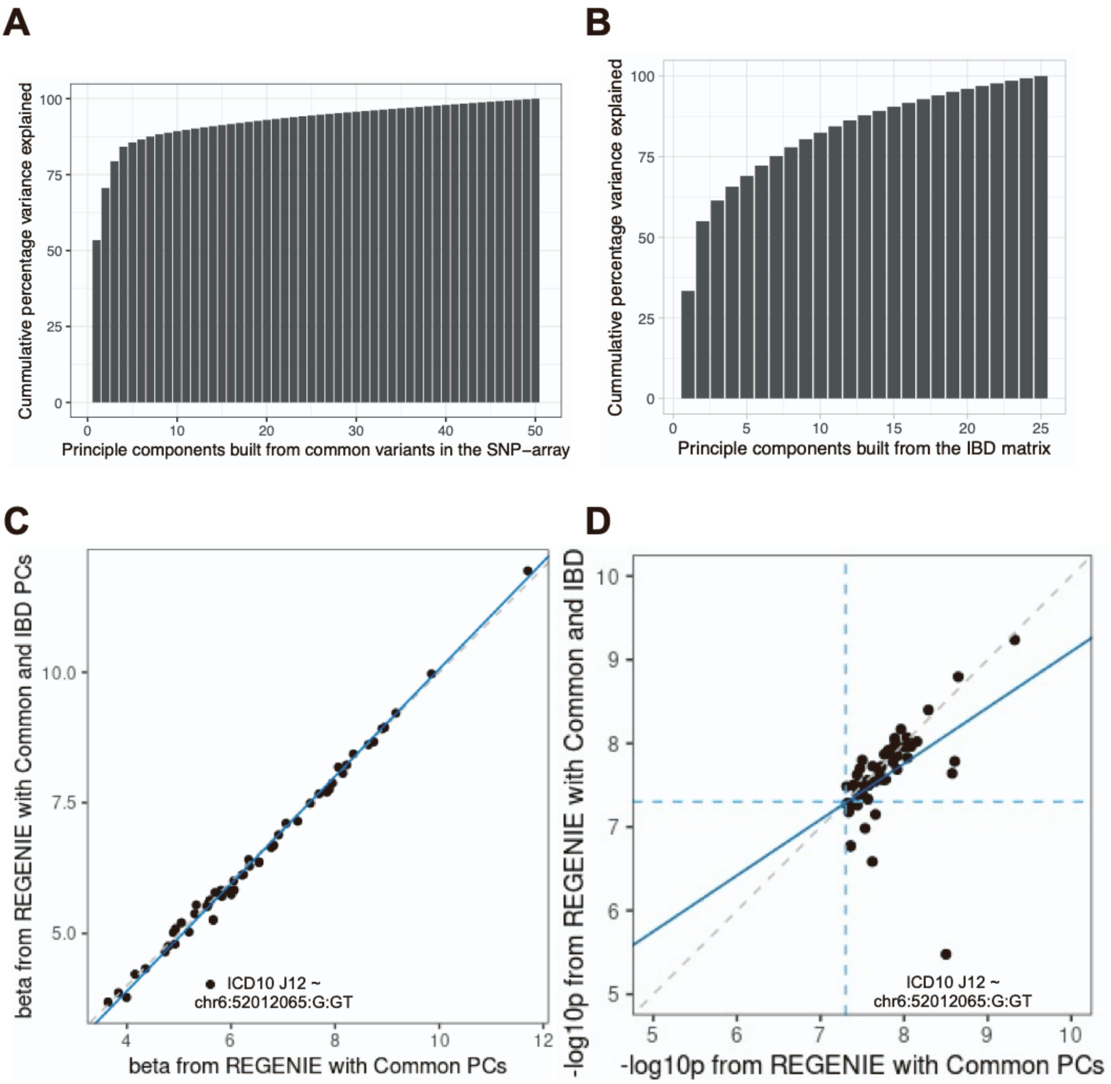

Figure S11

Principal component analyses of population structure. A: The cumulative percentage variance explained across the 50PCs generated from common variants in the SNP-array, with the variance explained by all 50PCs scaled to 100%. B: The cumulative percentage variance explained across the 25PCs generated from the IBD matrix, with the variance explained by all 25PCs scaled to 100%. C&D: For the 56 recessive loci associated with rare (AF 1% and lower) variants, the association analysis was repeated controlling for 10 common variant PCs and 15 IBD PCs, and the betas (C) p-values (D) were

compared. The blue solid line represents the line of best fit, the grey dashed line represents the x=y line, the blue dashed line represents the $p=5x10^{-8}$ cutoff. The outlier finding has been labelled.

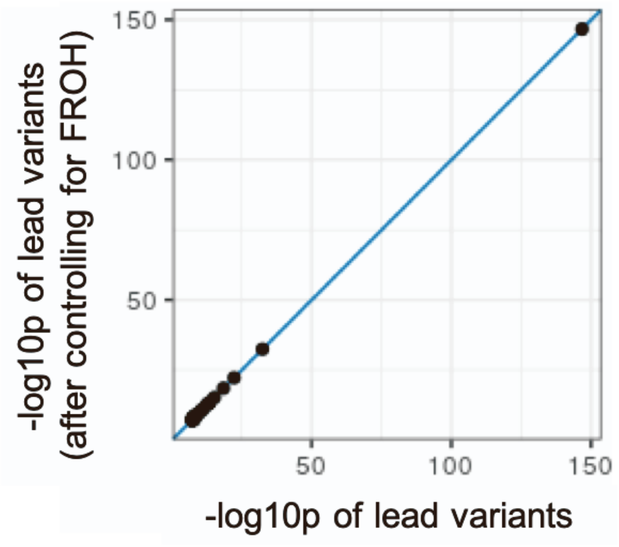

Figure S12

Scatter plot of the WES5Kimputation lead variants p-values (-log10p) compared to their p-values when the recessive test is rerun to control for F_{ROH}.

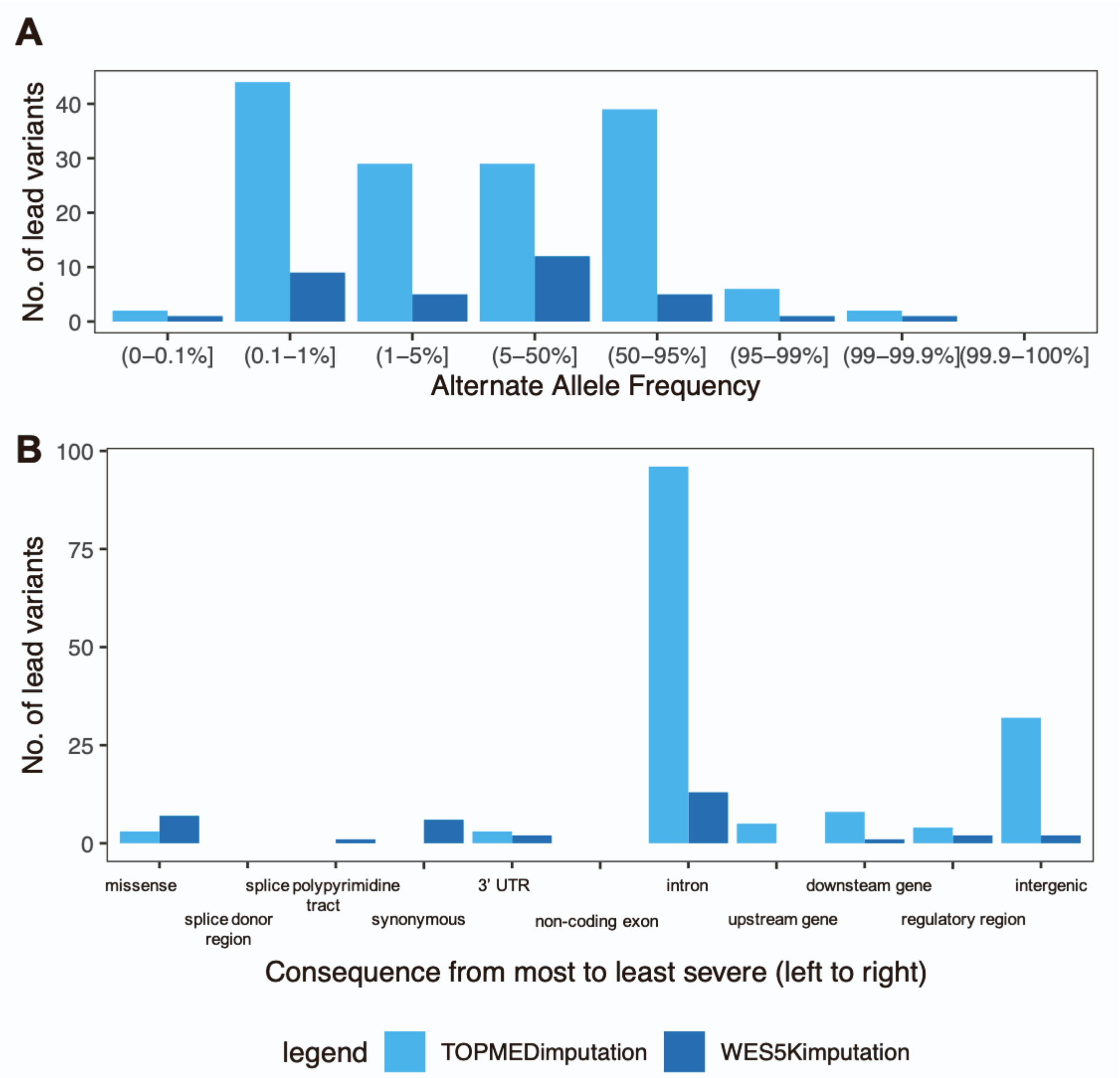

Figure S13

Characteristics of lead SNPs in the recessive findings. A: Distribution of the allele frequencies of the lead SNPs. B: Distribution of the variant consequences of the lead SNPs.

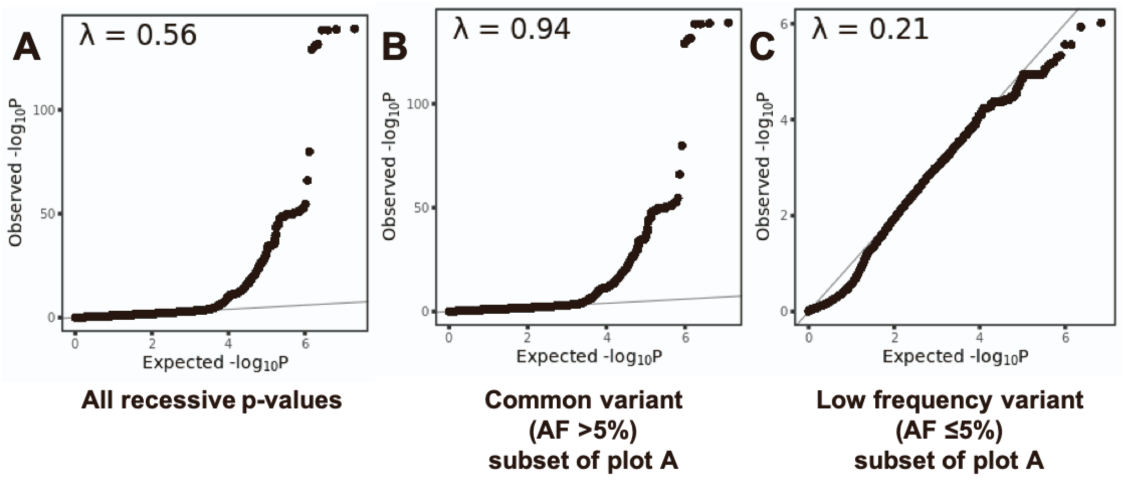

Figure S14

Quantile-quantile (QQ) plots (B-D) for the phenotypes that have Bonferroni-significant findings. In A, all the recessive p-values in the D58[Other hereditary haemolytic anaemias] run have been plotted. We then split the QQ-plot into common variants (AF > 5%, B) and low frequency variants (C).

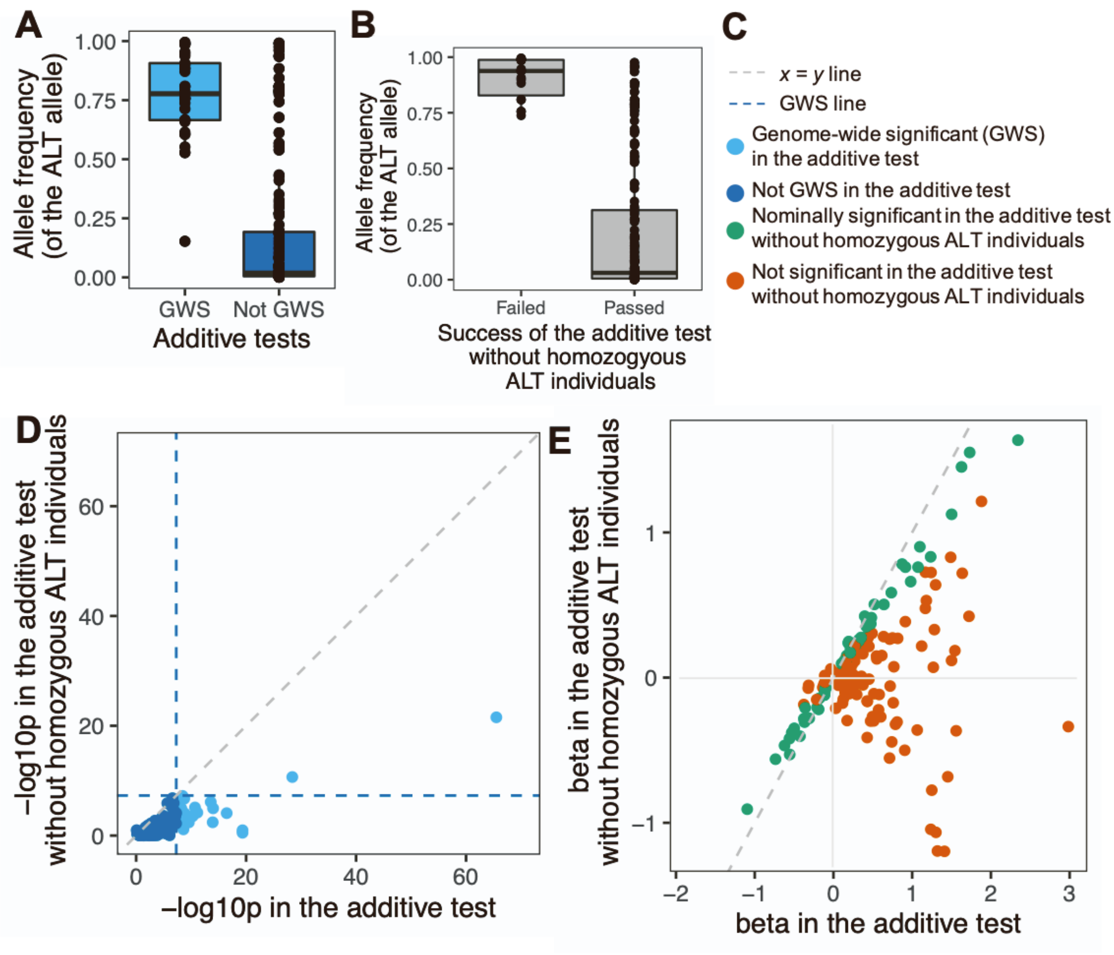

Figure S15

Exploring the recessive lead variants with different models of testing in REGENIE. A: AF distribution of recessive hits that were GWS and not GWS in additive tests. B: AF distribution of recessive hits that could and could not run under the additive model without homozygous individuals. C: legend for the figure. D, E: P-values (-log10p, D) and betas (E) in the additive tests compared to the additive tests without homozygotes.

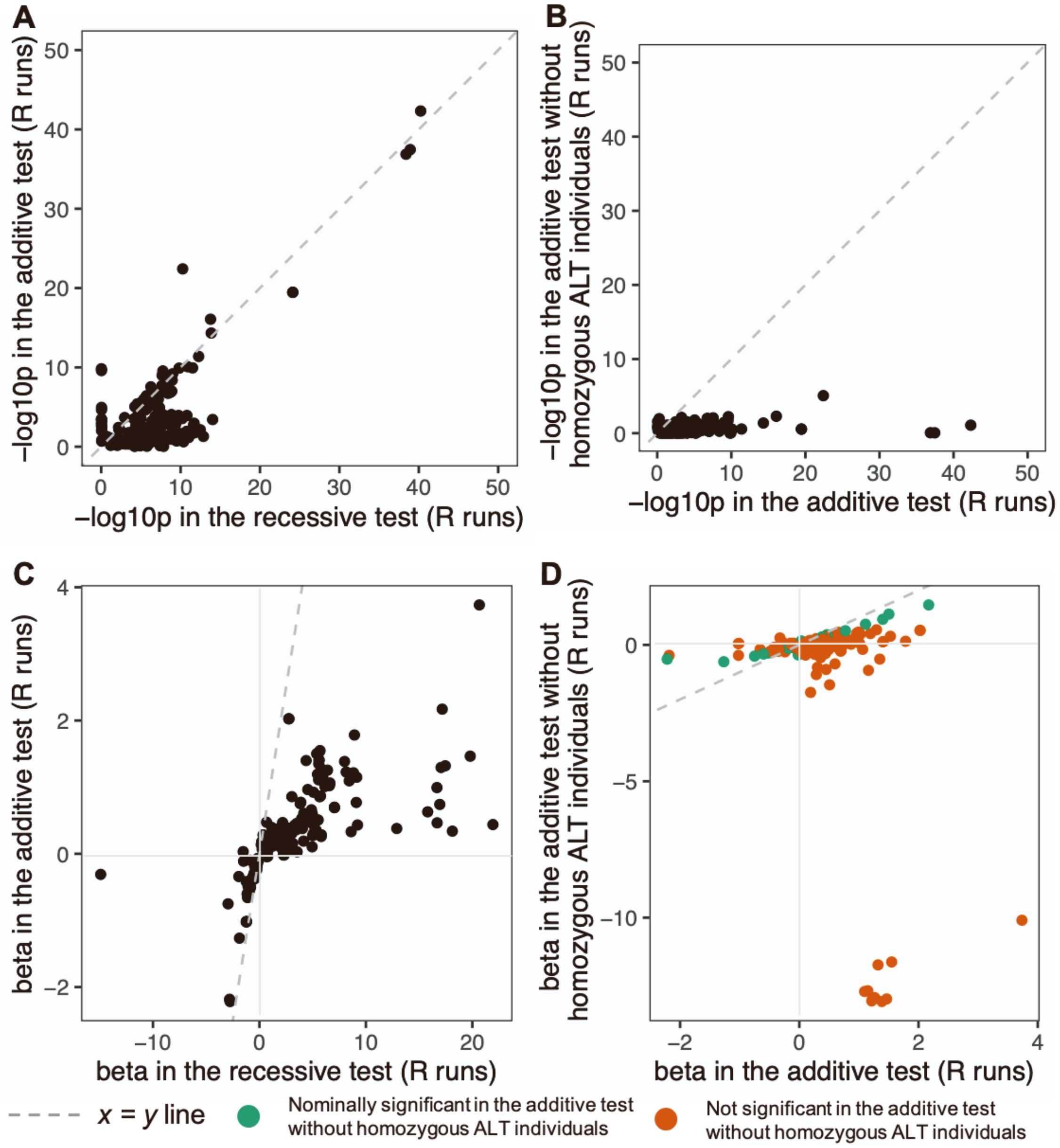

Figure S16

Exploring the recessive lead variants with different models of testing in R.

A,C: P-values (-log10p, A) and betas (C) in the recessive tests compared to the additive tests in R. B,D: P-values (-log10p, B) and betas (D) in the additive tests compared to the additive tests without homozygotes in R.

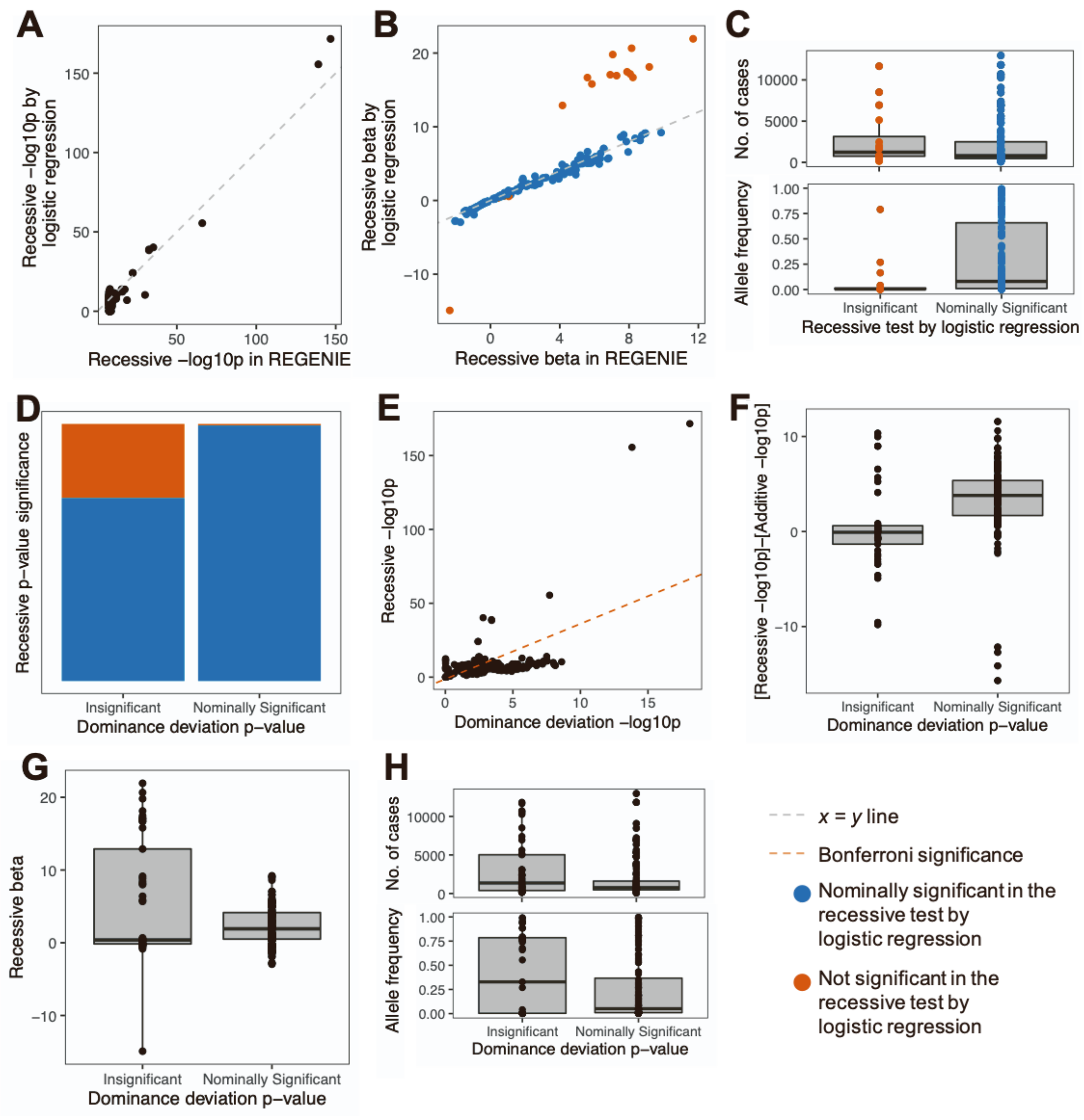

Figure S17

Logistic regression testing in R for the recessive findings. A,B: P-values (-log10p, A) and betas (B) in REGENIE compared to logistic regression testing. C: Distributions of AF and case counts between nominally significant and insignificant (p-value > 0.05) tests in the recessive logistic regression. D-F: Distribution of recessive logistic regression p-values(D-E), the differences between the recessive -log10p and the additive -log10p (F), betas (G), and AF and case counts (H) across the dominance deviation tests.

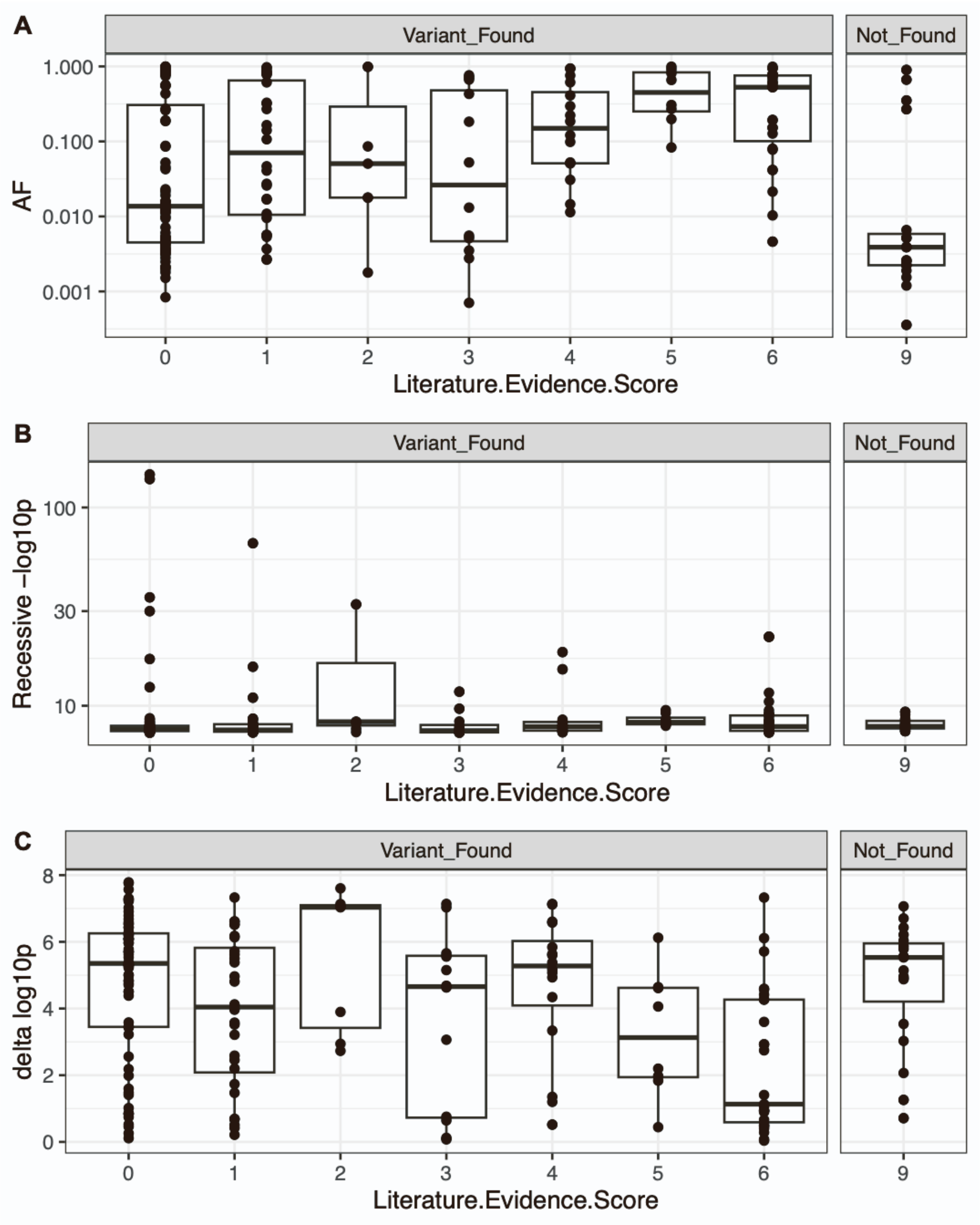

Figure S18

Lead variant characteristics plotted across Literature.Evidence.Scores. A: Allele frequencies (AFs) have been plotted. B: Recessive -log10p values have been plotted. C: Delta log10p (Subtracting the additive -log10p from the recessive -log10p) have been plotted.

Supplemental tables

	SNP-Array Data	WES Data
**Initial QC**	**Initial genotype QC**<br>44,396 individuals<br>Variant QC in GenomeStudio<br>Sample QC (exclude samples with low call rate or failed sex checks)	5,236 individuals after light sample QC (exclude samples with low coverage or sex discrepancies)
**Genetically infer ancestry**	44,190 individuals assigned as Pakistani or Bangladeshi	5,073 individuals with array data and assigned as Pakistani or Bangladeshi
**Genotype QC**		Set genotype as missing when: GQ <20, binomAD <10^{-2}, DP ≤7
**Variant QC**	534,806 autosomal SNPs	2,245,547 autosomal SNPs and indels, after excluding:<br>For SNPs: "QD < 2.0 || FS > 30 || MQ < 40.0 || MQRankSum < -12.5 || ReadPosRankSum < -8.0"<br>For indels: "QD < 2.0 || FS > 30 || ReadPosRankSum < -20.0"
	534,531 variants with a call rate ^{3}99%	1,645,161 variants with a post-genotype QC call rate ^{3}70%
	533,362 variants with HWE p>=10^{-6} in the Bangladeshi subgroup	
	533,166 biallelic variants	
	Evaluate Concordance	
**MAF cut-offs**	Keep 469,678 variants with MAF >0.1%	Remove singletons
**Resolve Overlapping Positions**	Exclude 25 common palindromic variants with MAF >40%	Keep, as no problem of allele-switching
	Exclude 515 variants with overlapping positions but unmatched alleles	Keep, as some are indels that have been genotyped incorrectly on the array
	Keep 38,248 variants with matched alleles and MAF >0.1%	Exclude, as the array has a higher overall call rate<br>(Note: rare variants with MAF ≤0.1% that overlapped with the array are retained)
**Final Sample QC**		4,982 individuals after excluding: Pre-genotype QC call rate <4SD from mean, Post-genotype QC call rate <2SD from mean, Post-genotype QC NRD >4SD from mean

Table S1

QC steps for the SNP-Array and WES Data

	Raw Genotypes (GTs)	GQ >=20	Call rate >=70% after: GQ >=20 binomAD >=10^{-2} DP>7	With raw GT at sites with call rate >=70% after: GQ >=20 binomAD >=10^{-2} DP>7
**After initial genotype QC**	5.018% (46,778 SNPs)			
**Call rate >=99% HWE pval >=10-6**	5.000% (46,656 SNPs)	0.500 % (46,401 SNPs)	0.398% (38,249 SNPs)	1.258 % (38,249 SNPs)

Table S2

Overall non-reference discordance (NRD) between the array and WES data with different QC filters applied to the SNP-array data and the WES data. Row names describe stages of SNP-array filtering, column names describe stages of WES filtering. “Initial genotype QC” is described in the first row of Table S1.

	n variants (TOPMEDimputation)	n variants (WES5Kimputation)	WES5Kimputation / TOPMEDimputation
**nHom 3**	10,806	26,001	2.41
**nHom 4**	10,855	19,588	1.80
**nHom 5**	9,771	15,532	1.59
**nHom 6**	8,725	12,615	1.45
**nHom 7**	7,701	10,546	1.37
**nHom 8**	6,875	9,044	1.32
**nHom 9**	6,233	7,864	1.26
**nHom 10**	5,519	6,774	1.23
**(nHom 10 – nHom 20]**	35,924	41,259	1.15
**(nHom 20 – nHom 30]**	18,717	20,156	1.08
**(nHom 30 – HomFreq 0.1%]**	17,065	17,907	1.05
**(HomFreq 0.1% - HomFreq 1%]**	109,350	111,916	1.02
**(HomFreq 1% - HomFreq 5%]**	106,049	109,146	1.03
**(HomFreq 5% - HomFreq 10%]**	151,338	173,978	1.15

Table S3

Number of imputed variants (that can be compared to WES positions for concordance analyses) binned by the number or frequency of homozygous genotypes in the WES data.

Phenotypes	n phenotypes	n individuals	n variants (WES5KImputation)	n variants (TOPMEDImputation)
Custom phenotypes (In both sexes)	199	44,186	605,263	10,045,406
Custom phenotypes (Female only)	15	24,387	553,716	9,215,675
Custom phenotypes (Male only)	5	19,799	529,480	8,855,245
ICD10 codes (In both sexes)	565	42,027	599,758	9,948,708
ICD10 codes (Female only)	99	23,710	550,988	9,171,960
ICD10 codes (Male only)	15	18,317	522,117	8,741,181

Table S4

The number of phenotypes, individuals, and variants being tested. The number of variants tested varied with the number of individuals tested, as different subsets of individuals would result in different numbers of variants having at least three homozygotes.

	coding	non-coding
All tested	91,930	9,953,476
Recessive variants	40	1,090

Table S6

Table of the number of coding and non-coding variants tested in the TOPMEDImputation, compared to the number of coding and non-coding variants among the TOPMEDImputation variants with a genome-wide-significant recessive p-value that was smaller than their additive p-value, before defining loci and filtering by LD.

Genotype	Additive	Recessive	Dominance deviation
RR	0	0	0
RA	1	0	1
AA	2	1	0

Table S7

Genotype encodings for the additive, recessive and dominance deviation tests performed as logistic regression tests in R. “RR” refers to the homozygous wild type, “RA” the heterozygous genotype, and “AA” the homozygous alternate genotype.

Gene	Lead variant	AF	CSQ	Phenotype	OR	p-value	DD p-value
F_{ROH}-Associated Phenotypes:							
**A09[Other gastroenteritis and colitis of infectious and unspecified origin]**							
.	2:171680912:G:A	0.3	intergenic	A09[Other gastroenteritis and colitis of infectious and unspecified origin]	0.6	2.7E-08	6.0E-02
**E11[Type 2 diabetes mellitus]**							
.	6:104459545:G:A	0.01	regulatory region	E11[Type 2 diabetes mellitus]	38	3.6E-08	7.2E-06
Y_RNA	10:92706738:G:C	0.7	downstream gene	GNH0242 Type 2 Diabetes narrow	0.8	8.8E-09	1.0E-01
Y_RNA	10:92706682:A:G	0.8	downstream gene	Type 2 Diabetes	0.8	4.3E-09	5.6E-02
ADAMTS16	5:5261658:G:A	0.01	intron	E14[Unspecified diabetes mellitus]	296	5.1E-09	6.9E-06
UBE2E2	3:23530638:C:A	0.001	intron	E14[Unspecified diabetes mellitus]	2664	4.6E-08	9.6E-01
SNX5	20:17949555:G:T	0.01	intron	GNH0244 Unspecified or Rare Diabetes narrow	63	1.4E-08	3.5E-03
**E78[Disorders of lipoprotein metabolism and other lipidaemias]**							
APOA5	11:116796367:A:G	0.9	upstream gene	E78[Disorders of lipoprotein metabolism and other lipidaemias]	0.8	3.3E-11	7.1E-03
**F41[Other anxiety disorders]**							
.	2:236834949:C:T	0.01	regulatory region	F40[Phobic anxiety disorders]	138	2.7E-08	2.6E-03
.	7:131782270:G:A	0.08	intergenic	Anxiety and phobia	1.8	3.5E-08	4.8E-03
.	7:131780486:G:A	0.08	intergenic	F41[Other anxiety disorders]	1.9	7.7E-10	2.7E-03
.	7:131773247:A:G	0.1	regulatory region	F41[Other anxiety disorders]	1.8	1.8E-08	7.7E-05
**H61[Other disorders of external ear]**							
ESRRG	1:216911690:AG:A	0.01	intron	H60[Otitis externa]	54	2.5E-09	4.7E-04
**J11[Influenza, virus not identified]**							
PKHD1	6:52012065:G:GT	0.004	intron	J12[Viral pneumonia, not elsewhere classified]	271	3.1E-09	6.6E-06
PKHD1	6:52012065:G:GT	0.004	intron	B97[Viral agents as the cause of diseases classified to other chapters]	180	2.7E-09	1.9E-05
.	6:51529735:A:G	0.002	intergenic	B97[Viral agents as the cause of diseases classified to other chapters]	430	2.4E-08	8.0E-04
**J34[Other disorders of nose and nasal sinuses]**							
ADAMTS9-AS2	3:64802433:A:G	0.03	intron	J34[Other disorders of nose and nasal sinuses]	8	1.5E-08	1.8E-07
.	7:48916122:T:C	0.02	intergenic	J34[Other disorders of nose and nasal sinuses]	13	5.0E-09	1.5E-03

.	5:91760313:C:G	0.01	intron	J34[Other disorders of nose and nasal sinuses]	11	2.4E-08	1.5E-03
**L30[Other dermatitis]**							
PTPRN2	7:157970755:T:G	0.005	intron	L20[Atopic dermatitis]	64	1.1E-08	8.6E-01
NKAIN1	1:31215264:T:C	0.9	intron	L21[Seborrhoeic dermatitis]	1.3	6.2E-09	8.4E-01
BCAR3	1:93847011:TCGGGCGCGGCGG:*	0.4	intron	Dermatitis (atopic, contact, other, unspecified)	1.2	1.6E-08	6.3E-08

Table S10

Single recessive associations involving phenotypes found to be significantly associated with genome-wide homozygosity in Malawsky et al. (2023) [22].

Supplemental acknowledgements

We thank the Human Genetics Informatics team at the Wellcome Sanger Institute for support with variant annotations.

This research was funded in part by Wellcome (grant no. 220540/Z/20/A, “Wellcome Sanger Institute Quinquennial Review 2021–2026”). For the purpose of open access, the authors have applied a CC-BY public copyright licence to any author accepted manuscript version arising from this submission.

Genes & Health is/has recently been core-funded by Wellcome (WT102627, WT210561), the Medical Research Council (UK) (M009017, MR/X009777/1, MR/X009920/1), Higher Education Funding Council for England Catalyst, Barts Charity (845/1796), Health Data Research UK (for London substantive site), and research delivery support from the NHS National Institute for Health Research Clinical Research Network (North Thames). Genes & Health is/has recently been funded by Alnylam Pharmaceuticals, Genomics PLC; and a Life Sciences Industry Consortium of Astra Zeneca PLC, Bristol-Myers Squibb Company, GlaxoSmithKline Research and Development Limited, Maze Therapeutics Inc, Merck Sharp & Dohme LLC, Novo Nordisk A/S, Pfizer Inc, Takeda Development Centre Americas Inc.

T. H. Heng is supported by the Agency for Science, Technology, and Research (A*STAR) National Science Scholarship.

We thank Social Action for Health, Centre of The Cell, members of our Community Advisory Group, and staff who have recruited and collected data

from volunteers. We thank the NIHR National Biosample Centre (UK Biocentre), the Social Genetic & Developmental Psychiatry Centre (King's College London), Wellcome Sanger Institute, and Broad Institute for sample processing, genotyping, sequencing and variant annotation.

This work uses data provided by patients and collected by the NHS as part of their care and support.

We thank: Barts Health NHS Trust, NHS Clinical Commissioning Groups (City and Hackney, Waltham Forest, Tower Hamlets, Newham, Redbridge, Havering, Barking and Dagenham), East London NHS Foundation Trust, Bradford Teaching Hospitals NHS Foundation Trust, Public Health England (especially David Wyllie), Discovery Data Service/Endeavour Health Charitable Trust (especially David Stables), Voror Health Technologies Ltd (especially Sophie Don), NHS England (for what was NHS Digital) - for GDPR-compliant data sharing backed by individual written informed consent.

We want to acknowledge the participants and investigators of the FinnGen study. The FinnGen project is funded by two grants from Business Finland (HUS 4685/31/2016 and UH 4386/31/2016) and the following industry partners: AbbVie Inc., AstraZeneca UK Ltd, Biogen MA Inc., Bristol Myers Squibb (and Celgene Corporation & Celgene International II Sàrl), Genentech Inc., Merck Sharp & Dohme LCC, Pfizer Inc., GlaxoSmithKline Intellectual Property Development Ltd., Sanofi US Services Inc., Maze Therapeutics Inc., Janssen Biotech Inc, Novartis Pharma AG, and Boehringer Ingelheim International GmbH. Following biobanks are acknowledged for delivering biobank samples to

FinnGen: Auria Biobank (www.auria.fi/biopankki), THL Biobank (www.thl.fi/biobank), Helsinki Biobank (www.helsinginbiopankki.fi), Biobank Borealis of Northern Finland (https://www.ppshp.fi/Tutkimus-ja-opetus/Biopankki/Pages/Biobank-Borealis-briefly-in-English.aspx), Finnish Clinical Biobank Tampere (www.tays.fi/en-US/Research_and_development/Finnish_Clinical_Biobank_Tampere), Biobank of Eastern Finland (www.ita-suomenbiopankki.fi/en), Central Finland Biobank (www.ksshp.fi/fi-FI/Potilaalle/Biopankki), Finnish Red Cross Blood Service Biobank (www.veripalvelu.fi/verenluovutus/biopankkitoiminta), Terveystalo Biobank (www.terveystalo.com/fi/Yritystietoa/Terveystalo-Biopankki/Biopankki/) and Arctic Biobank (https://www.oulu.fi/en/university/faculties-and-units/faculty-medicine/northern-finland-birth-cohorts-and-arctic-biobank). All Finnish Biobanks are members of BBMRI.fi infrastructure (www.bbmri.fi). Finnish Biobank Cooperative -FINBB (https://finbb.fi/) is the coordinator of BBMRI-ERIC operations in Finland. The Finnish biobank data can be accessed through the Fingenious® services (https://site.fingenious.fi/en/) managed by FINBB. The team of investigators in FinnGen are listed in Table S11.

We want to thank the Genes & Health Research Team (in alphabetical order by surname): Shaheen Akhtar, Mohammad Anwar, Omar Asgar, Samina Ashraf, Saeed Bidi, Gerome Breen, James Broster, Raymond Chung, David Collier, Charles J Curtis, Shabana Chaudhary, Grainne Colligan, Panos Deloukas, Ceri Durham, Faiza Durrani, Fabiola Eto, Sarah Finer, Joseph Gafton, Ana Angel,

Chris Griffiths, Joanne Harvey, Teng Heng, Sam Hodgson, Qin Qin Huang, Matt Hurles, Karen A Hunt, Shapna Hussain, Kamrul Islam, Vivek Iyer, Benjamin M Jacobs, Georgios Kalantzis, Ahsan Khan, Claudia Langenberg, Cath Lavery, Sang Hyuck Lee, Daniel MacArthur, Sidra Malik, Daniel Malawsky, Hilary Martin, Dan Mason, Rohini Mathur, Mohammed Bodrul Mazid, John McDermott, Caroline Morton, Bill Newman, Elizabeth Owor, Asma Qureshi, Shwetha Ramachandrappa, Mehru Raza, Jessry Russell, Nishat Safa, Miriam Samuel, Moneeza Siddiqui, Michael Simpson, John Solly, Marie Spreckley. Daniel Stow, Michael Taylor, Richard C Trembath, Karen Tricker, David A van Heel, Klaudia Walter, Caroline Winckley, Suzanne Wood, John Wright, Ishevanhu Zengeya, Julia Zöllner.

Most of all we thank all of the volunteers participating in Genes & Health.

Supplemental references

1. Moore, C.M., Jacobson, S.A., and Fingerlin, T.E. (2019). Power and Sample Size Calculations for Genetic Association Studies in the Presence of Genetic Model Misspecification. Hum. Hered. *84*, 256–271.

2. Futuyma, D.J. (1986). Evolutionary Biology (Sinauer Associates).

3. Heyne, H.O., Karjalainen, J., Karczewski, K.J., Lemmelä, S.M., Zhou, W., FinnGen, Havulinna, A.S., Kurki, M., Rehm, H.L., Palotie, A., et al. (2023). Mono- and biallelic variant effects on disease at biobank scale. Nature *613*, 519–525.

4. Kurki, M.I., Karjalainen, J., Palta, P., Sipilä, T.P., Kristiansson, K., Donner, K.M., Reeve, M.P., Laivuori, H., Aavikko, M., Kaunisto, M.A., et al. (2023). FinnGen provides genetic insights from a well-phenotyped isolated population. Nature *613*, 508–518.

5. Narasimhan, V.M., Hunt, K.A., Mason, D., Baker, C.L., Karczewski, K.J., Barnes, M.R., Barnett, A.H., Bates, C., Bellary, S., Bockett, N.A., et al. (2016). Health and population effects of rare gene knockouts in adult humans with related parents. Science *352*, 474–477.

6. Li, H. (2013). Aligning sequence reads, clone sequences and assembly contigs with BWA-MEM. arXiv [q-bio.GN].

7. Poplin, R., Ruano-Rubio, V., DePristo, M.A., Fennell, T.J., Carneiro, M.O., Van der Auwera, G.A., Kling, D.E., Gauthier, L.D., Levy-Moonshine, A., Roazen, D., et al. (2017). Scaling accurate genetic variant discovery to tens of thousands of samples. Genomics.

8. McLaren, W., Gil, L., Hunt, S.E., Riat, H.S., Ritchie, G.R.S., Thormann, A., Flicek, P., and Cunningham, F. (2016). The Ensembl Variant Effect Predictor. Genome Biol. *17*, 122.

9. 1000 Genomes Project Consortium, Auton, A., Brooks, L.D., Durbin, R.M., Garrison, E.P., Kang, H.M., Korbel, J.O., Marchini, J.L., McCarthy, S., McVean, G.A., et al. (2015). A global reference for human genetic variation. Nature *526*, 68–74.

10. Bergström, A., McCarthy, S.A., Hui, R., Almarri, M.A., Ayub, Q., Danecek, P., Chen, Y., Felkel, S., Hallast, P., Kamm, J., et al. (2020). Insights into human genetic variation and population history from 929 diverse genomes. Science *367*. https://doi.org/10.1126/science.aay5012.

11. Meyer, H.V. (2020). plinkQC: R package for quality control of plink genetic datasets (Github).

12. McInnes, L., Healy, J., and Melville, J. (2018). UMAP: Uniform Manifold Approximation and Projection for Dimension Reduction. arXiv [stat.ML].

13. Manichaikul, A., Mychaleckyj, J.C., Rich, S.S., Daly, K., Sale, M., and Chen, W.-M. (2010). Robust relationship inference in genome-wide association studies. Bioinformatics *26*, 2867–2873.

14. Wright, C.F., West, B., Tuke, M., Jones, S.E., Patel, K., Laver, T.W., Beaumont,

R.N., Tyrrell, J., Wood, A.R., Frayling, T.M., et al. (2019). Assessing the Pathogenicity, Penetrance, and Expressivity of Putative Disease-Causing Variants in a Population Setting. Am. J. Hum. Genet. *104*, 275–286.

15. Loh, P.-R., Danecek, P., Palamara, P.F., Fuchsberger, C., A Reshef, Y., K Finucane, H., Schoenherr, S., Forer, L., McCarthy, S., Abecasis, G.R., et al. (2016). Reference-based phasing using the Haplotype Reference Consortium panel. Nat. Genet. *48*, 1443–1448.

16. Malawsky, D.S., van Walree, E., Jacobs, B.M., Heng, T.H., Huang, Q.Q., Sabir, A.H., Rahman, S., Sharif, S.M., Khan, A., Mirkov, M.U., et al. (2023). Influence of autozygosity on common disease risk across the phenotypic spectrum. bioRxiv. https://doi.org/10.1101/2023.02.01.23285346.

17. Jacobs, B.M., Stow, D., Hodgson, S., Zöllner, J., Samuel, M., Kanoni, S., Bidi, S., Genes & Health Research Team, Walter, K., Langenberg, C., et al. (2024). Genetic architecture of routinely acquired blood tests in a British South Asian cohort. Nat. Commun. *15*, 8929.

18. Akbaş, A., Kılınç, F., Şener, S., and Hayran, Y. (2023). Vitamin D levels in patients with seborrheic dermatitis. Rev. Assoc. Med. Bras. *69*, e20230022.

19. Dimitrova, J. (2013). Study of the level of 25-hydroxyvitamin D in patients with seborrheic dermatitis. Scr. Sci. Medica *45*, 75.

20. Rahimi, S., Nemati, N., and Shafaei-Tonekaboni, S.S. (2021). Serum levels of 25-hydroxyvitamin D in patients with seborrheic dermatitis: A case-control study. Dermatol. Res. Pract. *2021*, 6623271.

21. Ao, T., Kikuta, J., and Ishii, M. (2021). The effects of vitamin D on immune system and inflammatory diseases. Biomolecules *11*, 1624.

22. Malawsky, D.S., van Walree, E., Jacobs, B.M., Heng, T.H., Huang, Q.Q., Sabir, A.H., Rahman, S., Sharif, S.M., Khan, A., Mirkov, M.U., et al. (2023). Influence of autozygosity on common disease risk across the phenotypic spectrum. Cell *186*, 4514–4527.e14.
