# Supplementary material for: Widespread recessive effects on common diseases in a cohort of 44,000 British Pakistanis and Bangladeshis with high autozygosity
Source: Am J Hum Genet. 2025 Apr 29;112(6):1316–29. doi: 10.1016/j.ajhg.2025.03.020 (PMC12256797; doi:10.1016/j.ajhg.2025.03.020)
Supplement: Document S2. Article plus supplemental information [file mmc4.pdf]

Widespread recessive effects on common diseases in a cohort of 44,000 British Pakistanis and Bangladeshis with high autozygosity

Graphical abstract

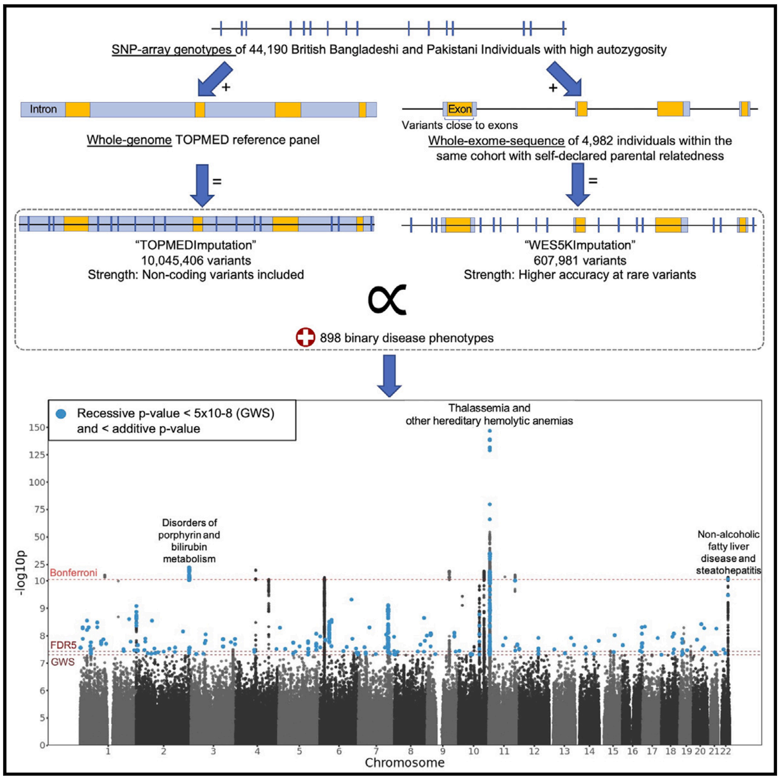

Authors

Teng Hiang Heng, Klaudia Walter, Qin Qin Huang, ..., Sarah Finer, David A. van Heel, Hilary C. Martin

Correspondence

teng-hiang.heng@sanger.ac.uk (T.H.H.),
hilary.martin@sanger.ac.uk (H.C.M.)

We leveraged the increased homozygosity in the Genes & Health cohort to perform recessive association testing between 898 diseases and 10,045,406 whole-genome imputed variants, as well as 607,981 whole-exome imputed variants. We identified 185 independent loci and described several examples. This motivates interrogating recessive effects on common diseases more widely.

Heng et al., 2025, The American Journal of Human Genetics *112*, 1316–1329
June 5, 2025 © 2025 The Author(s). Published by Elsevier Inc. on behalf of American Society of Human Genetics.
https://doi.org/10.1016/j.ajhg.2025.03.020

ARTICLE

Widespread recessive effects on common diseases in a cohort of 44,000 British Pakistanis and Bangladeshis with high autozygosity

Teng Hiang Heng,[1,*] Klaudia Walter,[1] Qin Qin Huang,[1] Juha Karjalainen,[2] Mark J. Daly,[2] Henrike O. Heyne,[2,3] FinnGen, Daniel S. Malawsky,[1] Georgios Kalantzis,[1] Genes & Health Research Team, Sarah Finer,[4] David A. van Heel,[5] and Hilary C. Martin[1,*]

Summary

Genetic association studies have focused on testing additive models in cohorts with European ancestry. Little is known about recessive effects on common diseases, specifically for non-European ancestry. Genes & Health is a cohort of British Pakistani and Bangladeshi individuals with elevated rates of consanguinity and endogamy, making it suitable to study recessive effects. We imputed variants into a genotyped dataset ($n = 44{,}190$) by using two reference panels: a set of 4,982 whole-exome sequences from within the cohort and the Trans-Omics for Precision Medicine (TOPMed-r2) panel. We performed association testing with 898 diseases from electronic health records. 185 independent loci reached genome-wide significance ($p < 5 \times 10^{-8}$) under the recessive model, with p values lower than under the additive model, and >40% of these were novel. 140 loci demonstrated nominally significant ($p < 0.05$) dominance deviation p values, confirming a recessive association pattern. Sixteen loci in three clusters were significant at a Bonferroni threshold, accounting for multiple phenotypes tested ($p < 5.4 \times 10^{-12}$). In FinnGen, we replicated 44% of the expected number of Bonferroni-significant loci we were powered to replicate, at least one from each cluster, including an intronic variant in patatin-like phospholipase domain-containing protein 3 (*PNPLA3*; rs66812091) and non-alcoholic fatty liver disease, a previously reported additive association. We present evidence suggesting that the association is recessive instead (odds ratio [OR] = 1.3, recessive $p = 2 \times 10^{-12}$, additive $p = 2 \times 10^{-11}$, dominance deviation $p = 3 \times 10^{-2}$, and FinnGen recessive OR = 1.3 and $p = 6 \times 10^{-12}$). We identified a novel protective recessive association between a missense variant in *SGLT4* (rs61746559), a sodium-glucose transporter with a possible role in the renin-angiotensin-aldosterone system, and hypertension (OR = 0.2, $p = 3 \times 10^{-8}$, dominance deviation $p = 7 \times 10^{-6}$). These results motivate interrogating recessive effects on common diseases more widely.

Introduction

Recessive effects in humans have been primarily studied in the context of rare, monogenic disorders, and little is known about recessiveness in common diseases and complex traits.[1] Identifying variants with recessive associations with diseases could improve polygenic risk scoring, provide better insight into gene and variant function, improve understanding of disease pathophysiology, and allow for the identification of novel drug targets.[2–4]

The effects of genetic variation on common complex phenotypes are typically discovered through genome-wide association studies (GWASs),[5–7] where an additive model is predominantly tested. However, applying a recessive model has allowed for the discovery of associations that would have been otherwise missed under conventional additive testing. For example, Heyne et al.[8] performed recessive tests on 44,370 variants and 2,444 diseases in the FinnGen project from Finland and identified 31 loci at genome-wide significance (GWS; $p < 5 \times 10^{-8}$) where the associations were more significant in the recessive model than in the additive model. Notably, of the 20 findings further validated, 13 loci would have been missed with the additive model alone. Similarly, Guindo-Martinez et al.[9] performed non-additive association testing in 62,281 subjects across 22 age-related diseases, and among 26 novel loci, four were identified only with the recessive model. Palmer et al.[1] systematically quantified the contribution of dominance deviations (deviation from the additive pattern of inheritance) to heritability across 1,060 common traits in the UK Biobank (UKBB; $n = 361{,}194$). They identified non-additive effects of 183 phenotype-locus pairs across the phenotypic spectrum but concluded that, overall, non-additive effects contribute very little to heritability. Collectively, these results suggest that many recessive associations on common traits still remain to be found through the application of non-additive testing, including with rare variants, where the additive model would be more likely to miss recessive effects.

With the construction of large-scale biobanks, it is now possible to study the recessive contribution to common diseases.[8,10] However, in outbred populations, very large sample sizes are required for adequate power to test recessive effects rather than additive effects, particularly for rare variants. The power to detect recessive effects is expected to be increased in bottlenecked populations such

[1]Wellcome Sanger Institute, Wellcome Genome Campus, Hinxton CB10 1SA, UK; [2]Broad Institute, 415 Main Street, Cambridge, MA 02142, USA; [3]Hasso Plattner Institute, 14482 Potsdam, Germany; [4]Wolfson Institute for Population Health, Queen Mary University of London, London E1 4NS, UK; [5]Blizard Institute, Queen Mary University of London, London E1 2AT, UK
*Correspondence: teng-hiang.heng@sanger.ac.uk (T.H.H.), hilary.martin@sanger.ac.uk (H.C.M.)
https://doi.org/10.1016/j.ajhg.2025.03.020.
© 2025 The Author(s). Published by Elsevier Inc. on behalf of American Society of Human Genetics.
This is an open access article under the CC BY license (http://creativecommons.org/licenses/by/4.0/).

as Finland, where recessive variants may rise in frequency due to founder effects,[8,11] or in populations enriched for consanguinity and, therefore, increased homozygosity. We showed in simulations that the power to find recessive effects is boosted both by explicit testing of a recessive model and by increased homozygosity (Note S1).

Genes & Health (G&H) is a community-based cohort of, at present, ~60,000 individuals of British Bangladeshi and Pakistani ancestry with genetic data and linked electronic health records (EHRs).[12] The cohort has a high rate of consanguinity (32% are offspring of second cousins or closer).[13] Furthermore, British Pakistanis, who comprise 40% of the cohort, have been previously found to have high levels of endogamy as a result of the biraderi system, and multiple bottlenecked subpopulations display elevated levels of identity-by-descent (IBD) sharing, more than 10–20 times the level found in the Finnish population.[13,14] We therefore hypothesized that the increased IBD sharing within these subgroups in G&H might allow reasonable quality imputation of variants even from a relatively small sample size of individuals from the same cohort. From this imputation, we would be able to take advantage of the increased homozygosity to test for recessive effects.

We leveraged genotype chip data on 44,000 G&H individuals, of whom around 5,000 also had whole-exome sequencing (WES) data. First, we imputed variants from the exome-sequenced individuals into the larger genotyped G&H cohort. This was inspired by Barton et al.,[15] who boosted the power for association testing of rare coding variants by building a within-cohort reference panel from 49,960 WES samples in the UK Biobank and imputing variants into the larger genotyped cohort ($n =$ ~500,000). We also used an additional imputation reference panel, the Trans-Omics for Precision Medicine (TOPMED-r2) panel (97,256 individuals, including 644 with South Asian ancestry)[16] to perform whole-genome imputation to study recessive effects in the noncoding regions. Using these two imputed datasets, we performed association testing with binary phenotypes curated from the EHRs, focusing on detecting recessive effects. We then characterized the recessive associations that we identified, systematically testing for replication and searching for literature support for each finding. In order to be objective when deciding which of our findings are "novel," we have applied a standardized set of criteria, which are summarized in Note S10.

Methods

Summary of data collection

Participants were recruited into the G&H cohort with individual written informed consent, and data analysis is compliant with the General Data Protection Regulation (GDPR). The study has ethical approval (14/LO/1240) from the London South East NRES Committee of the Health Research Authority. More details are described in the supplemental acknowledgments. G&H data are available for analysis in a secure trusted research environment. Application can be made to the G&H executive: https://www.genesandhealth.org/researchers/apply-for-access/. Information on how to access FinnGen data can be found here: https://www.finngen.fi/en/access_results.

Preparation of genetic data

Preparation of the genotyped data

Detailed quality control (QC) of the genetic data is described in Note S2. Genome-wide genotyping was performed with the Illumina Global Screening Array (GSAv3EAMD, build 38), and these data were used as the imputation backbone. Initial QC has been described in Huang et al.[17] From the genotyped 44,396 individuals, we inferred 44,190 individuals to be of either Bangladeshi or Pakistani genetic ancestry (Note S3), and downstream analyses were restricted to these individuals. Another round of variant filters was applied to include only autosomal, bi-allelic SNPs with a ≥99% call rate. The Pakistani subgroup has high autozygosity and strong population structure, whereas the Bangladeshi subgroup has minimal structure and much less autozygosity,[18] so to avoid excluding too many high-quality variants as a result of failure on a standard test for Hardy-Weinberg equilibrium (HWE), we performed the HWE test (with PLINK 1.9)[19] only in the Bangladeshi subgroup, and the variants that failed a p value threshold of 10^{-6} in Bangladeshis were then excluded from the entire dataset. Variants with a minor-allele frequency (MAF) > 0.1% were included from the imputation backbone, which resulted in 469,678 variants that were then phased with EAGLE2 (Algorithm option Kpbwt = 20,000).[20]

Preparation of the within-cohort imputation panel

Exome sequencing was performed with Agilent v.5 capture kits on a subset of 5,236 individuals who self-declared as having consanguineous parents. Mapping, calling, and initial QC included excluding samples with sex discrepancies and <10× on-target coverage and applying the following variant filters by using bcftools[21]: "QD < 2.0 || FS > 30 || MQ < 40.0 || MQRankSum < −12.5 || ReadPosRankSum < −8.0" for SNPs and "QD < 2.0 || FS > 30 || ReadPosRankSum < −20.0" for insertions or deletions (indels; referenced from the G&H September 2019 summary files, please see web resources). We restricted the analysis to the 5,073 individuals who were genetically inferred to be of Bangladeshi or Pakistani ancestry from their array data. Then, we set genotypes that had a genotype quality (GQ) < 20, a p value from a binomial test for allele depth at heterozygous sites (binomAD) < 10^{-2}, or a depth ≤ 7 to missing. Variants were excluded if they had a post-genotype QC call rate < 70%. 91 samples with high missingness or high discordance with their array data were excluded, leaving 4,982 samples (Note S4). The WES data were then merged with the SNP-array data and phased with EAGLE2 (Kpbwt = 20,000) to form a reference panel for imputation.

Imputation

We imputed the G&H data against two different imputation panels. Firstly, variants were imputed from the within-cohort whole-exome reference panel (described above) into the individuals without WES data with Minimac4.[22] To assess the imputation accuracy and determine an imputed r^2 cutoff, ten "leave-10%-out" trials were performed (Note S4). We retained variants with an imputed $r^2 \geq 0.5$ and at least three individuals with a homozygous genotype. This is referred to as the "WES5Kimputation" dataset. The SNP-array data were also submitted to the TOPMed-r2 Minimac4 1.5.7 Imputation Server[16,22,23] for whole-genome imputation against the

TOPMED-r2 panel. The same post-imputation filters were applied, and this is referred to as the "TOPMEDimputation."

Variant annotation

Variants were annotated with Ensembl's Variant Effect Predictor (VEP) v.107. For each variant, the worst consequence for any transcript was extracted for subsequent analyses.

Phenotype curation

Two lists of phenotypes were curated from participants' EHRs, and phenotype information was encoded as a binary, with "1" coding for a case and "0" coding for a control. A list of 237 custom phenotypes was compiled manually, and a second set of 1,281 phenotypes were defined based on International Classification of Disease (ICD10) codes. Further detail is described in Malawsky et al. (methods section phenotypic data harmonization and preparation for G&H).[18] We retained phenotypes with ≥ 30 cases and also classified them into those that affected both sexes or were sex-specific (i.e., occurred only in females or males). For sex-specific phenotypes, the cohort was filtered to the relevant sex for testing. This resulted in 898 phenotypes.

Association testing

We performed association testing by using the two-step pipeline of REGENIE.[24] In step 1, we fit the model by using variants from the SNP array; we used the leave-one-out cross-validation (LOOCV) scheme and a genotype block size of 1,000. Step 2 was performed under the additive model and the recessive model at a genotype block size of 1,000 and a p value threshold of 0.05, below which the approximate Firth correction was applied. The covariates included age, sex, age^2, age × sex, age^2 × sex, and the first ten principal components (PCs) from the principal-component analysis (PCA) on unrelated G&H individuals (Note S3).

We defined significant recessive associations as tests with $p < 5 \times 10^{-8}$ (GWS) and with a p value lower under the recessive model than under the additive model. We excluded the human leukocyte antigen (HLA) region as a result of complex linkage disequilibrium (LD; chr6: 25–35 Mb) and then defined independent loci with the following steps: (1) for each phenotype with significant tests, we identified the test with the most significant recessive p value as the lead variant. (2) We calculated the LD r^2 by using PLINK 1.9 between the lead variant and variants within a ±1.5 Mb window of it. Variants with an LD $r^2 \geq 0.25$ with the lead variant were defined as being part of the same locus, according to previous work from FinnGen.[8,11] (3) We then identified the next most significant variant among the remaining variants that are not part of the locus defined in the previous steps and repeated the loop.

We calculated dominance deviation p values to assess the evidence that our significant associations detected under the recessive model were really recessive. Specifically, for the lead variants of the significant recessive associations, we ran logistic regression in R, controlling for the same covariates, performing genotypic tests with 2 degrees of freedom as well as additive and recessive tests for comparison. A description of how this was performed and detailed comparisons of the results with those from REGENIE are described in Note S8. We also noted that pairs of individuals carrying the same homozygous genotype at these significant variants were significantly more likely to be first-degree relatives than expected by chance (Note S3). However, repeating the association testing using only unrelated individuals found similar results (Figure S5), suggesting REGENIE is adequately controlling for this relatedness.

Testing for replication in FinnGen and GERA

FinnGen is a public-private collaboration to profile the genomic and digital healthcare data of ~500,000 Finnish individuals, with the goal of uncovering novel biological and therapeutic insights into human diseases. As a recessive testing pipeline has been built in the cohort before,[8] we used it as an independent cohort to assess replication. More details of the cohort are described in Note S12. Phenotypes were matched manually to FinnGen binary phenotypes curated from Finnish health registers (Table S7). The variants from FinnGen release 10 were then tested with the phenotypes using REGENIE under the recessive model, controlling for the covariates sex, age, 10 PCs, and genotyping batch, as described for release 10 on https://www.finngen.fi/.[11] For each significant locus in G&H, we first identified proxy variants as variants that are within the window described above (±1.5 Mb from the lead variant with an LD $r^2 \geq 0.25$. For a more stringent assessment of replicability, we applied the locus definitions from Huang et al.[17] and repeated the analysis (see Note S9). We defined a locus as replicating (or "transferable") in FinnGen if any of the lead or proxy variants had the same direction of effect as observed in G&H at a nominally significant $p < 0.05$ when tested in a recessive model with a similar trait. To compare the number of loci that replicated to what we might have expected to replicate given the power in FinnGen, we calculated a power-adjusted transferability (PAT) ratio.[17] Specifically, for each locus, we used the effect size of the lead variant in G&H, the allele frequency (AF) of the lead variant in FinnGen, and the case rate and sample size in FinnGen to estimate the power for the test with the genpwr R package.[25] The expected number of transferable loci was calculated as the sum of the power estimates across all the loci. The PAT ratio was then calculated by dividing the observed number of transferred loci by the expected number.

Publicly available summary statistics from recessive testing in the Genetic Epidemiology Research on Aging (GERA) cohort by Guindo-Martinez et al.[9] were accessed on December 14, 2023. Phenotypes were matched manually (Table S8), and the PAT ratio was calculated as described above, through the use of AFs and case rates in GERA.

Results

Recessive association testing identifies 185 loci

Using exome-sequencing data from 4,982 individuals from G&H, we generated a reference panel, which allowed us to impute 605,263 variants, including rare exonic variants, into the larger cohort of 44,186 individuals from that cohort. Simultaneously, we carried out a whole-genome imputation of 10,045,406 variants using the TOPMED panel. We then tested these variants for recessive associations with 898 phenotypes. We identified 185 unique loci where the lead variant had a genome-wide-significant recessive p value ($<5 \times 10^{-8}$) that is smaller than the additive p value (Figure 1; Table S5). At a stringent Bonferroni cutoff ($0.05/9{,}197{,}933{,}046$ tests $= 5.4 \times 10^{-12}$; Note S5), 16 loci remained. 144 loci passed a more lenient

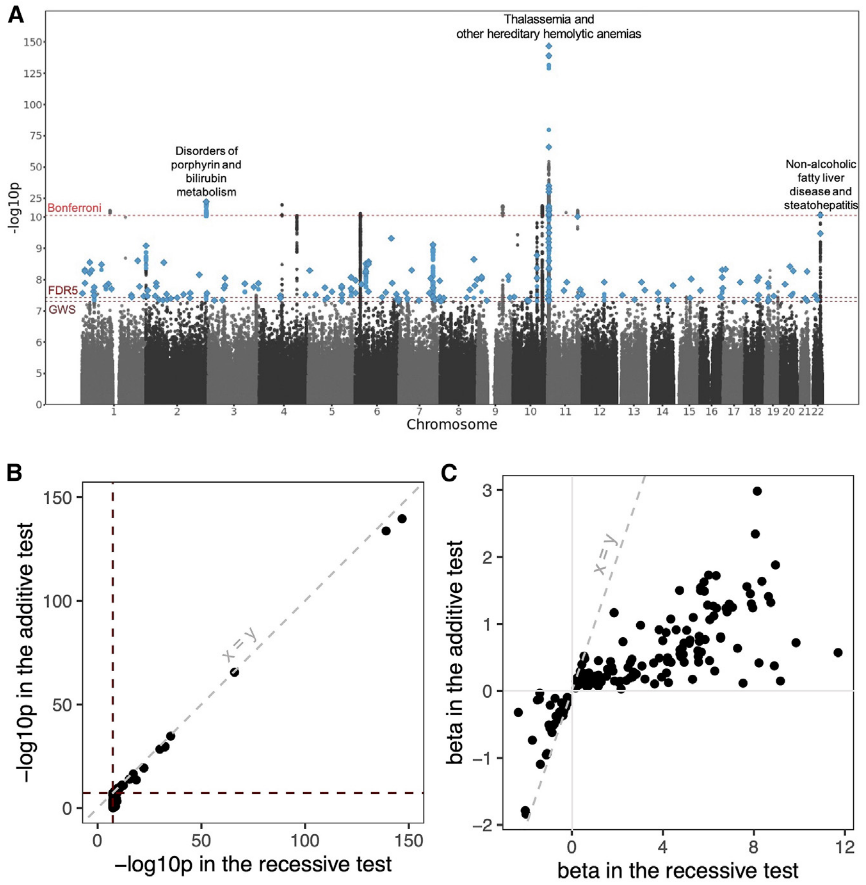

Figure 1. The 185 loci identified at genome-wide significance in recessive association tests

(A) Manhattan plot of the results from recessive tests performed. Dashed horizontal lines represent the various p value cutoffs: genome-wide significance (GWS; $p < 5 \times 10^{-8}$), for a false discovery rate of 5% (FDR5; $p < 3.7 \times 10^{-8}$), and the Bonferroni-corrected cutoff (Bonferroni; $p < 5.5 \times 10^{-12}$). Blue points represent recessive tests passing the genome-wide significance threshold where the recessive p value is more significant than the additive p value, and diamonds represent lead variants defined as described in the methods. The clusters containing Bonferroni-significant associations are labeled with the phenotypes with which they are associated.

(B) For the lead variants, the −log10 p from additive testing against −log10 p from recessive testing. The horizontal line indicates the genome-wide significance threshold, and the diagonal line is $y = x$.

(C) The effect size (beta) under the additive versus recessive model for the lead variants. The additive beta is the log of the change in odds for carrying one alternate allele, while the recessive beta is the log of the change in odds for the homozygous carriers of the alternate allele compared to the rest of the cohort (reference homozygotes and heterozygotes combined).

Benjamini-Hochberg cutoff for a false discovery rate of 5% (FDR 5%) ($p < 3.7 \times 10^{-8}$, so close to the GWS threshold). Notably, the 16 Bonferroni-significant findings can be found in three clusters (Figure 1; Table 1), corresponding to (1) non-alcoholic fatty liver disease and steatohepatitis (NAFLD) (one locus; lead SNP chr22:43,939,790 [GRCh38]), (2) disorders of porphyrin and bilirubin metabolism (one locus, found with both imputation panels; lead SNP chr2:233,763,993), and (3) thalassemia and other hereditary hemolytic anemias (13 loci, one of which was found with both imputation panels; lead SNPs at chr11:4,908,482–5,544,800).

Of the lead variants at the 185 recessive loci, 152 (82%) were not genome-wide significant in the additive test (Figure 1B), suggesting that they would have been missed in conventional additive GWASs. The associations that were not genome-wide significant in the additive test tended to be with rarer variants (Figure S15A). The effect sizes in the recessive tests for these lead variants also tend to have a larger magnitude than in the additive tests (Figure 1C). 76% (140) of these lead variants had nominally significant dominance deviation p values.

These 185 recessive loci include loci that were significant in either or both of the imputation panels. Twenty-nine of the lead variants were successfully tested in both imputation sets. At these, the effect sizes (Figure 2B) and AFs (Figure 2C) correlated well between the two imputation panels, though interestingly, a small subset of findings significant with the WES5Kimputation had much less significant p values when their TOPMEDimputation genotypes were tested (Figure 2A). These findings corresponded to variants with lower imputed r^2 values in the TOPMEDimputation compared to the WES5Kimputation (Figure 2D), suggesting that higher confidence in the imputation when using the in-house panel improved the sensitivity of the testing through more accurate prediction of the genotypes.

Replication of significant findings in other cohorts

We sought to test how many of the 185 loci could be replicated in two other cohorts suitable for recessive association testing, FinnGen and GERA. 133 loci could be found in FinnGen, meaning the lead variant of the locus could be tested with a comparable phenotype in FinnGen. Of these 133 lead variants, 82 had the same direction of effect in the recessive tests of both cohorts. This is significantly more than the number expected by chance (one-sided exact binomial test, $p = 0.0045$).

Next, we calculated the PAT ratio,[17] a quantitative measure of how many loci can be replicated in FinnGen relative to the power in this independent cohort; it takes into account AFs, case rates, and sample sizes. We expected to replicate 129 of the 133 examined

Table 1. Recessive findings that passed Bonferroni significance

				Genes & Health								FinnGen				
						Recessive		Additive					Recessive		Additive	
Gene	Lead variant	Consequence	Phenotype	Imputation	AF	OR	*p*	OR	*p*	Δ log10 *p*	DD *p*	Power	OR	*p*	OR	*p*
UGT1A6	chr2:233763993G>T	intron	E80 (disorders of porphyrin and bilirubin metabolism)	WES5K	0.53	6.4	5×10^{-23}	3.2	4×10^{-20}	2.9	4×10^{-3}	1	5.8	2×10^{-74}	3.0	4×10^{-65}
UGT1A6	chr2:233763993G>T	intron	E80 (disorders of porphyrin and bilirubin metabolism)	TOPMED	0.53	6.4	5×10^{-23}	3.2	4×10^{-20}	2.9	4×10^{-3}	1	5.8	2×10^{-74}	3.0	4×10^{-65}
UGT1A6	chr11:5216054A>G	intergenic	D56 (thalassemia)	TOPMED	0.76	0.6	5×10^{-16}	0.7	1×10^{-14}	1.4	3×10^{-3}	1	0.7[a]	4×10^{-3}[a]	not reported	not reported
OR52D1	chr11:5489111C>T	synonymous	D56 (thalassemia)	WES5K	0.81	0.6	4×10^{-13}	0.7	1×10^{-14}	1.5	5×10^{-3}	0.9	not replicated	not replicated	not reported	not reported
HBE1	chr11:5478732G>C	intron	D56 (thalassemia)	TOPMED	0.81	0.6	2×10^{-16}	0.7	1×10^{-14}	1.7	6×10^{-4}	1	0.7[b]	8×10^{-5}[b]	not reported	not reported
HBE1	chr11:5330439C>T	intron	D56 (thalassemia)	TOPMED	0.71	0.4	2×10^{-12}	0.6	7×10^{-12}	0.6	1×10^{-2}	1	not replicated	not replicated	not reported	not reported
HBE1	chr11:5367606A>C	intron	D58 (other hereditary hemolytic anemias)	TOPMED	0.98	0.3	6×10^{-36}	0.4	2×10^{-35}	0.5	2×10^{-3}	1	not replicated	not replicated	not reported	not reported
HBG1	chr11:5247392T>G	downstream gene	D58 (other hereditary hemolytic anemias)	TOPMED	0.61	0.4	2×10^{-19}	0.6	3×10^{-14}	5.1	9×10^{-5}	1	not replicated	not replicated	not reported	not reported
MMP26	chr11:4931228C>A	intron	D58 (other hereditary hemolytic anemias)	TOPMED	0.79	0.4	6×10^{-18}	0.6	3×10^{-17}	0.7	4×10^{-3}	1	0.8	3×10^{-2}	not reported	not reported
OR51A7	chr11:4908482G>A	3′ UTR	D58 (other hereditary hemolytic anemias)	TOPMED	0.95	0.3	1×10^{-66}	0.3	3×10^{-66}	0.5	2×10^{-8}	1	not replicated	not replicated	not reported	not reported
OR51V1	chr11:5197578AAT>A	downstream gene	D58 (other hereditary hemolytic anemias)	TOPMED	0.55	0.2	1×10^{-30}	0.5	4×10^{-29}	1.6	7×10^{-7}	1	not replicated	not replicated	not reported	not reported
OR52E1	chr11:5067264A>G	upstream gene	D58 (other hereditary hemolytic anemias)	TOPMED	0.99	0.1	9×10^{-140}	0.2	2×10^{-134}	5.4	2×10^{-14}	1	0.1[c]	1×10^{-129}[c]	not reported	not reported

(*Continued on next page*)

Table 1. *Continued*

				Genes & Health								FinnGen				
						Recessive		Additive					Recessive		Additive	
**Gene**	**Lead variant**	**Consequence**	**Phenotype**	**Imputation**	**AF**	**OR**	***p***	**OR**	***p***	**Δ log10 *p***	**DD *p***	**Power**	**OR**	***p***	**OR**	***p***
OR52E2	chr11:5058716C>T	synonymous	D58 (other hereditary hemolytic anemias)	WES5K	0.99	0.1	2×10^{-147}	0.2	3×10^{-140}	7.2	8×10^{-19}	1	not replicated	not replicated	not reported	not reported
OR52H1	chr11:5544800A>G	missense	D58 (other hereditary hemolytic anemias)	WES5K	0.99	0.3	4×10^{-33}	0.4	3×10^{-30}	2.9	4×10^{-4}	1	not replicated	not replicated	not reported	not reported
OR52H1	chr11:5544800A>G	missense	D58 (other hereditary hemolytic anemias)	TOPMED	0.99	0.3	3×10^{-33}	0.4	2×10^{-30}	2.7	4×10^{-4}	1	not replicated	not replicated	not reported	not reported
PNPLA3	chr22:43939790TGG>T	intron	non-alcoholic fatty liver disease and steatohepatitis	TOPMED	0.61	1.3	2×10^{-12}	1.2	2×10^{-11}	0.9	3×10^{-2}	0.9	1.4	6×10^{-12}	not reported	not reported

The allele frequency (AF) and odds ratio (OR) presented are for the alternate allele (second allele in the lead variant ID). Footnotes are added to those that replicated by proxy variant. We checked whether additive results in FinnGen were reported in https://r10.finngen.fi/. We derived the power-adjusted transferability (PAT) ratio for the replication of Bonferroni-significant loci in FinnGene by summing the number of loci that replicated (7) and dividing it by the sum of the expected power for replication at each loci (sum of the "power" column: ∼16); this gave us a PAT of 44%. OR, odds ratio, converted from beta in the test output; Δ log10 *p*, the difference between the −log10 *p* in the recessive test and the −log10 *p* in the additive test; DD *p*, dominance deviation *p* value.

[a]Proxy variant that replicated is chr11:5212606C>G (LD $r^2 = 0.3$).

[b]Proxy variant that replicated is chr11:5483160T>C (LD $r^2 = 0.4$).

[c]Proxy variant that replicated is chr11:5011706A>G (LD $r^2 = 0.9$).

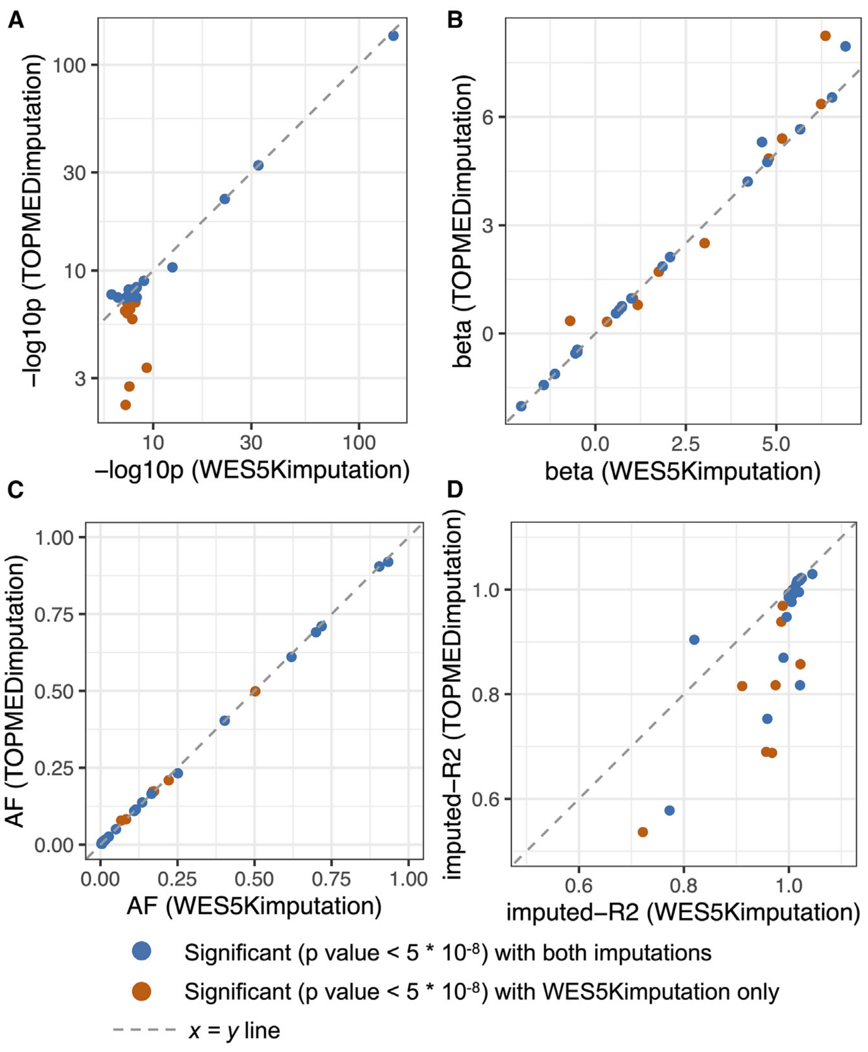

Figure 2. Comparison of results for variants tested using both imputation panels and that are significant in one or both
Scatterplots show the (A) p values ($-\log10\ p$), (B) betas, (C) allele frequencies (AFs), and (D) imputed r^2 values of lead variants.

loci at $p < 0.05$; of these, 30 loci replicated in FinnGen, resulting in a PAT ratio of 23%. This suggests that a $p < 5 \times 10^{-8}$ cutoff for significance in G&H is too lenient, given the number of phenotypes tested. Restricting to the Bonferroni-significant findings, the PAT ratio increased to 44%. The loci associated with NAFLD and with disorders of porphyrin and bilirubin both replicated. For the cluster of loci associated with thalassemia and other hereditary hemolytic anemia, four out of 13 loci replicated, while eight of the remaining nine loci had the same direction of effect in both cohorts (although we note that we were not considering identical phenotypes in FinnGen since they were not available; see Table S8). We also assessed replication with a variety of more stringent cutoffs, such as applying stricter locus definitions (which would inflate the number of independent findings), considering only lead variants for replication, and changing p value thresholds, as shown in Note S9. The broad conclusions remain the same: about ~40% to ~70% of loci replicate by their lead SNP alone, depending on the replication criteria (Table S11), and Bonferroni-significant loci had a higher percentage of replication than genome-wide-significant loci and were more robust to more stringent thresholds for replication.

We also assessed replication in the GERA cohort. The 22 phenotypes tested by GERA restricted the number of phenotypes we could match, so only 11 GWS loci could be evaluated. After accounting for the AF, case rate, and sample size differences, we expected to replicate around eight loci. Four loci replicated, resulting in a PAT ratio of 48%.

Finally, we looked up our significant recessive associations in the genome-wide scans performed in Palmer et al.[1] that tested for deviation from the additive effect across traits in ~361,000 unrelated individuals of European ancestry in the UK Biobank. Among our 185 significant associations, 71 were also tested in the UK Biobank (i.e., the leading variant was tested for dominance deviation from the additive effect on a phenotype related to the one tested in G&H). Nine of them showed nominally significant dominance testing results with a concordant direction of effect (Table S5). Of these nine loci, seven replicated in FinnGen, and three of four loci that replicated in GERA replicated here as well.

The Bonferroni-significant recessive associations

The Bonferroni-significant lead variants are listed in Table 1. We summarize these significant associations below.

Firstly, we found a recessive association between rs66812091 (chr22:43939790TGG>T), an intronic variant in *PNPLA3* (patatin-like phospholipase domain-containing protein 3), and NAFLD, with an odds ratio (OR) of 1.3 at a p value of 2×10^{-12}. This p value is an order of magnitude more significant in the recessive test than the additive test (2×10^{-11}). The relationship also had a significant dominance deviation p value of 3×10^{-2} and was replicated in FinnGen with a recessive test OR of 1.3 and a p value of 6×10^{-12}. *PNPLA3* is involved in lipid and fatty acid metabolism and has high expression in adipose and liver tissues.[26,27] This gene's role in fatty acid metabolism and liver function has been further supported by

functional knockout studies in mice.[27,28] This variant has also been shown in additive GWASs to be associated with NAFLD (in the FinnGen release 6 dataset) and deranged liver enzymes (a marker of hepatitis).[29] However, to our knowledge, this is the first study demonstrating that, in fact, one or more of the variants near this gene are likely to have recessive rather than additive effects on NAFLD.

Secondly, an intronic SNP in *UGT1A6*, rs6742078 (chr2: 233763993G>T), identified with both imputation panels, was found to be associated with disorders of porphyrin and bilirubin metabolism. *UGT1A6* encodes a uridine 5′-diphospho-glucuronosyltransferase (UDP-glucuronosyltransferase [UGT]). UGTs are enzymes responsible for the glucuronidation of lipophilic molecules, including bilirubin, into the conjugated, hydrophilic form that can be excreted in the urine.[30] *UGT1A6* has been shown to be associated with bilirubin levels in the GWASs on liver traits and bilirubin levels.[31,32] Mutations in UGTs, specifically *UGT1A1*, cause the well-known autosomal recessive conditions Gilbert's syndrome[33] and Crigler-Najjar syndrome,[33,34] in which affected individuals present with excess unconjugated bilirubin. The findings presented here demonstrate novel recessive associations between another UGT not previously reported and dysfunctional bilirubin clearance.

Thirdly, we found multiple Bonferroni-significant recessive hits in the genomic region chr11:4,908,482–5,544,800 associated with thalassemia and other hereditary hemolytic anemias. Although many variants in this region have been known to associate with these phenotypes (Note S11), among the four hits that replicate in FinnGen, two (chr11:4931228C>A and chr11:5067264A>G) have no reported associations in the literature, additive or otherwise, and can be considered novel; the remaining two hits (chr11:5216054A>G and chr11:5478732G>C) have been associated with multiple anemia traits in additive GWASs,[35,36] and we present support that their underlying association pattern may be recessive instead (Table 1; Note S10). The variants span two hemoglobin genes, hemoglobin epsilon locus (*HBE1*) and hemoglobin gamma a (*HGB1*), as well as the gene *MMP26* and a cluster of olfactory receptor genes. The two hemoglobin genes *HBE1* and *HGB1* encode embryonic and fetal hemoglobin (HbF) subunits, respectively. The gene hemoglobin subunit gamma 2 (*HBG1*) is well established to be associated with β-thalassemia and hemoglobin E disease.[37] β-thalassemia is an inherited anemia caused by dysfunctional production of the β-globin subunit of adult hemoglobin (HbA)[38]; therefore, the persistence of elevated HbF past infancy as a compensatory mechanism is occasionally observed.[39] Genes encoding embryonic hemoglobin and HbF, including *HBE1* and *HGB1*, have been targeted as part of possible therapies in thalassemias, where reactivating these alternative forms of hemoglobin expression may help to supplement low HbA production.[40–42] With regard to the variants in olfactory receptor genes, there is some evidence in the literature reporting on the relationship between olfactory genes and thalassemia. The β-globin gene *HBB*, in which pathogenic variants cause β-thalassemia, is sandwiched between olfactory receptor gene clusters,[43] and β-globin deletions that cause thalassemia have been known to extend to these olfactory genes.[44,45] In addition, one of the olfactory genes in this region, *OR52A1*, contains an enhancer for γ-globin, and variants that disrupt the function of this enhancer may exacerbate anemia in individuals with thalassemia.[46] As the variants we have reported are in close proximity to multiple hemoglobin genes, including *HBB*, we tested if they were in LD with variants in hemoglobin genes but found no such evidence within G&H. We think it is likely that these variants are expression quantitative trait loci (eQTLs) or splice QTLs (sQTLs) for hemoglobin genes. Indeed, we found one additional eQTL, chr11:5489111C>T, the synonymous variant in *OR52D1*, that is an eQTL associated with changes in the expression of *HBG2* in blood, as reported in eQTLGen.[47] However, we cannot robustly test this in the absence of large-scale single-cell eQTL and sQTL datasets because the QTLs might be specific to certain cell types or states.

Recessive findings implicating coding variants provide insight into gene functions

We observed a significant enrichment of recessive associations among coding variants (Note S7). Out of the 10,045,406 variants of the TOPMEDimputation that were tested, 0.044% of coding variants had significant recessive associations compared to 0.011% in noncoding variants (chi-squared test $p = 8.3 \times 10^{-20}$). This is consistent with the expectation that protein-coding variants are more likely to impact gene function and health outcomes and that they have larger effect sizes, leading to better power for detection. Since these coding variants seem likely to be the causal variants at those loci, we discuss several examples here.

Firstly, we see a recessive association between a missense variant (chr1:11796321G>A, rs1801133; c.677C>T [p. Ala222Val]) in the gene encoding methylenetetrahydrofolate reductase (MTHFR) and folate deficiency (OR = 2.1, $p = 5 \times 10^{-9}$, dominance deviation $p = 10^{-3}$). This finding was also replicated in FinnGen (OR = 2.1, $p = 0.02$). MTHFR is an enzyme involved in folate and homocysteine metabolism. After folate is converted to 5,10-methylenetetrahydrofolate (5,10-MTHF), MTHFR reduces 5,10-MTHF to 5-methyltetrahydrofolate (5-MTHF), which is then required as a cosubstrate for the conversion of homocysteine to methionine.[48] The missense variant we report (c.677C>T) has been shown to cause instability in MTHFR, resulting in the accumulation of homocysteine.[49] Indeed, there are reports that this c.677C>T mutation has a recessive effect on homocysteine levels but with a mild heterozygous effect: individuals heterozygous for the c.677C>T mutation have mildly elevated homocysteine levels, while the homozygous individuals have significantly higher levels.[50–52] Functional studies have shown that the enzyme's reduced efficiency from instability caused by the

c.677C>T transition can be compensated with additional folate.[51,53–55] This implies that the reduced enzyme efficiency from the c.677C>T transition results in low folate levels, consistent with our association. Multiple additive GWASs have reported associations between this variant and folate deficiency anemia (in the FinnGen release 6 dataset) or being on folate supplements (in the UK Biobank GWAS round 2 results). However, our study suggests that the underlying pattern of inheritance may be recessive instead. Folate is essential for DNA, RNA, and protein methylation, and fetal neural tube defect is an established consequence of low folate levels in pregnancy.[56] Germane to this, there are several reports (although conflicting) of recessive associations between variants (including c.677C>T) in *MTHFR* and neural tube defects.[57–60]

Secondly, we have found a protective recessive association between a missense variant in solute carrier (SLC) family 5 member 9 (*SLC5A9*) (chr1:48228922G>A, rs61746559) hypertension (OR = 0.2, $p = 3 \times 10^{-8}$, dominance deviation $p = 7 \times 10^{-6}$). SLC5A9 is also known as sodium-glucose transporter 4 (SGLT4) and is a member of the SLC superfamily. Specifically, it is a sodium-dependent glucose transporter of mannose, 1,5-anhydro-D-glucitol, and fructose.[61] Another member of this family is the well-studied SGLT2, a glucose transporter largely expressed in the kidney and which is a target of gliofozins, drugs used for lowering serum glucose levels in individuals with type 2 diabetes.[62] It has been shown that a concomitant benefit of inhibiting SGLT2 in type 2 diabetes is lowering blood pressure,[63–65] possibly from hemodynamic changes in kidney glomeruli[66] and regulating the renin-angiotensin-aldosterone system.[67] SGLT4 may function in a similar manner, as, like SGTL2, it is expressed in the kidneys.[61] Missense mutations might inhibit its function, explaining the relationship we detect with hypertension. Although there is limited information about this gene's function currently[68] to support this hypothesis, there is an additive association between this variant and renin levels,[69] suggesting it might indeed be involved in the renin-angiotensin-aldosterone system. Multiple widely used classes of antihypertensives work on the renin-angiotensin-aldosterone system, which plays a key role in regulating blood pressure.[70]

Quantifying novelty in the recessive findings

For the 185 recessive loci reported, we next systematically searched for associations between the lead variant and the same or related trait in the literature. In an effort to quantify the novelty in our findings, we developed a "literature evidence score" to characterize the strength of the literature support for each finding (the higher the score, the stronger the evidence). The details of the approach are described in Note S10. In summary, ~40%–80% of our reported associations are novel, depending on whether a strict (no reported association to a related trait) or lenient (no reported association to the same trait) definition of novelty is used. We found that the strength of the support in the literature was associated with a smaller difference in the recessive and additive p values for the test (linear regression of delta log10 p against our literature evidence score; slope: −0.32, $p = 5.4 \times 10^{-5}$; Figure S18)—in other words, associations that seem more likely to be truly recessive are less likely to have support already in the literature. This reiterates the importance of performing recessive testing on top of just additive testing and in cohorts with better power for the recessive tests, as these findings would potentially have been missed from additive testing alone.

Discussion

After performing recessive association testing on variants imputed from both a within-cohort exonic reference panel and the whole-genome TOPMED reference panel, 185 unique loci were identified at GWS, where the recessive association was more significant than the additive association. After Bonferroni multiple testing correction, 16 loci in three clusters remained. After adjusting for changes in case rates, AFs, and sample size in the independent cohort FinnGen, we replicated 44% of the expected number of Bonferroni-significant loci we would be powered to replicate, including at least one locus from each cluster. We also identified recessive associations at loci previously thought to be additive. Examples include the association between rs66812091 and NAFLD and that between rs1801133 and folate deficiency. Notably, we report a novel recessive association between a missense variant in *SGLT4* (rs61746559) and a reduced risk of hypertension.

In modeling binary traits, one usually assumes a liability threshold model, in which an individual develops the disease once they pass a certain threshold on a continuous, quantitative trait (the liability) that follows a normal distribution in the population.[71] Under this model, it is possible that a variant may have an additive effect on the underlying liability but a recessive effect on disease status. For example, it may be that rs66812091 in *PNPLA3* has an additive effect on liver enzyme levels, leading to the accumulation of fatty acids that results in hepatic inflammation (i.e., the underlying quantitative trait), but a recessive effect on NAFLD ($p = 2.4 \times 10^{-12}$). Supporting this, Barton et al.[72] showed in the UK Biobank that variants with known recessive associations to disease can have milder heterozygous effects in related quantitative traits. For our recessive findings, we searched for additive associations to relevant quantitative traits in Jacobs et al.,[73] where additive GWASs on 42 blood-based quantitative traits had been performed in G&H. We found that out of the 54 loci that we expected to show a quantitative trait association, 19 loci, or 35% of the findings, indeed had relevant significant additive quantitative-trait associations (Note S10). Therefore, characterizing the underlying inheritance pattern in greater detail could improve our understanding of disease pathophysiology and highlight homozygous individuals as a higher-risk group to target during screening.

By extension, the patterns of association between a variant and a disease may be context specific and may

differ across ancestries. These complex traits are multifactorial, and differing disease pathophysiologies and environmental effects result in different effect sizes[74] and may affect inheritance patterns as well. This might contribute to the differences in heritability across ancestries[75] and the difficulty in transferring polygenic risk scores to other ancestries.[17] This further emphasizes the importance of performing genetic analyses in diverse cohorts, such as G&H.[76]

With G&H, we were able to perform imputation with a within-cohort reference panel and demonstrated the advantage of using this to improve sensitivity, as previously reported by Barton et al.[15] We saw a subset of recessive findings with p values that were only significant with the WES5Kimputation and not the TOPMEDimputation. We hypothesize from our evaluation of imputation accuracy (Note S4) that this might be due to more accurate imputation of these variants with the within-population reference panel. However, the TOPMEDimputation provided a significantly larger set of variants to work with, which probably explains why most of our associations are from that panel.

Our recessive testing highlights the value of detecting novel recessive associations that would have been missed under the additive model. For example, the rs61746559 missense variant affecting *SGLT4* was previously reported to be additively associated with renin levels, and together with our novel finding that the homozygous genotype is protective for hypertension, it may provide a basis for considering the gene as a drug target. The majority of the GWS findings (152/185) were not GWS under the additive model, illustrating the importance of applying the recessive model of testing to find recessive effects. For example, we showed that an intronic variant (chr13: 109179501G>C, rs2038707) in myosin 16 (*MYO16*), a gene involved in the musculoskeletal system,[77,78] had a recessive association with the ICD10 code M85, "other disorders of bone density and structure" (OR = 3.2, $p = 2 \times 10^{-8}$, dominance deviation $p = 10^{-5}$). It would likely have been missed under the additive model (OR = 1.3, $p = 9 \times 10^{-3}$). This example also illustrates the value of applying recessive testing to a cohort enriched for homozygosity. The AF for this variant in G&H is 0.17, which is similar to the AF in non-Finnish Europeans (NFE) of 0.16.[79] The NFE population has low levels of consanguinity; therefore, at a sample size of 44,000, one would expect, on the basis of the HWE, that the number of homozygous individuals would be ~1,126 and thus that the power to perform this recessive test in this population would be 57%. As a result of increased autozygosity, the observed number of homozygotes in G&H is 1,408, giving 82% power. Therefore, it is possible that additive tests in larger non-consanguineous cohorts may have missed this recessive association. An exome-based study of 394,841 UK 3Biobank individuals and 4,529 phenotypes detected a nominally significant gene-based additive association between putative loss-of-function variants in *MYO16* with the same ICD10 code (OR = 1.1, $p = 5 \times 10^{-4}$).[80] Furthermore, additive tests with FinnGen (release 6) report nominally significant associations between our lead variant and related phenotypes such as fibroblastic disorders (OR = 1.1, $p = 10^{-3}$) and benign neoplasms in the scapula and long bones of the upper limb (OR = 0.7, $p = 10^{-3}$; FinnGen release 6 dataset).

Another study within Genes & Health, Malawsky et al.,[18] demonstrated that increased homozygosity (higher F_{ROH}) was associated with multiple common diseases. The primary hypothesis for these associations is that homozygous regions of the genome contained causal variants with recessive effects on these phenotypes.[81] From the meta-analysis of highly consanguineous subsets of Genes & Health and UK Biobank in that study, F_{ROH} was found to be associated with 12 ICD10 subchapters (passing the FDR 5% multiple testing threshold). Five of the 12 (42%) phenotypes significantly associated with F_{ROH} had underlying single-variant recessive associations reported in this study, compared to 13 of the remaining 49 (27%) phenotypes tested in that paper, which were not significantly associated with F_{ROH} (Fisher's exact test $p = 0.31$). Manually relaxing the phenotype matching for the 12 phenotypes associated with F_{ROH}, we found variants with significant ($p < 5 \times 10^{-8}$) recessive associations to three more closely related phenotypes (Table S10), although none passed our more stringent Bonferroni correction threshold. This supports the hypothesis that the association between increased autozygosity and the prevalence of some common diseases is due to underlying genetic variants with recessive effects.

There are several limitations to the project. Firstly, we have not carried out fine-mapping of these recessive associations because, to our knowledge, there are no established methods for fine-mapping that would help disentangle, for example, a recessive hit that is in LD with a strongly additive hit. However, it is worth noting also that LD diminishes with distance between non-additive variants at a rate that is squared of the rate between additive variants,[1] which should, in theory, make it easier to pinpoint causal recessive variants due to the lower LD. Secondly, there is limited replication of findings with p values slightly below the GWS threshold. This might be because this p value cutoff is not stringent enough, given the number of tests we performed. Thirdly, our power calculations have various limitations. They were performed on a simple logistic regression model, so may have overestimated the power of the model fitted by SAIGE and REGENIE. Furthermore, effect sizes used in the power calculation might be overestimated in the discovery GWAS (winner's curse), which could also lead to overestimated power. Additionally, differences in the granularity of disease classification and differing methods for phenotype curation are not accounted for in the power calculation. For example, there is no thalassemia phenotype available for testing in FinnGen (or GERA), and the G&H phenotypes of "thalassemia" and "other hereditary hemolytic anemia" were matched to "other anemia" and "hemolytic anemia" in FinnGen, which may have contributed to

the poorer replication in that cluster of loci compared to the other Bonferroni-significant loci. Lastly, the Finnish cohort and the GERA cohort are composed of very different ancestries from British South Asians, and the differences in LD patterns between populations could be significant. In particular, the relatively poor replication of Bonferroni-significant variants associated with thalassemia and hereditary anemias in FinnGen might be because selection for resistance to malaria in South Asia has produced complex LD patterns in that genomic region[82,83] that differ from the LD patterns in European-ancestry samples; these are not accounted for in our power calculation. Finally, another limitation is that we only carried out single-variant tests. In the future, exome sequencing of the full G&H cohort will allow us to identify rare variants that can be aggregated within a gene to try to boost the power to find genes with recessive effects and reduce the need for fine-mapping.

In conclusion, with our whole-exome and whole-genome imputation sets, we profiled the recessive landscape at single variants in this cohort of 44,000 British South Asians across a broad spectrum of clinical phenotypes and identified 185 recessive associations. It is likely that many recessive findings remain to be found, and this project provides a sound argument for expanding the search to other cohorts and phenotypes.

Consortia

The consortium members that make up the G&H Research Team are Ahsan Khan, Amna Asif, Ana Angel, Annum Salman, Asma Qureshi, Benjamin M Jacobs, Bill Newman, Caroline Morton, Caroline Winckley, Ceri Durham, Chris Griffiths, Claudia Langenberg, Dan Mason, Daniel MacArthur, Daniel Stow, David A Van Heel, David Collier, Eamonn Maher, Elizabeth Owor, Emily Mantle, Fabiola Eto, Georgios Kalantzis, Gerome Breen, Grainne Colligan, Hanifa Khatun, Hilary Martin, Iaroslav Popov, Ishevanhu Zengeya, Jessry Russell, Joanne Harvey, John Solly, John Wright, Joseph Gafton, Julia Zöllner, Kamrul Islam, Karen A Hunt, Karen Tricker, Klaudia Walter, Matt Hurles, Michael Simpson, Miriam Samuel, Mohammed Bodrul Mazid, Moneeza K Siddiqui, Nishat Safa, Omar Asgar, Panos Deloukas, Raymond Chung, Richard C Trembath, Rohini Mathur, Sabina Yasmin, Saeed Bidi, Sam Hodgson, Samina Ashraf, Sang Hyuck Lee, Sarah Finer, Shaheen Akhtar, Shabana Chaudhary, Shapna Hussain, Sheik Dowlut, Stuart Rison, Teng Heng, Vladimir Ovchinnikov, Vivek Iyer, and Jan Whalley. https://docs.google.com/spreadsheets/d/1D9HLbc_m0KdOUN-gS0hymTLewJ36e8ETSEb8Tu_CqWY/edit?gid=0#gid=0.

Acknowledgments

The acknowledgments are provided in the supplemental information.

Author contributions

T.H.H. helped plan the project, performed the data analysis, and drafted the manuscript. K.W. supervised the association testing. Q.Q.H. supervised the QC and, with D.A.v.H., performed the TOPMED-r2 imputation. J.K. and M.J.D. ran the recessive testing in FinnGen. H.H. advised on FinnGen data and recessive association testing. D.M. contributed to the power and F_{ROH} calculations. G.K. advised on imputation and association testing. D.A.v.H. supervised the collection and curation of G&H data and advised on association testing. H.C.M. planned and led the project and helped draft the manuscript. All authors reviewed the manuscript.

Declaration of interests

The authors declare no competing interests.

Supplemental information

Supplemental information can be found online at https://doi.org/10.1016/j.ajhg.2025.03.020.

Web resources

FinnGen, https://r6.finngen.fi/
Genes & Health, https://www.genesandhealth.org/researchers/
UK Biobank, http://www.nealelab.is/uk-biobank/

Received: April 3, 2024
Accepted: March 31, 2025
Published: April 29, 2025

References

1. Palmer, D.S., Zhou, W., Abbott, L., Wigdor, E.M., Baya, N., Churchhouse, C., Seed, C., Poterba, T., King, D., Kanai, M., et al. (2023). Analysis of genetic dominance in the UK Biobank. Science *379*, 1341–1348.
2. Gebreyesus, G., Sahana, G., Christian Sørensen, A., Lund, M.S., and Su, G. (2020). Novel approach to incorporate information about recessive lethal genes increases the accuracy of genomic prediction for mortality traits. Heredity *125*, 155–166.
3. O'Connor, M.J., Schroeder, P., Huerta-Chagoya, A., Cortés-Sánchez, P., Bonàs-Guarch, S., Guindo-Martínez, M., Cole, J.B., Kaur, V., Torrents, D., Veerapen, K., et al. (2022). Recessive Genome-Wide Meta-analysis Illuminates Genetic Architecture of Type 2 Diabetes. Diabetes *71*, 554–565.
4. Hinney, A., Vogel, C.I.G., and Hebebrand, J. (2010). From monogenic to polygenic obesity: recent advances. Eur. Child Adolesc. Psychiatry *19*, 297–310.
5. Lewis, C.M. (2002). Genetic association studies: design, analysis and interpretation. Brief. Bioinform. *3*, 146–153.
6. Klein, R.J., Zeiss, C., Chew, E.Y., Tsai, J.-Y., Sackler, R.S., Haynes, C., Henning, A.K., SanGiovanni, J.P., Mane, S.M., Mayne, S.T., et al. (2005). Complement factor H polymorphism in age-related macular degeneration. Science *308*, 385–389.
7. Visscher, P.M., Wray, N.R., Zhang, Q., Sklar, P., McCarthy, M.I., Brown, M.A., and Yang, J. (2017). 10 years of GWAS discovery: biology, function, and translation. Am. J. Hum. Genet. *101*, 5–22.

8. Heyne, H.O., Karjalainen, J., Karczewski, K.J., Lemmelä, S.M., Zhou, W., Palotie, A., Daly, M.J., FinnGen, Havulinna, A.S., Kurki, M., and Rehm, H.L. (2023). Mono- and biallelic variant effects on disease at biobank scale. Nature *613*, 519–525.
9. Guindo-Martínez, M., Amela, R., Bonàs-Guarch, S., Puiggròs, M., Salvoro, C., Miguel-Escalada, I., Carey, C.E., Cole, J.B., Rüeger, S., Atkinson, E., et al. (2021). The impact of non-additive genetic associations on age-related complex diseases. Nat. Commun. *12*, 2436.
10. Bycroft, C., Freeman, C., Petkova, D., Band, G., Elliott, L.T., Sharp, K., Motyer, A., Vukcevic, D., Delaneau, O., O'Connell, J., et al. (2018). The UK Biobank resource with deep phenotyping and genomic data. Nature *562*, 203–209.
11. Kurki, M.I., Karjalainen, J., Palta, P., Sipilä, T.P., Kristiansson, K., Donner, K.M., Reeve, M.P., Laivuori, H., Aavikko, M., Kaunisto, M.A., et al. (2023). FinnGen provides genetic insights from a well-phenotyped isolated population. Nature *613*, 508–518.
12. Finer, S., Martin, H.C., Khan, A., Hunt, K.A., MacLaughlin, B., Ahmed, Z., Ashcroft, R., Durham, C., MacArthur, D.G., McCarthy, M.I., et al. (2020). Cohort Profile: East London Genes & Health (ELGH), a community-based population genomics and health study in British Bangladeshi and British Pakistani people. Int. J. Epidemiol. *49*, 20–21i. https://doi.org/10.1093/ije/dyz174.
13. Malawsky, D.S. (2021). Population Structure, Consanguinity Patterns, and Clinical Impact of Homozygosity in British Bangladeshis and Pakistanis (Master's thesis. Apollo - University of Cambridge Repository).
14. Arciero, E., Dogra, S.A., Malawsky, D.S., Mezzavilla, M., Tsismentzoglou, T., Huang, Q.Q., Hunt, K.A., Mason, D., Sharif, S.M., van Heel, D.A., et al. (2021). Fine-scale population structure and demographic history of British Pakistanis. Nat. Commun. *12*, 7189.
15. Barton, A.R., Sherman, M.A., Mukamel, R.E., and Loh, P.-R. (2021). Whole-exome imputation within UK Biobank powers rare coding variant association and fine-mapping analyses. Nat. Genet. *53*, 1260–1269.
16. Taliun, D., Harris, D.N., Kessler, M.D., Carlson, J., Szpiech, Z.A., Torres, R., Taliun, S.A.G., Corvelo, A., Gogarten, S.M., Kang, H.M., et al. (2021). Sequencing of 53,831 diverse genomes from the NHLBI TOPMed Program. Nature *590*, 290–299.
17. Huang, Q.Q., Sallah, N., Dunca, D., Trivedi, B., Hunt, K.A., Hodgson, S., Lambert, S.A., Arciero, E., Wright, J., Griffiths, C., et al. (2022). Transferability of genetic loci and polygenic scores for cardiometabolic traits in British Pakistani and Bangladeshi individuals. Nat. Commun. *13*, 4664.
18. Malawsky, D.S., van Walree, E., Jacobs, B.M., Heng, T.H., Huang, Q.Q., Sabir, A.H., Rahman, S., Sharif, S.M., Khan, A., Mirkov, M.U., et al. (2023). Influence of autozygosity on common disease risk across the phenotypic spectrum. Cell *186*, 4514–4527.e14.
19. Chang, C.C., Chow, C.C., Tellier, L.C., Vattikuti, S., Purcell, S.M., and Lee, J.J. (2015). Second-generation PLINK: rising to the challenge of larger and richer datasets. GigaScience *4*, 7.
20. Loh, P.-R., Danecek, P., Palamara, P.F., Fuchsberger, C., A Reshef, Y., K Finucane, H., Schoenherr, S., Forer, L., McCarthy, S., Abecasis, G.R., et al. (2016). Reference-based phasing using the Haplotype Reference Consortium panel. Nat. Genet. *48*, 1443–1448.
21. Danecek, P., Bonfield, J.K., Liddle, J., Marshall, J., Ohan, V., Pollard, M.O., Whitwham, A., Keane, T., McCarthy, S.A., Davies, R.M., and Li, H. (2021). Twelve years of SAMtools and BCFtools. GigaScience *10*, giab008. https://doi.org/10.1093/gigascience/giab008.
22. Das, S., Forer, L., Schönherr, S., Sidore, C., Locke, A.E., Kwong, A., Vrieze, S.I., Chew, E.Y., Levy, S., McGue, M., et al. (2016). Next-generation genotype imputation service and methods. Nat. Genet. *48*, 1284–1287.
23. Fuchsberger, C., Abecasis, G.R., and Hinds, D.A. (2015). minimac2: faster genotype imputation. Bioinformatics *31*, 782–784.
24. Mbatchou, J., Barnard, L., Backman, J., Marcketta, A., Kosmicki, J.A., Ziyatdinov, A., Benner, C., O'Dushlaine, C., Barber, M., Boutkov, B., et al. (2021). Computationally efficient whole-genome regression for quantitative and binary traits. Nat. Genet. *53*, 1097–1103.
25. Moore, C.M., Jacobson, S.A., and Fingerlin, T.E. (2019). Power and Sample Size Calculations for Genetic Association Studies in the Presence of Genetic Model Misspecification. Hum. Hered. *84*, 256–271.
26. Baulande, S., Lasnier, F., Lucas, M., and Pairault, J. (2001). Adiponutrin, a transmembrane protein corresponding to a novel dietary- and obesity-linked mRNA specifically expressed in the adipose lineage. J. Biol. Chem. *276*, 33336–33344.
27. Xiang, H., Wu, Z., Wang, J., and Wu, T. (2021). Research progress, challenges and perspectives on PNPLA3 and its variants in Liver Diseases. J. Cancer *12*, 5929–5937.
28. Basu Ray, S. (2019). PNPLA3-I148M: a problem of plenty in non-alcoholic fatty liver disease. Adipocyte *8*, 201–208.
29. Pazoki, R., Vujkovic, M., Elliott, J., Evangelou, E., Gill, D., Ghanbari, M., van der Most, P.J., Pinto, R.C., Wielscher, M., Farlik, M., et al. (2021). Genetic analysis in European ancestry individuals identifies 517 loci associated with liver enzymes. Nat. Commun. *12*, 2579.
30. King, C.D., Rios, G.R., Green, M.D., and Tephly, T.R. (2000). UDP-glucuronosyltransferases. Curr. Drug Metab. *1*, 143–161.
31. Seo, J.Y., Lee, J.-E., Chung, G.E., Shin, E., Kwak, M.-S., Yang, J.I., and Yim, J.Y. (2020). A genome-wide association study on liver enzymes in Korean population. PLoS One *15*, e0229374.
32. Dai, X., Wu, C., He, Y., Gui, L., Zhou, L., Guo, H., Yuan, J., Yang, B., Li, J., Deng, Q., et al. (2013). A genome-wide association study for serum bilirubin levels and gene-environment interaction in a Chinese population. Genet. Epidemiol. *37*, 293–300.
33. Scriver, C.R. (2001). The Metabolic & Molecular Bases of Inherited Disease (McGraw-Hill).
34. Crigler, J.F., Jr., and Najjar, V.A. (1952). Congenital familial nonhemolytic jaundice with kernicterus. Pediatrics *10*, 169–180.
35. Ghoussaini, M., Mountjoy, E., Carmona, M., Peat, G., Schmidt, E.M., Hercules, A., Fumis, L., Miranda, A., Carvalho-Silva, D., Buniello, A., et al. (2021). Open Targets Genetics: systematic identification of trait-associated genes using large-scale genetics and functional genomics. Nucleic Acids Res. *49*, D1311–D1320.
36. Sollis, E., Mosaku, A., Abid, A., Buniello, A., Cerezo, M., Gil, L., Groza, T., Güneş, O., Hall, P., Hayhurst, J., et al. (2023). The NHGRI-EBI GWAS Catalog: knowledgebase and deposition resource. Nucleic Acids Res. *51*, D977–D985.

37. Nuinoon, M., Makarasara, W., Mushiroda, T., Setianingsih, I., Wahidiyat, P.A., Sripichai, O., Kumasaka, N., Takahashi, A., Svasti, S., Munkongdee, T., et al. (2010). A genome-wide association identified the common genetic variants influence disease severity in beta0-thalassemia/hemoglobin E. Hum. Genet. *127*, 303–314.
38. Cao, A., and Galanello, R. (2010). Beta-thalassemia. Genet. Med. *12*, 61–76.
39. Lim, W.F., Muniandi, L., George, E., Sathar, J., Teh, L.K., and Lai, M.I. (2015). HbF in HbE/β-thalassemia: A clinical and laboratory correlation. Hematology *20*, 349–353.
40. He, Z., and Russell, J.E. (2002). A human embryonic hemoglobin inhibits Hb S polymerization in vitro and restores a normal phenotype to mouse models of sickle cell disease. Proc. Natl. Acad. Sci. USA *99*, 10635–10640.
41. Russell, J.E., and Liebhaber, S.A. (1998). Reversal of lethal alpha- and beta-thalassemias in mice by expression of human embryonic globins. Blood *92*, 3057–3063.
42. Métais, J.-Y., Doerfler, P.A., Mayuranathan, T., Bauer, D.E., Fowler, S.C., Hsieh, M.M., Katta, V., Keriwala, S., Lazzarotto, C.R., Luk, K., et al. (2019). Genome editing of HBG1 and HBG2 to induce fetal hemoglobin. Blood Adv. *3*, 3379–3392.
43. Bulger, M., Bender, M.A., van Doorninck, J.H., Wertman, B., Farrell, C.M., Felsenfeld, G., Groudine, M., and Hardison, R. (2000). Comparative structural and functional analysis of the olfactory receptor genes flanking the human and mouse beta-globin gene clusters. Proc. Natl. Acad. Sci. USA *97*, 14560–14565.
44. Game, L., Bergounioux, J., Close, J.P., Marzouka, B.E., and Thein, S.L. (2003). A novel deletion causing (epsilon gamma delta beta) degrees thalassaemia in a Chilean family. Br. J. Haematol. *123*, 154–159.
45. Brantberg, A., Eik-Nes, S.H., Roberts, N., Fisher, C., and Wood, W.G. (2009). Severe intrauterine anemia: a new form of epsilongammagammadeltabeta thalassemia presenting in utero in a Norwegian family. Haematologica *94*, 1157–1159.
46. Van Ziffle, J., Yang, W., and Chehab, F.F. (2011). Homozygous deletion of six olfactory receptor genes in a subset of individuals with Beta-thalassemia. PLoS One *6*, e17327.
47. Võsa, U., Claringbould, A., Westra, H.-J., Bonder, M.J., Deelen, P., Zeng, B., Kirsten, H., Saha, A., Kreuzhuber, R., Yazar, S., et al. (2021). Large-scale cis- and trans-eQTL analyses identify thousands of genetic loci and polygenic scores that regulate blood gene expression. Nat. Genet. *53*, 1300–1310.
48. Goyette, P., Pai, A., Milos, R., Frosst, P., Tran, P., Chen, Z., Chan, M., and Rozen, R. (1998). Gene structure of human and mouse methylenetetrahydrofolate reductase (MTHFR). Mamm. Genome *9*, 652–656.
49. Fan, Y., Wu, L., and Zhuang, W. (2022). Methylenetetrahydrofolate Reductase Gene rs1801133 and rs1801131 Polymorphisms and Essential Hypertension Risk: A Comprehensive Analysis. Cardiovasc. Ther. *2022*, 2144443.
50. Liew, S.-C., and Gupta, E.D. (2015). Methylenetetrahydrofolate reductase (MTHFR) C677T polymorphism: epidemiology, metabolism and the associated diseases. Eur. J. Med. Genet. *58*, 1–10.
51. Frosst, P., Blom, H.J., Milos, R., Goyette, P., Sheppard, C.A., Matthews, R.G., Boers, G.J., den Heijer, M., Kluijtmans, L.A., and van den Heuvel, L.P. (1995). A candidate genetic risk factor for vascular disease: a common mutation in methylenetetrahydrofolate reductase. Nat. Genet. *10*, 111–113.
52. Hustad, S., Midttun, Ø., Schneede, J., Vollset, S.E., Grotmol, T., and Ueland, P.M. (2007). The methylenetetrahydrofolate reductase 677C-T polymorphism as a modulator of a B vitamin network with major effects on homocysteine metabolism. Am. J. Hum. Genet. *80*, 846–855.
53. Kang, S.S., Zhou, J., Wong, P.W., Kowalisyn, J., and Strokosch, G. (1988). Intermediate homocysteinemia: a thermolabile variant of methylenetetrahydrofolate reductase. Am. J. Hum. Genet. *43*, 414–421.
54. Guttormsen, A.B., Ueland, P.M., Nesthus, I., Nygård, O., Schneede, J., Vollset, S.E., and Refsum, H. (11 1996). Determinants and vitamin responsiveness of intermediate hyperhomocysteinemia (> or = 40 micromol/liter). The Hordaland Homocysteine Study. J. Clin. Investig. *98*, 2174–2183.
55. Jacques, P.F., Bostom, A.G., Williams, R.R., Ellison, R.C., Eckfeldt, J.H., Rosenberg, I.H., Selhub, J., and Rozen, R. (1996). Relation between folate status, a common mutation in methylenetetrahydrofolate reductase, and plasma homocysteine concentrations. Circulation *93*, 7–9.
56. Viswanathan, M., Urrutia, R.P., Hudson, K.N., Middleton, J.C., and Kahwati, L.C. (2023). Folic Acid Supplementation to Prevent Neural Tube Defects: Updated Evidence Report and Systematic Review for the US Preventive Services Task Force. JAMA *330*, 460–466.
57. de Franchis, R., Buoninconti, A., Mandato, C., Pepe, A., Sperandeo, M.P., Del Gado, R., Capra, V., Salvaggio, E., Andria, G., and Mastroiacovo, P. (1998). The C677T mutation of the 5,10-methylenetetrahydrofolate reductase gene is a moderate risk factor for spina bifida in Italy. J. Med. Genet. *35*, 1009–1013.
58. Mornet, E., Muller, F., Lenvoisé-Furet, A., Delezoide, A.L., Col, J.Y., Simon-Bouy, B., and Serre, J.L. (1997). Screening of the C677T mutation on the methylenetetrahydrofolate reductase gene in French patients with neural tube defects. Hum. Genet. *100*, 512–514.
59. Ou, C.Y., Stevenson, R.E., Brown, V.K., Schwartz, C.E., Allen, W.P., Khoury, M.J., Rozen, R., Oakley, G.P., Jr., and Adams, M.J., Jr. (1996). 5,10 Methylenetetrahydrofolate reductase genetic polymorphism as a risk factor for neural tube defects. Am. J. Med. Genet. *63*, 610–614.
60. van der Put, N.M., Steegers-Theunissen, R.P., Frosst, P., Trijbels, F.J., Eskes, T.K., van den Heuvel, L.P., Mariman, E.C., den Heyer, M., Rozen, R., and Blom, H.J. (1995). Mutated methylenetetrahydrofolate reductase as a risk factor for spina bifida. Lancet *346*, 1070–1071.
61. Tazawa, S., Yamato, T., Fujikura, H., Hiratochi, M., Itoh, F., Tomae, M., Takemura, Y., Maruyama, H., Sugiyama, T., Wakamatsu, A., et al. (2005). SLC5A9/SGLT4, a new Na+-dependent glucose transporter, is an essential transporter for mannose, 1,5-anhydro-D-glucitol, and fructose. Life Sci. *76*, 1039–1050.
62. Shubrook, J.H., Bokaie, B.B., and Adkins, S.E. (2015). Empagliflozin in the treatment of type 2 diabetes: evidence to date. Drug Des. Devel. Ther. *9*, 5793–5803.
63. Zaccardi, F., Webb, D.R., Htike, Z.Z., Youssef, D., Khunti, K., and Davies, M.J. (2016). Efficacy and safety of sodium-glucose co-transporter-2 inhibitors in type 2 diabetes mellitus: systematic review and network meta-analysis. Diabetes Obes. Metab. *18*, 783–794.
64. Thomas, M.C., and Cherney, D.Z.I. (2018). The actions of SGLT2 inhibitors on metabolism, renal function and blood pressure. Diabetologia *61*, 2098–2107.

65. Mazidi, M., Rezaie, P., Gao, H.-K., and Kengne, A.P. (2017). Effect of Sodium-Glucose Cotransport-2 Inhibitors on Blood Pressure in People With Type 2 Diabetes Mellitus: A Systematic Review and Meta-Analysis of 43 Randomized Control Trials With 22 528 Patients. J. Am. Heart Assoc. *6*, e004007. https://doi.org/10.1161/JAHA.116.004007.
66. Dharia, A., Khan, A., Sridhar, V.S., and Cherney, D.Z.I. (2023). SGLT2 Inhibitors: The Sweet Success for Kidneys. Annu. Rev. Med. *74*, 369–384.
67. Schork, A., Saynisch, J., Vosseler, A., Jaghutriz, B.A., Heyne, N., Peter, A., Häring, H.-U., Stefan, N., Fritsche, A., and Artunc, F. (2019). Effect of SGLT2 inhibitors on body composition, fluid status and renin-angiotensin-aldosterone system in type 2 diabetes: a prospective study using bioimpedance spectroscopy. Cardiovasc. Diabetol. *18*, 46.
68. Gyimesi, G., Pujol-Giménez, J., Kanai, Y., and Hediger, M.A. (2020). Sodium-coupled glucose transport, the SLC5 family, and therapeutically relevant inhibitors: from molecular discovery to clinical application. Pflugers Arch. *472*, 1177–1206.
69. Folkersen, L., Gustafsson, S., Wang, Q., Hansen, D.H., Hedman, Å.K., Schork, A., Page, K., Zhernakova, D.V., Wu, Y., Peters, J., et al. (2020). Genomic and drug target evaluation of 90 cardiovascular proteins in 30,931 individuals. Nat. Metab. *2*, 1135–1148.
70. Laurent, S. (2017). Antihypertensive drugs. Pharmacol. Res. *124*, 116–125.
71. Wright, S. (1934). An Analysis of Variability in Number of Digits in an Inbred Strain of Guinea Pigs. Genetics *19*, 506–536.
72. Barton, A.R., Hujoel, M.L.A., Mukamel, R.E., Sherman, M.A., and Loh, P.-R. (2022). A spectrum of recessiveness among Mendelian disease variants in UK Biobank. Am. J. Hum. Genet. *109*, 1298–1307.
73. Jacobs, B.M., Stow, D., Hodgson, S., Zöllner, J., Samuel, M., Kanoni, S., Bidi, S., Walter, K., Langenberg, C., et al.; Genes & Health Research Team (2024). Genetic architecture of routinely acquired blood tests in a British South Asian cohort. Nat. Commun. *15*, 8929.
74. Wang, J., Zhang, Z., Lu, Z., Mancuso, N., and Gazal, S. (2024). Genes with differential expression across ancestries are enriched in ancestry-specific disease effects likely due to gene-by-environment interactions. Am. J. Hum. Genet. *111*, 2117–2128.
75. Visscher, P.M., Hill, W.G., and Wray, N.R. (2008). Heritability in the genomics era–concepts and misconceptions. Nat. Rev. Genet. *9*, 255–266.
76. Fatumo, S., Chikowore, T., Choudhury, A., Ayub, M., Martin, A.R., and Kuchenbaecker, K. (2022). A roadmap to increase diversity in genomic studies. Nat. Med. *28*, 243–250.
77. Kengyel, A., Bécsi, B., Kónya, Z., Sellers, J.R., Erdődi, F., and Nyitrai, M. (2015). Ankyrin domain of myosin 16 influences motor function and decreases protein phosphatase catalytic activity. Eur. Biophys. J. *44*, 207–218.
78. Chou, C.-H., Wu, C.-C., Song, I.-W., Chuang, H.-P., Lu, L.-S., Chang, J.-H., Kuo, S.-Y., Lee, C.-H., Wu, J.-Y., Chen, Y.-T., et al. (2013). Genome-wide expression profiles of subchondral bone in osteoarthritis. Arthritis Res. Ther. *15*, R190.
79. Chen, S., Francioli, L.C., Goodrich, J.K., Collins, R.L., Kanai, M., Wang, Q., Alföldi, J., Watts, N.A., Vittal, C., Gauthier, L.D., et al. (2024). A genomic mutational constraint map using variation in 76,156 human genomes. Nature *625*, 92–100.
80. Karczewski, K.J., Solomonson, M., Chao, K.R., Goodrich, J.K., Tiao, G., Lu, W., Riley-Gillis, B.M., Tsai, E.A., Kim, H.I., Zheng, X., et al. (2022). Systematic single-variant and gene-based association testing of thousands of phenotypes in 394,841 UK Biobank exomes. Cell Genom. *2*, 100168.
81. Charlesworth, D., and Willis, J.H. (2009). The genetics of inbreeding depression. Nat. Rev. Genet. *10*, 783–796.
82. Kumar, R., Sagar, C., Sharma, D., and Kishor, P. (2015). β-globin genes: mutation hot-spots in the global thalassemia belt. Hemoglobin *39*, 1–8.
83. Hedrick, P.W. (2012). Resistance to malaria in humans: the impact of strong, recent selection. Malar. J. *11*, 349.

The American Journal of Human Genetics, Volume *112*

Supplemental information

Widespread recessive effects on common diseases in a cohort of 44,000 British Pakistanis and Bangladeshis with high autozygosity

Teng Hiang Heng, Klaudia Walter, Qin Qin Huang, Juha Karjalainen, Mark J. Daly, Henrike O. Heyne, FinnGen, Daniel S. Malawsky, Georgios Kalantzis, Genes & Health Research Team, Sarah Finer, David A. van Heel, and Hilary C. Martin

Supplemental notes

Note S1: The statistical power needed for recessive analyses

We ran simulations to show that power to detect a recessive effect can be boosted firstly by fitting a recessive rather than an additive model, and secondly by increased homozygosity in the cohort.

We used the genpwr R package [1] for the simulations. We applied the following parameters for all calculations: sample size = 44,000 (mimicking G&H), p-value threshold = $p<5x10^{-8}$, model = recessive (or additive), regression = logistic, and assumed there was no gene-environment interaction. The power, allele frequency (AF), case rate and odds ratio (OR) were modified depending on what was being simulated. Since the package does not accept genotype frequencies, but rather, takes the AF as input and assumes the Hardy-Weinberg Equilibrium (HWE) to determine the genotype frequencies, to account for increased autozygosity in G&H, we calculated the frequency at which one would expect to see the number of homozygotes that we would actually see under HWE, denoted below as "$AF_{autozyg}$". Specifically, we calculated:

$$AF_{autozyg} = \sqrt{(1 - F) * AF_{out}^2 + F * AF_{out}}$$

AF_{out}: $AF\ of\ the\ variant\ in\ a\ hypothetical\ population\ at\ HWE$

$F = The\ average\ inbreeding\ coefficient\ in\ the\ sample$

The parameter F (commonly known in the scientific literature as the “inbreeding coefficient”) corresponds to the average relatedness of parents of individuals in the sample with each other. It also corresponds to the average fraction of the genome homozygous in a given sample. [2]

We assessed if we would have power to detect recessive findings with similar ORs, AFs and case rates as those detected in FinnGen. The FinnGen recessive hits were collated from Table 1 of Heyne et al. (2023) [3]. We filtered the thirty-one findings to the eighteen findings (Table S3) that were validated in release 6 [4]. The ORs, AFs and case rates were then projected into the power calculations we performed.

Using an OR of 2 and a phenotype case frequency of 1%, we calculated the power of a recessive versus an additive test across the AF spectrum, simulating various levels of average autozygosity in the cohort, ranging from a cohort with no autozygosity (F = 0), to a consanguineous cohort with an average inbreeding coefficient of 10% (which corresponds to the average fraction of individuals homozygous at any position in the genome). In practice, the average fraction of the genome homozygous in individuals from G&H is ~2.2%. We first see that the additive model had less power for detecting a truly recessive effect than the recessive model, particularly at lower AFs (Figure S1A,B). Next, we see that power to detect this recessive effect increased with the average level of autozygosity, and for this set of parameters, this was most noticeable around the AF 0.2 - 0.5 range (Figure S1A). This sample size and levels of consanguinity simulated were not powered to detect an effect size of OR = 2 in rare variants, therefore we increased the OR to 5 and reran the simulations for

the rare and low frequency spectrum, and we still saw that power is higher with increasing F (Figure S1B).

We next wanted to evaluate the minimum OR we would be powered (80% power) to detect in G&H. To do that we simulated varying case rates from 0.1% to 25%. We projected the recessive findings reported in FinnGen by Heyne et al. (2023) onto our simulations, and found that the majority of the findings had ORs higher than the minimum that we are powered to detect at their corresponding case rates and AFs (Figure S1C). This suggested that G&H will be well powered to detect very large ORs at rare variants i.e. effectively Mendelian associations, and smaller recessive effects at common variants with common traits.

Note S2: QC of genetic data

Within this cohort, exome sequencing was performed using Agilent V5 capture kits on a subset of 5,236 G&H individuals who self-declared as having related parents following the protocol described in Narasimhan et al. (2016) (Agilent SureSelect Human All Exon V5, [5]). Sequencing was performed in batches, resulting in a bimodal distribution of read depth, with some individuals being sequenced to ~40X, and the rest ~20X (Figure S7A). Mapping was performed with the Burrow-Wheeler Aligner (specifically the BWA-MEM algorithm) [6]. Variants were then called using the GATK HaplotypeCaller [7] and annotated with Ensembl Variant Effect Predictor v95 [8]. This was released on September 2019: (https://www.genesandhealth.org/research/scientific-data-downloads/sept-2019-summary-files-exome-sequencing-loss-function-variant)

Subsequently, QC of the WES data relevant to this project was performed, as summarised in Table S1 below.

Note S3: Genetic inference of ancestry and analysis of relatedness

Ancestry inference

We genetically inferred ancestry for the 44,396 individuals by merging the SNP-array data with reference cohorts.

From the SNP-array had been through initial QC, we filtered to variants that were autosomal, common (defined as a minor allele frequency, MAF>0.01), had a call rate of ≥99%, and passed the HWE exact test ($p>10^{-6}$) in self-declared Bangladeshi individuals (Table S1). This filtered dataset was then merged with reference sequences of 3,433 individuals from the 1000 Genome Project (1000G) [9] and Central and South Asian individuals from the Human Genome Diversity Project (HGDP) [10]. We excluded palindromic variants, and variants with significant AF differences between G&H and 676 reference South Asians curated from 1000G and HGDP (since these represented likely genotyping errors). We defined variants with significant AF differences in the following manner: Firstly, we calculated the residuals from the linear regression between the AFs in both datasets. Next, we binned the variants into bins by frequency (in intervals of 0.01), and selected variants for which the residual was >5 standard deviations (5SD) away from the mean of the residuals in that frequency bin. (This choice seemed reasonable after testing various SD thresholds, Figure S3A). Lastly, we performed Fisher's exact tests to compare the genotype counts between the G&H data and the 676 reference South Asians at the variants selected above and excluded those with a $p<10^{-5}$. (Again,

we tested p-value thresholds ranging from 0.05 to a multiple testing correction of <0.05/349,632 variants, and felt that 10^{-5} was reasonable, Figure S3B). The various thresholds and the distribution of the outlier variants excluded are graphically represented in Figure S3C-D.

After merging of G&H with the reference samples, LD pruning was performed (window size 1000 kilobases (kb), step size 50, LD r^2 0.1) with PLINK1.9 and long LD regions were excluded. [11]

Principal component analysis (PCA) was performed with PLINK1.9 on the reference individuals, then the G&H individuals were projected into the reference PC space (PCA1). We calculated uniform manifold approximation and projection (UMAP) coordinates (umap R package) [12]. We found that the UMAP with 7 PCs was optimal to separate the reference individuals into superpopulations. 44,320 out of 44,396 G&H individuals were inferred to be South Asian at this stage (Figure S4A), and carried forward for the downstream analysis.

The PropIBD algorithm in KING [13] was run to estimate pairwise relationships up to fourth degree within G&H, and we removed a minimal set of 14,727 individuals who had at least one relative (3rd degree and closer) in the dataset, leaving 29,668 unrelated individuals.

We performed a second PCA (PCA2) on the unrelated G&H individuals, projecting the related G&H individuals who we had inferred to be South Asian into the PC space. The UMAP with 4 PCs identified distinct clusters that

corresponded well to self-declared Bangladeshi/Pakistani ancestry, and this was used to genetically classify individuals as genetically Bangladeshi or Pakistani (Figure S4B).

Relatedness

Next, we attempted to estimate the number of discrete families from the KING relationship inference (derived from the pairs defined in the '.kin0' output of the command KING –related –degree 3 (using PropIBD)). We identified 35,500 pairs involving 25,920 unique individuals that are inferred to be third degree relatives or closer: 7 identical twin pairs, 6,595 parent-offspring pairs, 5,863 full-sibling pairs, 11,639 second-degree pairs, and 11,396 third-degree pairs. From this, we estimated that these relationships cluster into 5,742 discrete families, with an additional 18,270 individuals with no inferred relationship (3rd degree or closer) to others in the cohort. Given the difficulty in accurately inferring more distant relationships in this endogamous population, we also inferred families considering only first degree relatives, namely parent-offspring and full-sibling pairs. In the cohort, 16,122 individuals have at least one first degree relative, and they were grouped into 6,122 families.

To investigate whether homozygotes are clustered within the same families, we focused on 52 rare variants (MAF <1%) with recessive associations and took all pairs of individuals who carry the same homozygous rare variant. Among the 848 pairs of such individuals, two were inferred as parent-offspring pairs, nine as full siblings, and three as 2nd degree relatives, while the remaining pairs were more distantly related than 3rd degree. In contrast, randomly-selected pairs are less likely to be related, within only one 2nd degree relative observed

in 100 simulations of 848 random pairs. This suggests that, as one would expect, homozygous carriers of rare variants that showed significant recessive associations are indeed more likely to be from the same family, especially as full siblings (who share a quarter of their genome IBD2), compared to randomly selected individuals. However, the majority of these homozygous carriers (98%) are more distantly related than 3rd degree, and we believe that the mixed-effect models implemented in REGENIE should sufficiently account for relatedness in association testing. Indeed, when we reran the association analysis in unrelated individuals (i.e. all pairwise relationships are more distant than 3rd degree), we found good correlation between the -log10p values from the full cohort and the unrelated subset (n = 26,579 in the largest set), though unsurprisingly there was less power (linear regression slope = 0.6, r2 = 0.93, Figure S5).

Note S4: Assessing genotyping and imputation accuracies with concordance analyses

Before merging the SNP-array and WES data to build the reference panel, we evaluated concordance of genotypes between them at overlapping sites. Furthermore, after imputation, we assessed imputation accuracy by comparing imputed genotypes to sequenced genotypes.

Evaluating concordance

Concordance was primarily evaluated by the non-reference discordance rate (NRD) calculated using the following formula:

$$NRD = \frac{xRR + xRA + xAA}{xRR + xRA + xAA + mRA + mAA}$$

$$R : reference\ allele^{*}$$

$$A : alternate\ allele^{*}$$

$$x : nMismatches$$

$$m : nMatches$$

*Note that the ‘truth’ was defined by the array genotype when comparing concordance between array and WES data, and defined by the sequenced genotype when comparing concordance between sequenced and imputed data. For example, “xRR” would mean that the truth dataset had a homozygous

reference genotype while the other dataset did not, and “mRA” means that both datasets had a heterozygous genotype.

When examining NRD stratified by allele frequency, the NRD was modified to calculate a “minor allele discordance” (MAD) rate:

$$MAD = \frac{xMajMaj + xMajMin + xMajMin}{xMajMaj + xMajMin + xMajMin + mMajMin + mMinMin}$$

$$Maj : major\ allele^{*}$$

$$Min : minor\ allele^{*}$$

Where relevant, Pearson correlation r^2 between genotypes / dosages were calculated as a secondary measure of concordance.

Concordance between the SNP-array and the WES data

QC on both the SNP-Array and WES datasets improved the overall NRD from 5.02% to 0.40%, with the WES GQ filter making the biggest difference (Table S2). We anticipated that the genotyping accuracy would be low for rare variants in the SNP-array, but surprisingly the array data continued to demonstrate good MADs of ~0.7-0.9% with the WES for variants with minor allele counts (MACs) on the array of 3-6 (equivalent to MAF 0.03-0.06%, Figure S6). We decided to use variants with MAF>0.1% from the SNP array for the imputation backbone.

Since minimac3 (Which is used to build the reference panel for imputation with minimac4) does not tolerate missingness in the imputation reference panel, we needed to use the WES data without any genotype-level QC to minimise

missingness. To select for variants with a majority of high-quality genotypes, we retained those that had <30% missing genotypes after applying the genotype-level QC. Using the raw genotypes at those sites increased the NRD to 1.26% (Table S2). Note that this value is inflated by the discordance at sites with MAC 0-2 (Figure S6), but variants this rare were filtered out of the SNP-array when building the imputation backbone, and instead retained in the WES data, since we assume these are likely to be more accurate than the array genotypes given the difficulties of genotyping uber-rare variants on arrays [14].

Based on the concordance analysis, we decided to include only array variants with MAF>0.1% in the imputation backbone. Overlapping positions with the WES were resolved as follows:

- For SNPs at overlapping positions with matched alleles and with MAF>0.1%, we retained these in the GSA data but removed them from the WES.
- We excluded overlapping common palindromic variants (MAF>0.4) where the strand could not be confidently determined.
- We excluded overlapping positions with unmatched alleles (including all indels).

This resulted in 469,678 variants, which were then phased with EAGLE2 (Kpbwt=20,000) [15].

Based on the concordance of the SNP-array and WES, 91 samples that had high missingness and/or high non-reference discordance (NRD) values were

excluded (Figure S7B, Figure S7C), leaving 4,982 samples. Specifically, we excluded individuals who had any of the following:

- a raw WES call rate <4SD from the mean raw WES call rate,
- a post-genotype QC WES call rate <2SD from the mean post-genotype QC WES call rate,
- an NRD based on the post-genotype QC WES and post-QC SNP-array data of >4SD from the mean

To build the reference panel, we filtered the WES data to a subset of high-quality variants that had ≥70% call rate after genotype-level QC, and removed singletons. Since Minimac3 does not cope with missing genotypes, we used the genotypes at those sites from the raw data (i.e. pre-genotype QC), and replaced missing genotypes (~0.02% of the total genotypes, Figure S8A) with 0/0 if the reference allele was the major allele and 1/1 otherwise. The distribution of raw per-variant missingness at sites that pass a post-genotype QC call rate ≥70% is shown in Figure S8B, with most variants having a raw missingness of <1%.

Variants with MAF>0.1% that were also present on the GSA array and had been included in the the imputation backbone were removed from the reference panel, since they tended to have higher call rates in the array data. The cleaned WES and cleaned SNP-array data from the 4,982 samples were then merged to form the reference panel and phased with EAGLE2 (Kpbwt=20,000). The reference panel consisted of 1,385,942 variants.

Evaluating imputation accuracy

After imputation, to assess the imputation accuracy, the imputed genotypes for the 4,982 individuals with WES data were compared to their sequenced genotypes. Genotype concordance was evaluated between the SNP-array and the WES data at overlapping sites, and between the sequenced and imputed variants.

Ten trials of imputation were performed using the WES5K panel, with each trial leaving 10% of the WES samples out of the reference panel against which we then evaluated concordance between the sequenced and imputed genotypes.

The total number of variants in each imputed dataset ranged from 1,325,855-1,327,697 across the 10 trials. Although the genotyped backbone is imputed by Minimac4 as well, to evaluate imputation accuracy purely at positions with no prior information, we excluded backbone SNPs resulting in 855,697-857,537 variants to compare in each trial. The overall NRD of the imputed genotypes compared to the sequenced genotypes ranged from 7.44-7.69% across 10 trials (Figure S9A).

The confidence of the imputation is quantified by Minimac4's imputed R^2. By convention, a minimum cutoff of ≥0.3 is applied to QC imputed data, which should sufficiently filter out poorly-imputed variants based on the distribution of imputed R^2 scores (Figure S9B). However the overall NRD was not found to improve at this cutoff (Figure S9A) (probably because we are including so many rare variants) so we applied a more stringent cutoff of ≥0.5. As expected, the rarer MAF bins had more variants with a lower imputed R^2 (Figure S9C).

MAD also increases with decreasing allele frequencies, as expected. Many of these extremely rare variants will not be submitted for association testing as they are both poorly imputed and not powered enough for recessive tests.

After applying imputed R^2 ≥0.5 and number of homozygotes (N_{Hom}) ≥3 to prepare for association tests, and including the positions of the SNP-array backbone (which will be included in association tests), the overall NRD improved to 1.78-1.84% across the trials (Figure S10).

The TOPMEDimputation genotypes of the 4,982 samples with WES were compared to their sequenced genotypes. The same cutoffs (imputed R^2 ≥0.5 and N_{Hom} ≥3) were applied to result in 10,045,406 variants. Of these variants, 523,018 variants overlapped with the WES and at these, the overall NRD was 1.19%.

We then considered NRD at variants stratified by the number of homozygotes as defined in the WES5Kimputation, the rationale being that the WES should be considered “truth” in this case, and that the power of the recessive association testing depends on the number of homozygotes rather than directly on the allele frequency. First, we observed that the within-cohort WES5K reference panel allowed for more variants to be imputed at the chosen level of accuracy, especially at lower MAFs (Table S3). Second, for N_{Hom} ≥9 up to a homozygous frequency of 5%, the MAD is lower for the WES5KImputation compared to the TOPMEDImputation, but the opposite is true for the lowest and highest MAF bins. Thirdly, the lower overall NRD of the TOPMEDimputation (1.19% versus ~1.7% for the WES5K Panel) is driven by the improved accuracy of common

variant imputation with the TOPMED reference panel. (Figure S10) As both imputation sets carried their own strengths and weaknesses across the frequency spectrum, it was decided to bring both sets forward to association testing.

Note S5: On multiple testing and the independence of phenotypes

The 898 phenotypes tested are not completely independent of each other, and there may be significant correlation between phenotypes, particularly as some diseases are repeated in the custom list and the ICD10 codes. We sought to calculate Pearson correlation r^2 between phenotype pairs to quantify the degree of correlation in our phenotypic data. This serves two purposes - to evaluate if phenotypes we would expect to be highly correlated indeed have a high r^2 (meaning that they have been curated correctly), and secondly, to try to eliminate highly-correlated phenotypes to reduce the multiple-testing burden.

Of the 402,753 pairs of phenotypes generated, 153 pairs had a correlation r^2 of ≥ 0.5. Manually inspecting these pairs, the majority were between conditions one would expect to be highly correlated; for example, the ICD10 encoding for sarcoidosis and for multiple sclerosis fully correlated ($r^2 = 1$) with the respective custom encodings for these conditions. There were also correlations between biologically similar phenotypes, such as pulmonary heart disease and pulmonary hypertension ($r^2 = 0.89$), and correlations between pairs for which one trait was a subset of the other, such as acute pancreatitis and pancreatitis ($r^2 = 0.90$). By reviewing these highly-correlated pairs, we estimated that only about 80-90 phenotypes could be excluded due to being highly correlated, as the rest of the pairs had differences in their definitions that warranted the inclusion of both phenotypes.

We therefore tested all 898 phenotypes available, and to be stringent, the Bonferonni cutoff we used accounted for all tests as if they were independent.

The number of phenotypes, individuals, and variants tested is summarised in Table S4. A small minority of tests failed on REGENIE; a total of 9,197,933,046 tests produced a p-value, out of a possible 9,374,352,233 tests (98% success rate).

Note S6: On covariates included in the association testing

The covariates included were age (at year of phenotype curation, 2022), sex, age^2, age x sex, age^2 x sex and the first ten principal components (PCs) from the principal component analysis on unrelated G&H individuals described above.

We controlled for ten genetic PCs derived from common variants. These top ten PCs explained more than 85% of the variance explained by the top 50 PCs (Figure S11A).

For the 56 out of 185 recessive loci that involved rare variants (AF <1%), we further investigated if the common PCs were adequately controlling for population structure by checking that the results held when additionally controlling for PCs based on an IBD sharing matrix that should capture more subtle, recent population structure. We used KING to call IBD segments and calculate the length of IBD sharing across pairs of individuals in the cohort, and performed a PCA on the matrix of IBD sharing. We then repeated the association analyses for the 56 recessive loci involving rare variants, adding the first 15 PCs from the IBD PCA to the existing covariates (since these explained most of the variance in the PCA; Figure S11B). Reassuringly, the betas (i.e. the effect sizes, linear regression slope 1.03, r2 0.98, p-value $<2\times10^{-16}$, Figure S11C), and the p-values (converted to -log10p, linear regression slope 0.67, r2 0.25, p-value 5.4×10^{-5}, Figure S11D), of the associations correlated well between the association tests performed controlling for IBD PCs and the

association tests performed controlling for common PCs, with the exception of a single outlier.

Previous work in the lab by [16] demonstrated that increased runs of homozygosity (ROHs) in the genome was associated with several conditions such as anxiety and type 2 diabetes. We sought to explore if the fraction of the genome in ROHs (F_{ROH}) is a possible confounder for these recessive findings, by adding it as a covariate and rerunning the association tests for the 42 lead SNPs in the WES5Kimputation dataset. ROHs were calledby PLINK1.9 on the SNP-array data with the following specifications: maximum inverse density 50kb/SNP, maximum internal gap 1000kb, minimum SNP count 50, maximum 1 heterozygous in scanning window hit, maximum 4 missing calls in scanning window hit and a scanning window sizeof 50. The total length of ROHs (in kb) was then divided by the length of the autosome (approximately 2700000kb) to obtain F_{ROH}. After controlling for F_{ROH}, the recessive p-values correlate well with those obtained without controlling for it, suggesting that this additional covariate is unnecessary (Figure S12).

Note S7: Characteristics of loci identified as significant in the recessive association testing

Information about the 185 lead variants passing $p<5x10^{-8}$ are tabulated in Table S5. The frequency and consequence distributions of the lead variants are shown in Figure S13. Despite applying an exome reference panel including many rare protein-coding variants, many of the findings from the WES5K imputation were still common and intronic, implicating variants near exonic regions that happened to be captured with WES, or common SNPs from the GSA backbone. Similarly, the majority of the significant hits from the whole genome TOPMEDimputation were within the non-coding regions. This is expected as common variants have better power and 99% of the human genome is non-coding.

As an example for plotting and visualisation purposes, we plotted the recessive tests performed between the TOPMEDImputation and D58[Other hereditary haemolytic anaemias]. The Quantile-quantile (QQ) plot (Figure S14A) suggests that the tests are underpowered, as we see substantial deflation of the test statistics below what is expected under the null (lambda = 0.56). When we split the variants contributing to these tests into common (AF > 5%) and low frequency variants (AF ≤ 5%), indeed we see that the lambda of 0.94 for the common variants is close to 1 (Figure S14BC), while the lambda for the rare variants is low at 0.21 (Figure S14C), indicating that the deflation is likely due to reduced power for rare variants.

Next, we tested whether significant recessive associations were more likely to be coding variants than non-coding ones. In our association testing, we evaluated 10,045,406 variants that were imputed with TOPMED, among which 91,930 were coding variants, and identified 1,216 variants with significant recessive associations with any phenotype. We observed significant enrichment of recessive associations amongst coding variants (Chi-square test P-value = 8.3×10^{-20}), with 0.044% having significant associations compared to 0.011% of non-coding variants. As expected, given the coding regions only cover ~1% of the genome, the vast majority of significant recessive effects we detected are non-coding (96.5%). However, the significant recessive associations are enriched among coding variants compared to the total set of variants tested for association: 3.5% of recessive variants are coding while only 0.9% of all tested variants are coding, as shown in Table S6.

To assess whether the enrichment varied by MAF, we stratified the analysis by MAF bins. We observed significant enrichment for coding variants amongst common variants (MAF > 5%; P-value = 2.3×10^{-16}) and low-frequency variants (MAF 1–5%; P-value = 2.4×10^{-8}) but not in rare (MAF 0.1–1%; P-value = 0.66) or ultra-rare variants (MAF < 0.1%; no coding variants reached significance in recessive testing). The enrichment analysis for the rare and ultra-rare groups is likely underpowered, since only 94 and 9 variants, respectively, showed significant recessive associations. These findings are consistent with the expectation that protein-coding variants are more likely to impact gene function and health outcomes, and that they have larger effect sizes leading to better power for detection.

Note S8: Evaluating the dominance deviation of the 185 recessive findings

For the lead variants of the 185 recessive loci, 152 lead variants were not GWS in the additive test. Looking at the distribution of AFs, recessive hits that were GWS in the additive test had higher AFs than hits that were insignificant in the additive test, suggesting that common variants simply had more power to be detected under the additive model even if the underlying pattern of inheritance might be recessive. (Figure S15A).

For the lead variants of the significant recessive associations, we also reran the additive test (step 2 of REGENIE) after removing homozygous individuals, to explore heterozygous effects. When doing this, all but two lead variants had p-values below GWS, demonstrating both the weight of these homozygotes on the significant results in the original additive tests and the loss of power by reducing the sample size of the tests. The remaining two lead variants likely have strong heterozygous effects that can be detected even at reduced power. (Figure S15D)

It is possible that the tests that dropped below GWS after homozygotes were removed may still have heterozygous effects. We compared the betas in the different models of testing. As expected, additive tests estimated betas that were smaller in magnitude compared to the recessive tests (Figure 2B). After dropping the homozygotes, 107 of these tests became insignificant (p-value > 0.05), but for tests that remained nominally significant, their betas correlated well with the full additive tests, though again, their estimated effect sizes were

smaller (Figure S15E). These nominally-significant additive tests performed without homozygous individuals demonstrate heterozygous effects despite reduced power.

When we removed the homozygous individuals for the additive tests, a small minority of tests (18/185 lead variants) could not be run in REGENIE when the homozygotes were excluded. These tests tend to have higher AFs greater than 0.7 (suggesting that the removal of homozygotes resulted in the test being too underpowered) (Figure S15B).

To further explore whether the recessive model was indeed the best fit for the variants that were significant on the recessive test in REGENIE, we performed logistic regression testing in R, using a genotypic model that included a dominance deviation encoding. For comparison, we also fitted a standard additive and recessive model in R. We re-coded the genotypes to perform additive, recessive and genotypic tests with 2 degrees of freedom, as shown in Table S7 and the equations below.

<u>Additive test:</u>

$$phenotype \sim \beta_{add} G_{additive} + BC$$

where $G_{additive}$ is the genotype of the SNP using the additive encoding (0/1/2), β_{add} is the effect size under an additive model, C is a matrix of covariates (defined below) and B is a vector of effect sizes for those covariates.

Recessive test:

$$phenotype \sim \beta_{rec} G_{recessive} + BC$$

where $G_{recessive}$ is the genotype of the SNP using the recessive encoding (0/0/1), and β_{rec} is the effect size under a recessive model.

Genotypic (2 degrees of freedom) test, to extraction dominance deviation:

$$phenotype \sim \beta_{add} G_{additive} + \beta_{domdev} G_{domdev} + BC$$

where G_{domdev} is the genotype of the SNP using the dominance deviation encoding (0/1/0), and β_{domdev} is the effect size of the dominance deviation under the genotypic model.

The tests were performed on the full cohort (i.e. the set of individuals used in REGENIE, sample sizes in Table S4), as well as on the subset of individuals genetically-inferred to be unrelated by KING (26,579 individuals in the largest cohort, subsetted accordingly depending on the imputation and phenotype tested).

Covariates

The covariates included were age, sex, age^2, age x sex, age^2 x sex and the first ten PCs.

In Figure S16, the results from the additive and recessive tests in R were plotted similarly to those in REGENIE in Figure 2 and gave similar conclusions to those noted earlier in the main text.

Before considering the results from the dominance deviation tests, we compared the results from the standard recessive model between R logistic regression and REGENIE. We found that the -log10(p-values) correlated well (Figure S17A, linear regression slope = 1.1, r^2 = 0.96). This correlation still held when restricting to a set of 26,579 unrelated individuals (in the largest set) in R, though unsurprisingly there was less power (linear regression slope = 0.6, r^2 = 0.93). Still, there were some REGENIE tests that were not significant (p-value > 0.05) in the R logistic regression, and these outliers had much larger effect size estimates in R (Figure S17B). Otherwise, for tests that were nominally significant in R, their betas correlated well with REGENIE (linear regression slope = 0.98, r^2 = 0.98). The outlier R tests that had insignificant p-values tended to involve rarer variants (Figure S17C, Wilcoxon two-sided p-value = $2x10^{-4}$), although the distribution of phenotype case counts were similar (Figure S17C, Wilcoxon two-sided p-value = 0.27). They were excluded from subsequent analyses described below.

When fitting the genotypic model, 76% (or 140) of these lead variants had nominally significant dominance deviation p-values. These variants tended to be at least nominally significant in the R recessive test as well (chi-square test p-value = $1.3x10^{-11}$, Figure S17D). For the tests that had an insignificant dominance deviation p-value, we cannot rule out that the underlying inheritance pattern may still be recessive, especially as some also had recessive tests with

insignificant p-values in R, suggesting that R is an imperfect model to replicate the results from the more complex model fitted by REGENIE.

Regardless, as expected, the recessive and dominance deviation p-values were correlated (linear regression of their log10-transformed p-values: slope = 4, r^2 = 0.3, p-value <2.2×10^{-16}) (Figure S17E), with hits having a nominally significant dominance deviation tending to have lower recessive p-values (Wilcoxon two-sided p-value = 2.8×10^{-5}). We also saw that hits having a nominally significant dominance deviation also tended to have a larger difference between their recessive and additive p-values (Wilcoxon two-sided p-value = 3×10^{-9}) (Figure S17F). We did not find any difference in the recessive betas, distribution of AFs and case counts between the hits with nominally significant and insignificant dominance deviation p-values (Wilcoxon two-sided p-value 0.3, 0.7 and 0.2 respectively, Figure S17G-H).

In summary, from removing homozygotes and re-performing the additive tests, we demonstrated that several of our recessive findings may harbour mild heterozygous effects. In addition, fitting the genotypic model in R provided further support for at least three-quarters of our findings being truly recessive.

Note S9: Replication in other cohorts

Table S7 and Table S8 list the Genes & Health phenotypes being matched to FinnGen phenotypes and GERA phenotypes respectively.

The current locus definition (r^2>0.25 and within 1.5Mb of the lead variant) was chosen so as not to inflate the pairs of independent findings we report, but for the purpose of replication in other cohorts, they are not as stringent as the one described in Huang et al. 2022. For completeness, we repeated the replication calculations with the Huang et al. 2022 cutoffs, so as to calculate a PAT in a manner closer to what was described in the literature. For each significant locus in G&H, we first identified proxy variants as variants that are within a +/-50kb window from the lead variant with LD r^2 ≥0.8, and a p-value ≤100 times the p-value of the lead variant. The PAT in FinnGen reduced from 23% to 21% for genome-wide significant loci with this more stringent locus definition. Notably, the PAT for the Bonferroni-significant loci increased from 44% to 56%, perhaps reflecting the complex LD patterns in the chromosome 11 thalassemia and hereditary anaemia findings, and how different locus definitions would therefore affect the counting of the number of independent loci for these associations.

Additionally, we have performed PAT calculations while varying the replication criteria, trying various more stringent cutoffs, such as replication only by the lead variant, and considering a p-value cutoff of ≤0.01 or ≤0.05/number of tests in the replication cohort. We have presented the results of these different trials in Table S10. Firstly, at our original replication p-value threshold of 0.05, the percentage of loci that replicate by their lead variant alone was 53% for

genome-wide significant variants and 57% for Bonferroni significant variants. Next, at a replication p-value cutoff of ≤0.05/number of tests, the PAT for genome-wide-significant loci decreased from 23% to 14%, and the PAT for Bonferroni-significant loci changed to a smaller extent from 44% to 38%. Broadly, stricter definitions for replication do reduce the number of loci we have replicated, and this affects the genome-wide-significant findings more than the Bonferroni-significant findings.

Note S10: Systematically finding literature support for the recessive associations and quantifying the novelty in our findings

We attempted to identify literature support for the 185 recessive loci in our study with a systematic approach. In addition, we aimed to use the findings from this to quantify the novelty in our findings.

Additive analysis in external datasets

Firstly, we searched external, publicly available datasets, which are mostly additive tests. From 8 October 2024 to 21 October 2024, we searched each lead variant on genetics.opentargets.org. The portal compiled associations between variants and phenotypes reported in FinnGen, UK Biobank and the GWAS Catalog. Different datasets applied different p-value thresholds, and the highest p-value reported in the data within this analysis was 0.0049.

We tabulated relevant associations in Note S11, and assigned a "Literature.Evidence.Score" to the evidence found:

- 9 (None): Variant not found
- 0 (None): Variant is found, but no reported associations with related traits
- 1 (Weak): Association with 1 related trait in 1 dataset. FinnGen and UK Biobank are each defined as 1 dataset. The GWAS Catalog is also defined as 1 dataset (With several exceptions and caveats: Evidence in the GWAS catalog can come from different studies. Some GWAS results may have used UK Biobank or FinnGen as well. Therefore the specific

studies contributing to the scoring have been listed in Note S11 and can be further reviewed.)

- 2 (Weak): Association with 1 related trait in more than 1 datasets.
- 3 (Moderate): Associations with more than 1 related traits, in 1 dataset.
- 4 (Moderate): Associations with more than 1 related traits, in 2 datasets.
- 5 (Strong): Associations with more than 1 related traits across all 3 datasets.
- 6 (Strong): At least 1 association with the same trait in any of the 3 datasets (with or without associations with 1 or more related traits across 1 or more datasets).

We acknowledge that there is some subjectivity in deciding whether a trait is related, and whether different entries with different variations on a trait name count as different traits or the same trait. However, this would be a persistent concern with any attempt at phenotype matching.

Of the 185 lead variants, 166 (~90%) could be found in the Open Targets portal. The remaining ~10% of lead variants that were only reported in G&H tended to be rarer (ANOVA across all seven scores p-value 0.02, one-sided Wilcoxon between variants with score 9 compared to the others p-value $4.0x10^{-5}$). This was expected as variants unique to G&H are likely to be rarer, and further supports the value of performing such genetic analyses in diverse cohorts. In addition, among the variants that could be found in external datasets, increasing AF was associated with stronger evidence (linear regression slope 0.036, r^2 0.05, p-value 0.003). This was also expected, as variants with higher

AF would have better power to be significant with the additive model even if the underlying pattern of association is recessive. (Figure S18A)

Of the 166 loci that could be evaluated in external datasets, 35 had strong support in the literature (27 with associations to the same trait, 8 with associations to multiple related traits across multiple datasets), 28 had moderate support (associations to multiple related traits), 35 had weak support (association to a related trait), and 68 had no associations to any related traits. Arguably, all but the 27 loci with reported associations to the same trait (158 loci remaining, 85%) are novel. With a stricter definition of novelty (i.e. if we define a novel locus as a locus with no reported associations with related traits), 87 loci (19 variants not found, 68 variants found but with no reported association with related traits), or ~47% of our findings, can be considered novel. As the FDR5 threshold of $3.7x10^{-8}$ is similar to the genome-wide-significant threshold of $5x10^{-8}$ we have used here, we can assume that these numbers are inflated by ~5% (assuming that all the false positives are amongst the ‘novel’ set), and that an estimated 42-80% of our findings are novel.

Interestingly, there was no difference in the distribution of recessive p-values across the 8 scores (ANOVA of -log10p across all scores p-value 0.87) (Figure S18B). Instead, the difference between the additive p-value and the recessive p-value (delta log10p) was associated with the strength of the evidence; the smaller the delta log10p, the higher the evidence score (linear regression of delta log10p across scores 0-6 slope -0.32, r^2 0.09, p-value $5.4x10^{-5}$) (Figure S18C). This implied that it was not the magnitude of the recessive p-value that was suggestive of whether the association had been detected before in additive

testing, but the difference in the p-values between the additive and recessive test that was more predictive. This makes sense, as it is possible that many of the recessive associations we report here were missed by other studies that performed additive testing alone, and in cohorts that did not have sufficient homozygous individuals to power recessive analysis.

Additive analysis of quantitative traits in G&H

Next, we also searched the summary statistics of the quantitative traits GWAS performed in G&H [17], which we downloaded on 8 October 2024. We searched for the lead variants representing the 185 loci in the summary statistics of the 42 quantitative traits tested. There were 242 associations at a nominal significance p-value cutoff 0.05. We then assigned a "Quantitative.GH.Score" to each loci:

- 0 (None): No significant associations to related quantitative traits
- 1 (Moderate): Significant associations to related quantitative traits
- 2 (Strong): Significant associations to quantitative traits directly involved in the diagnosis or presentation of the binary trait

(Again, we acknowledge that there is some subjectivity when deciding whether a quantitative trait is related to a binary trait.)

Of the 185 recessive loci reported in our study, 19 (~10%) had a score of 2, meaning that the lead variant had significant associations with quantitative traits directly related to the binary trait. Notably, 10 of these loci relate to the chromosome 11 associations with thalassaemia and hereditary anaemias, and

they were all significantly associated with red blood cell derangements related to anaemia (Note S11), which was expected.

An additional 14 loci (~8%) had a score of 1, demonstrating significant associations to quantitative traits that were related to the binary trait. They provide insight into possible underlying mechanisms that lead to the association between the variant and the binary trait. For example, we reported a recessive association between the variant chr1:31215264:T:C and increased likelihood of seborrhoeic dermatitis (recessive OR 1.3 and p-value 2.8×10^{-9}), and the additive GWAS reported an association between the variant and decreased vitamin D levels (beta -0.027 and p-value 3.5×10^{-2}). There is further evidence in the literature of decreased vitamin D levels being associated with seborrhoeic dermatitis [18–20], suggesting that vitamin D may play a role in the pathophysiology of seborrhoeic dermatitis. Indeed, this relationship has been hypothesised to be due to vitamin D's role in the inflammatory cascade [18,20,21]. As mentioned in the main text discussion, the liability threshold model is often applied to model binary traits. This finding provides additional support for this model - it is possible that this variant has an additive effect on vitamin D levels, and after a certain threshold, the vitamin D deficiency becomes disease-causing, and therefore the variant has a recessive association with seborrhoeic dermatitis.

About 82% of the recessive loci did not demonstrate significant associations to related quantitative traits. There are several reasons for this. Firstly, the quantitative blood traits were extracted from the electronic health records, and the context surrounding when these tests were taken is not accounted for, even

after the trait is normalised across the cohort. Therefore, for transient derangements in quantitative measurements that are related to the binary trait, we may not be able to pick them up in the quantitative trait GWAS. Secondly, many of these binary trait associations do not have known quantitative blood-based biomarkers for us to definitively call a significant quantitative trait association as an association to a “related trait”. For example, the variant chr5:5261658:G:A has a recessive association to the ICD10 code E14 (Unspecified diabetes mellitus). It also has additive associations to eosinophil and neutrophil levels. It is possible that an underlying immunologic cause is responsible for the diabetes association, however there is too little support from this analysis alone, and we have therefore assigned this finding a score of 0. Therefore, potentially with more relaxed definitions, or further investigation into these associations, we may be able to uncover more biological insight. As an extension to this, binary traits such as psychiatric conditions (e.g. Anxiety) may not have detectable changes in known, routinely-taken, blood-based biomarkers. Lastly, it is also possible that these variants may act recessively on not just the binary trait, but on the related quantitative trait as well. Therefore, they may still be missed out in the additive GWAS of quantitative traits.

In order to quantify the extent to which these reasons contribute to our findings (or lack thereof), we assign an additional score to the binary phenotypes, called “Phenotype with Relevant Quantitative Trait Score”, grading them based on the likelihood of them having a derangement in a quantitative blood-based measurement:

- 0: Unlikely to be associated with a quantitative blood-based measurement that has been tested. Examples of the binary phenotypes in this category include psychiatric disorders such as Anxiety, and mild dermatological conditions such as Melanocytic Naevi.
- 1: Possible association to a related blood-based measurement that has been tested, such as non-specific inflammatory markers. Or possible association to acute and transient derangements that may or may not be captured in the blood tests taken for the quantitative association analyses.
- 2: Association to a related blood-based measurement is expected. This includes binary phenotypes that require a blood-based measurement as part of the diagnosis (e.g. anaemia, diabetes), as well as chronic conditions that are likely to have blood-based derangements eventually captured in some of the testing used for quantitative association analyses.

Of the 185 recessive associations, 77 (42%) are associated with binary phenotypes with a “Phenotype with Relevant Quantitative Trait Score” of 0 and are not expected to have quantitative trait associations. Next, a further 54 are associated with binary phenotypes with a “Phenotype with Relevant Quantitative Trait Score” of 1, of which 12 (22% of the 54) have significant associations to related quantitative traits (“Quantitative.GH.Score” of 1), and 2 (4% of the 54) have a “Quantitative.GH.Score” of 2. Finally, in the remaining 54 associations that are associated with binary phenotypes that are expected to have an association with a related blood-based measurement (“Phenotype with

Relevant Quantitative Trait Score” of 2), 17 (31% of the 54) indeed have significant associations to quantitative traits directly involved in the diagnosis or presentation of the binary trait (“Quantitative.GH.Score” of 2) with an additional 2 (4% of the 54) having a “Quantitative.GH.Score” of 1.

In summary, about 35%, or 19 of 54 binary phenotypes expected to be associated with quantitative traits that have been tested do indeed reflect the relevant quantitative associations. As this categorisation is slightly subjective, we think that some of the reasons stated above for why the other associations did not demonstrate significant associations to related quantitative traits still apply. Nevertheless, from this analysis, we have managed to gain insight into the possible underlying biology contributing to these recessive binary trait associations (at least 18-35% of our findings) from evaluating the additive GWAS on quantitative traits.

A systematic review of the variants associated with thalassaemia and hereditary anaemias

On 22 October 2024, for the chromosome 11 variants associated with thalassaemia and hereditary anaemias, we also searched for expression quantitative trait loci (eQTL) results in genetics.opentargets.org. Positive results were collated in Note S11.

Note S11: A compilation of literature support for the 185 recessive loci

The document is available at

https://drive.google.com/file/d/1fxM1hmQPEozUAUIChQ-q1Md8ohyU2ZBA/view?usp=sharing

Note S12: FinnGen information, ethics statement, materials and methods

FinnGen was launched in 2017 (https://www.finngen.fi/), and it is a pre-competitive collaboration between biobanks in Finland and their supporting organisations such as universities and university hospitals. There is also involvement from international partners from the pharmaceutical industry, and the Finnish biobank cooperative (FINBB). All the FinnGen partners are listed here: https://www.finngen.fi/en/partners.

Patients and control subjects in FinnGen provided informed consent for biobank research, based on the Finnish Biobank Act. Alternatively, separate research cohorts, collected prior the Finnish Biobank Act came into effect (in September 2013) and start of FinnGen (August 2017), were collected based on study-specific consents and later transferred to the Finnish biobanks after approval by Fimea (Finnish Medicines Agency), the National Supervisory Authority for Welfare and Health. Recruitment protocols followed the biobank protocols approved by Fimea. The Coordinating Ethics Committee of the Hospital District of Helsinki and Uusimaa (HUS) statement number for the FinnGen study is Nr HUS/990/2017.

The FinnGen study is approved by Finnish Institute for Health and Welfare (permit numbers: THL/2031/6.02.00/2017, THL/1101/5.05.00/2017, THL/341/6.02.00/2018, THL/2222/6.02.00/2018, THL/283/6.02.00/2019, THL/1721/5.05.00/2019 and THL/1524/5.05.00/2020), Digital and population data service agency (permit numbers: VRK43431/2017-3, VRK/6909/2018-3,

VRK/4415/2019-3), the Social Insurance Institution (permit numbers: KELA 58/522/2017, KELA 131/522/2018, KELA 70/522/2019, KELA 98/522/2019, KELA 134/522/2019, KELA 138/522/2019, KELA 2/522/2020, KELA 16/522/2020), Findata permit numbers THL/2364/14.02/2020, THL/4055/14.06.00/2020, THL/3433/14.06.00/2020, THL/4432/14.06/2020, THL/5189/14.06/2020, THL/5894/14.06.00/2020, THL/6619/14.06.00/2020, THL/209/14.06.00/2021, THL/688/14.06.00/2021, THL/1284/14.06.00/2021, THL/1965/14.06.00/2021, THL/5546/14.02.00/2020, THL/2658/14.06.00/2021, THL/4235/14.06.00/2021, Statistics Finland (permit numbers: TK-53-1041-17 and TK/143/07.03.00/2020 (earlier TK-53-90-20) TK/1735/07.03.00/2021, TK/3112/07.03.00/2021) and Finnish Registry for Kidney Diseases permission/extract from the meeting minutes on 4th July 2019.

The Biobank Access Decisions for FinnGen samples and data utilized in FinnGen Data Freeze 10 include: THL Biobank BB2017_55, BB2017_111, BB2018_19, BB_2018_34, BB_2018_67, BB2018_71, BB2019_7, BB2019_8, BB2019_26, BB2020_1, BB2021_65, Finnish Red Cross Blood Service Biobank 7.12.2017, Helsinki Biobank HUS/359/2017, HUS/248/2020, HUS/150/2022 § 12, §13, §14, §15, §16, §17, §18, and §23, Auria Biobank AB17-5154 and amendment #1 (August 17 2020) and amendments BB_2021-0140, BB_2021-0156 (August 26 2021, Feb 2 2022), BB_2021-0169, BB_2021-0179, BB_2021-0161, AB20-5926 and amendment #1 (April 23 2020)and it´s modification (Sep 22 2021), Biobank Borealis of Northern Finland_2017_1013, 2021_5010, 2021_5018, 2021_5015, 2021_5023, 2021_5017, 2022_6001, Biobank of Eastern Finland 1186/2018 and

amendment 22 § /2020, 53§/2021, 13§/2022, 14§/2022, 15§/2022, Finnish Clinical Biobank Tampere MH0004 and amendments (21.02.2020 & 06.10.2020), §8/2021, §9/2022, §10/2022, §12/2022, §20/2022, §21/2022, §22/2022, §23/2022, Central Finland Biobank 1-2017, and Terveystalo Biobank STB 2018001 and amendment 25th Aug 2020, Finnish Hematological Registry and Clinical Biobank decision 18th June 2021, Arctic biobank P0844: ARC_2021_1001.

Supplemental figures

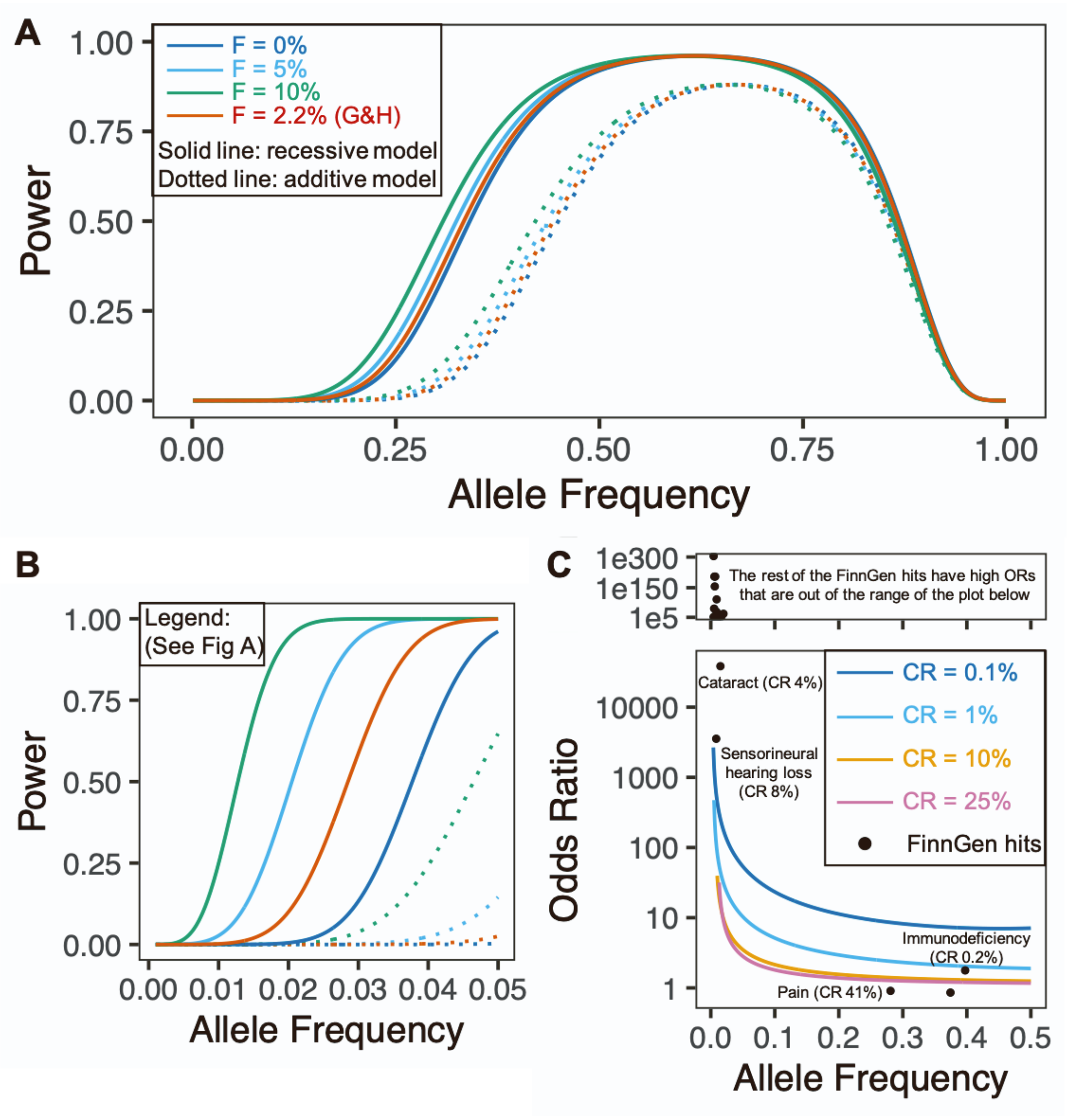

Figure S1

Simulations to evaluate power to detect a recessive effect in G&H when using a recessive versus additive model. A: Calculating power to detect an association at $p<5x10^{-8}$ for a range of allele frequencies and values for average inbreeding coefficient (F). Sample size = 44,000, OR = 2, CR = 1%. B: Calculating power for a range of rarer allele frequencies and values for F. Parameters as per A, except OR = 5. C: Calculating the minimum odds ratio

G&H would be powered to detect at 80% power, and F = 2.2%. ”CR” : Case rate. Recessive hits reported in FinnGen [3] are indicated on the plot, showing their OR and AF estimated in FinnGen.

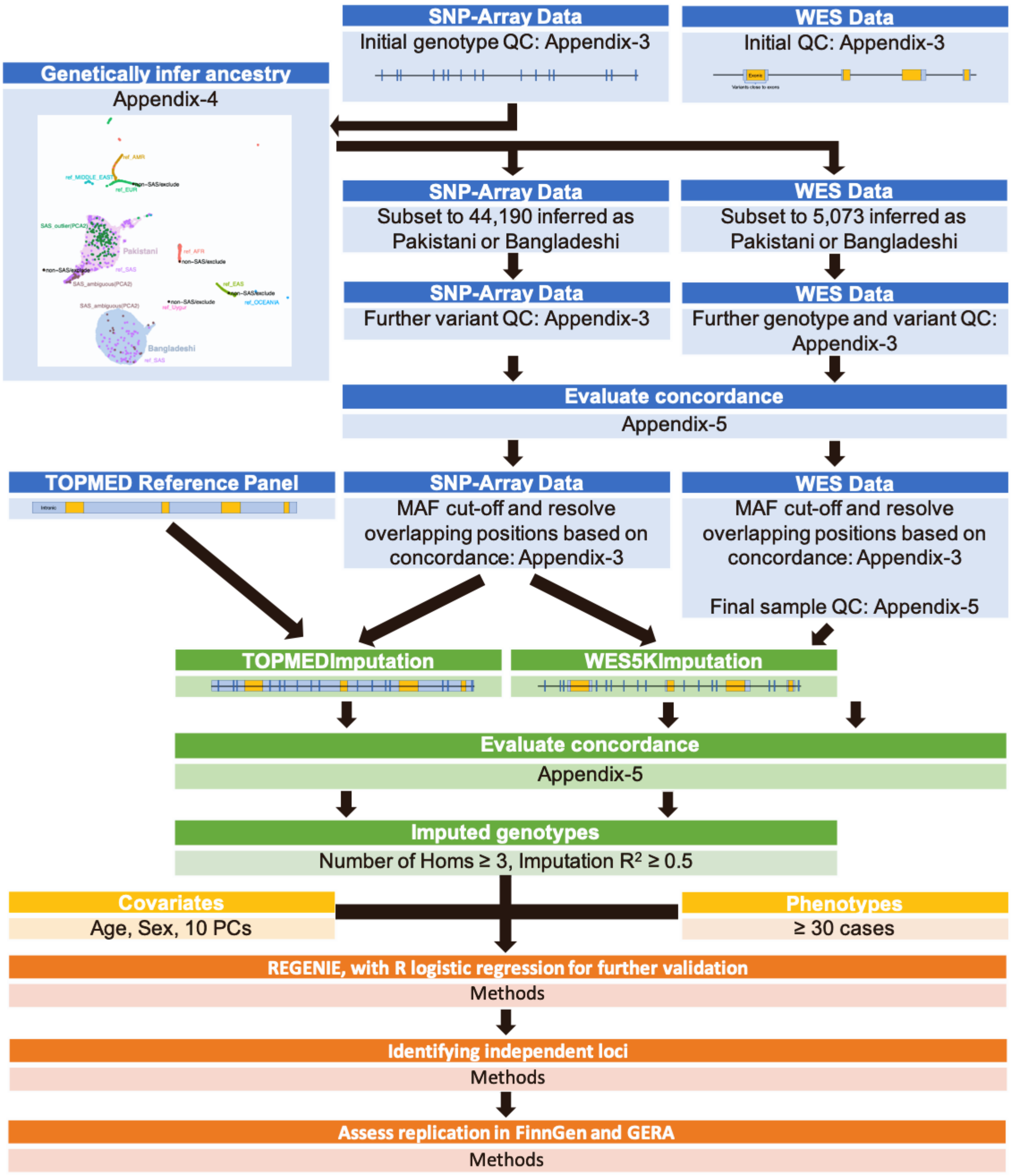

Figure S2

A flowchart to visualise the methods of this project. This is intended to serve as a directory to relevant sections.

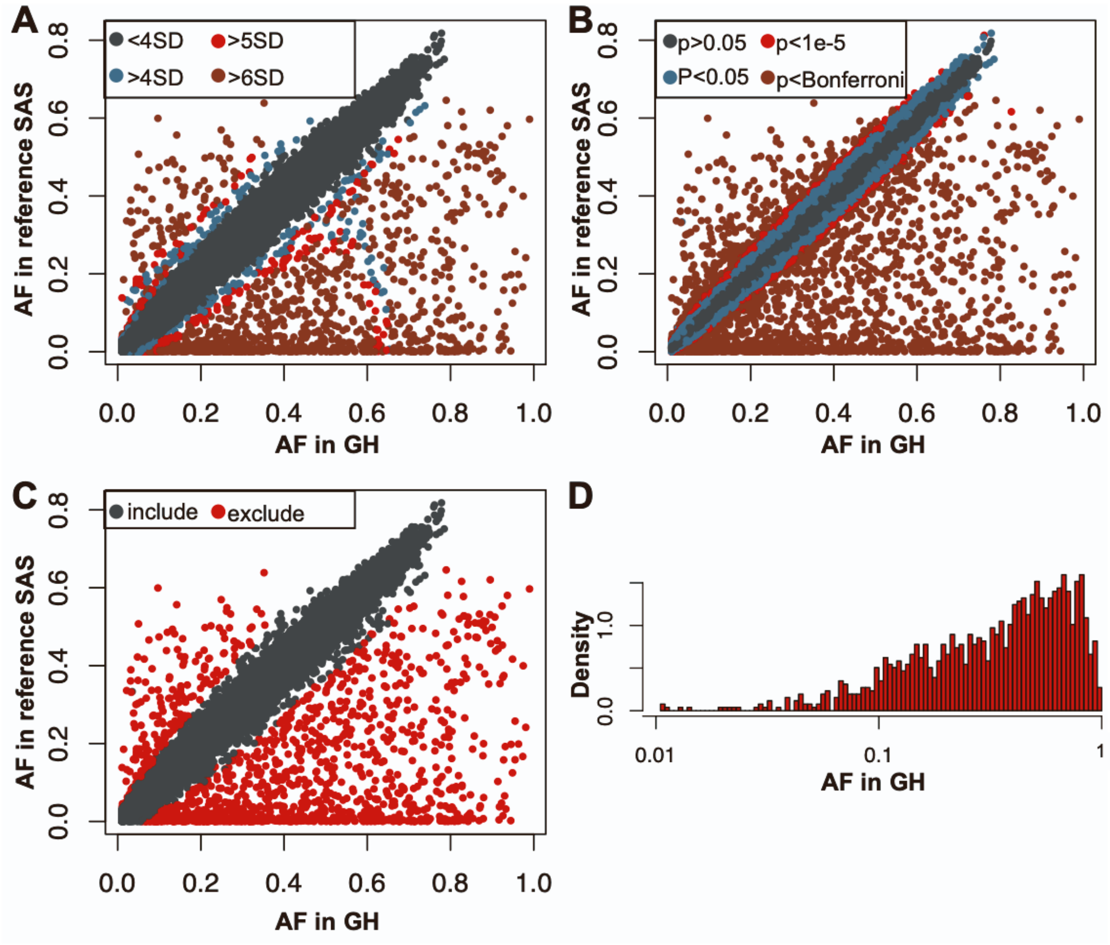

Figure S3

Excluding variants with significantly different AF in Genes and Health (GH) and 676 South Asian samples from 1000 Genomes and HGDP (reference SAS). Plotting the AFs in both cohorts for each variant, colour-coding represents in A: the different standard deviation (SD) thresholds applied to the mean of the residuals in 0.01 frequency bins, in B: the different p-value thresholds for Fisher's exact tests on the genotypes, and in C: the variants that were included and excluded from the merged panel of variants. D: The AF distribution of the variants excluded.

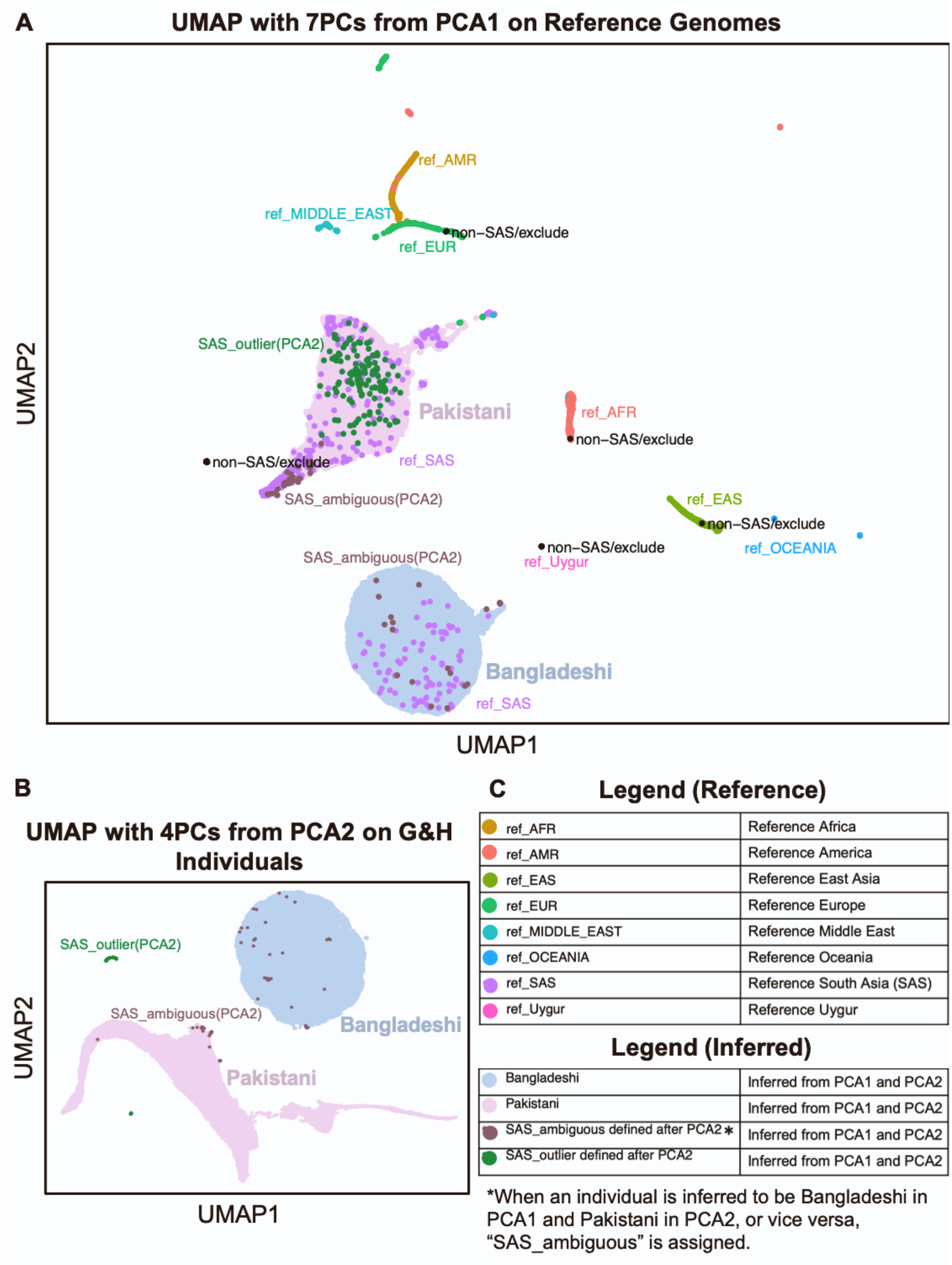

Figure S4

Genetic inference of ancestry using the G&H SNP-array data. A: UMAP with 7 PCs from the PCA of reference individuals (PCA1), with G&H individuals

projected into the PC space. B: UMAP with 4 PCs from the PCA of unrelated G&H individuals (PCA2), with related G&H individuals projected into the PC space. C: Legends for both UMAPs.

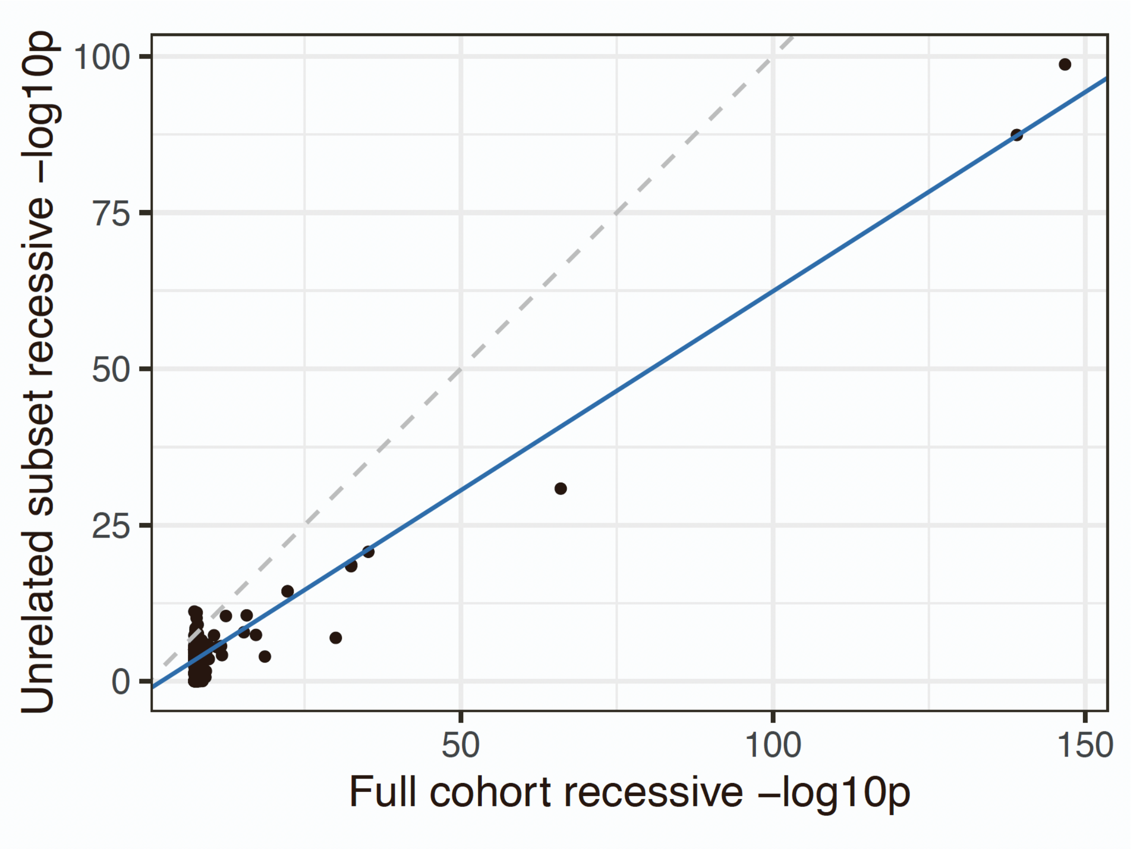

Figure S5

P-values (in -log10p) of the 185 lead variants from the recessive analysis performed on the unrelated subset (y-axis) compared to the recessive analysis performed on the full cohort (x-axis). The blue solid line represents the line of best fit, while the grey dashed line represents the x=y line.

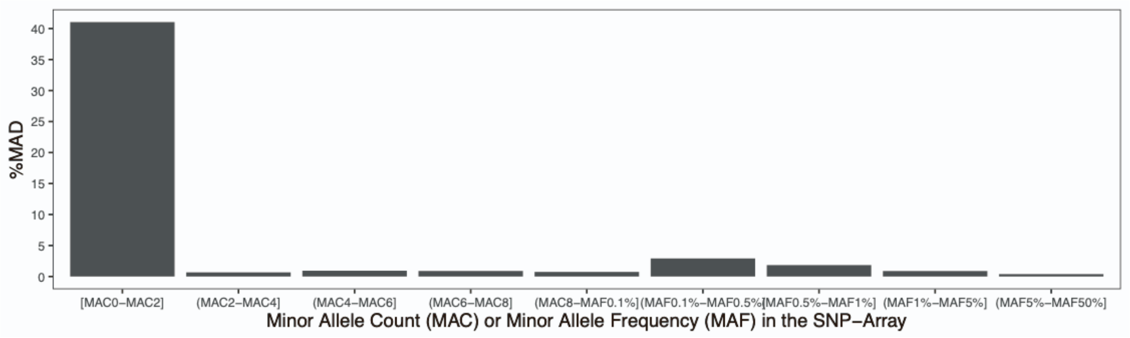

Figure S6

The Minor Allele Discordance (MAD) between the SNP-Array and the WES at overlapping variants, stratified by the frequency on the array. Array filters: After initial genotype QC + Call rate ≥99% + HWE pval $\geq 10^{-6}$. WES filters: Call rate ≥70% after GQ ≥20 + binomAD $\geq 10^{-2}$ + DP>7. Here, the array genotypes were treated as "truth". As N~5,000, a MAC of 2 converts to $\sim 2\times 10^{-4}$ (or ~0.02%) in MAF.

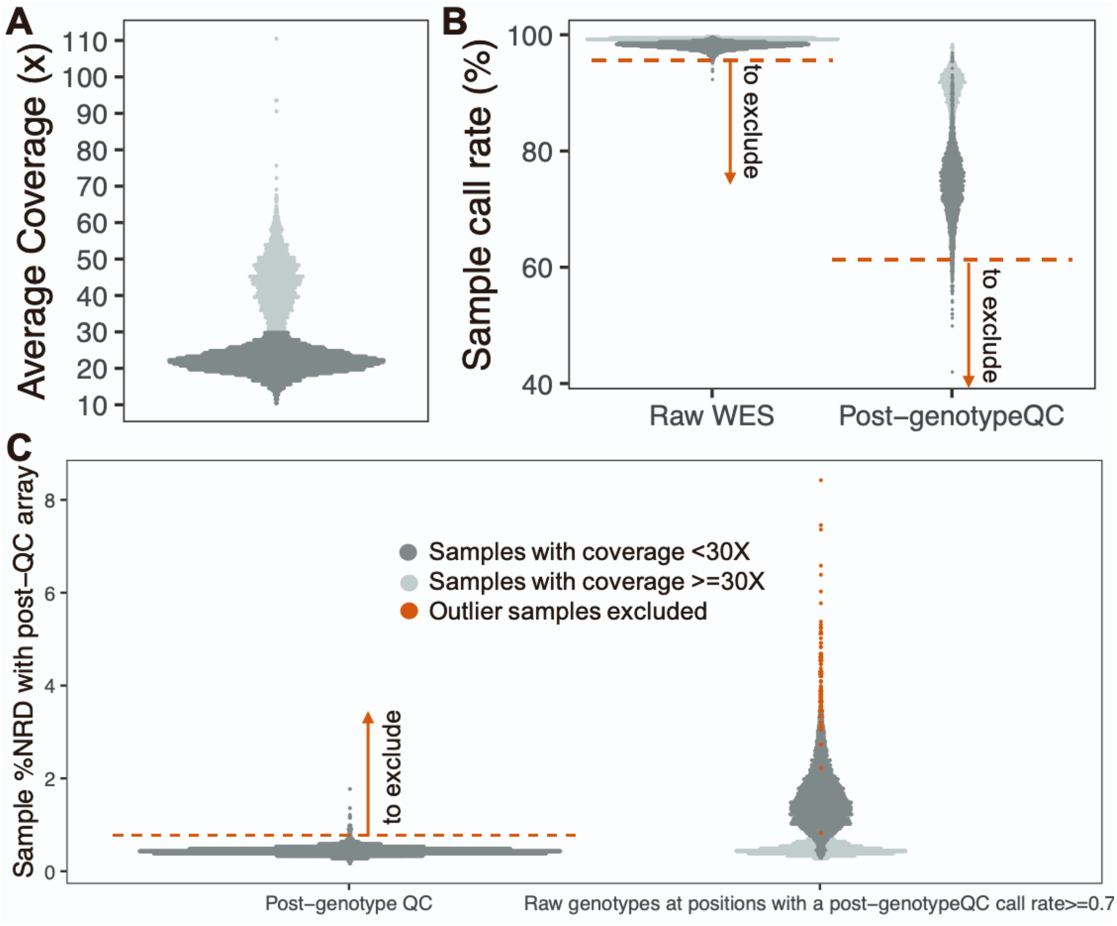

Figure S7

Per-sample coverage, call rate, and non-reference discordance (NRD) before and after QC of WES data. A: Distribution of average on-target coverage across samples. The bimodality observed is because different batches of samples were sequenced to either ~20X or ~40X. B: The distribution of WES sample call rates pre-genotype QC (left) and post-genotype QC (right). C: The distribution of NRDs across samples in the post-genotype QC WES. In B and C, the bimodality is again likely due to the different sequencing coverage.

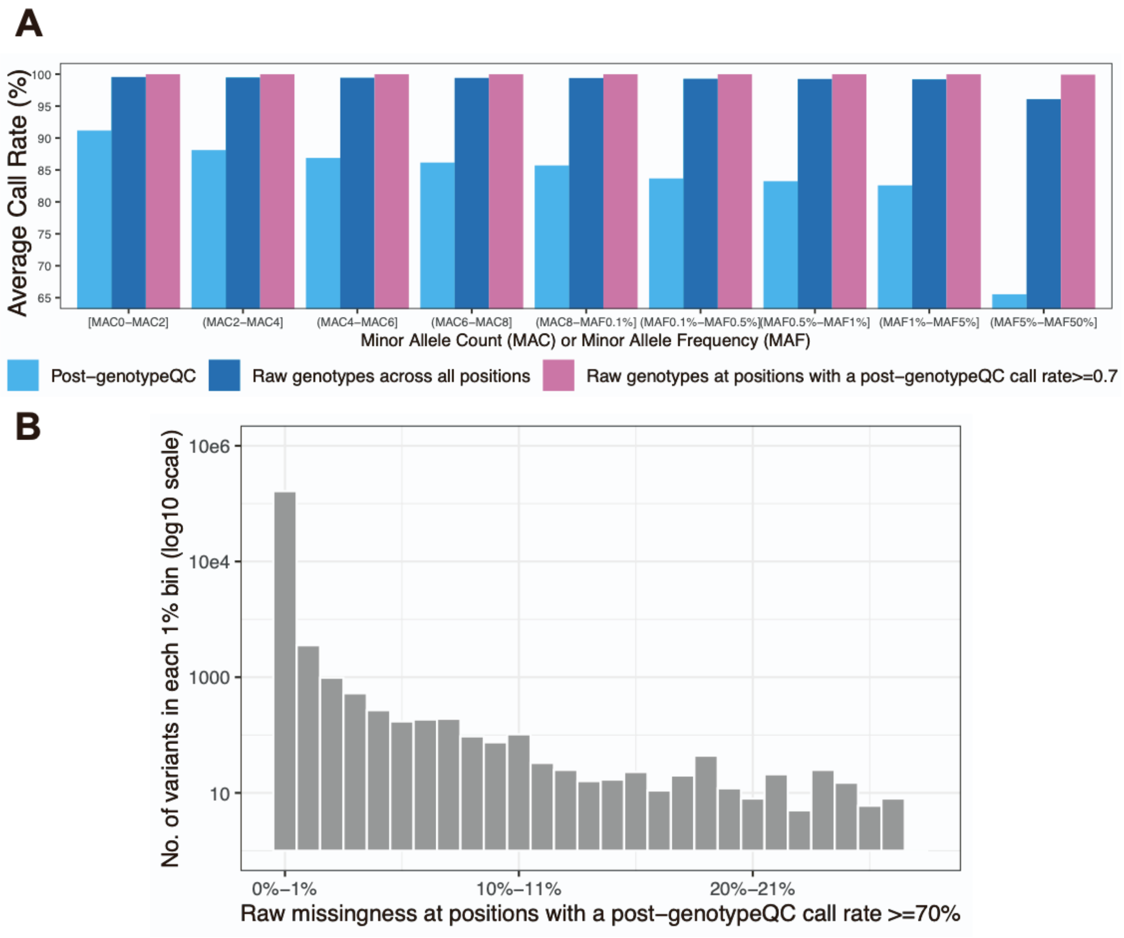

Figure S8

Distribution of call rates pre and post QC in the G&H WES data. A: Average call rate stratified across MAF bins. As the missingness is low in the raw WES, when filtering to positions with a post-genotypeQC call rate ≥70%, the call rate of the raw genotypes is >99% across the frequency spectrum. As N~5,000, a MAC of 2 converts to ~2x10^{-4} (or ~0.02%) in MAF. B: Distribution of per-variant raw missingness at positions with a post-genotype QC call rate ≥70%. The y-axis has been transformed to a log 10 scale as an overwhelmingly large number of variants have a raw missingness of 0-1%.

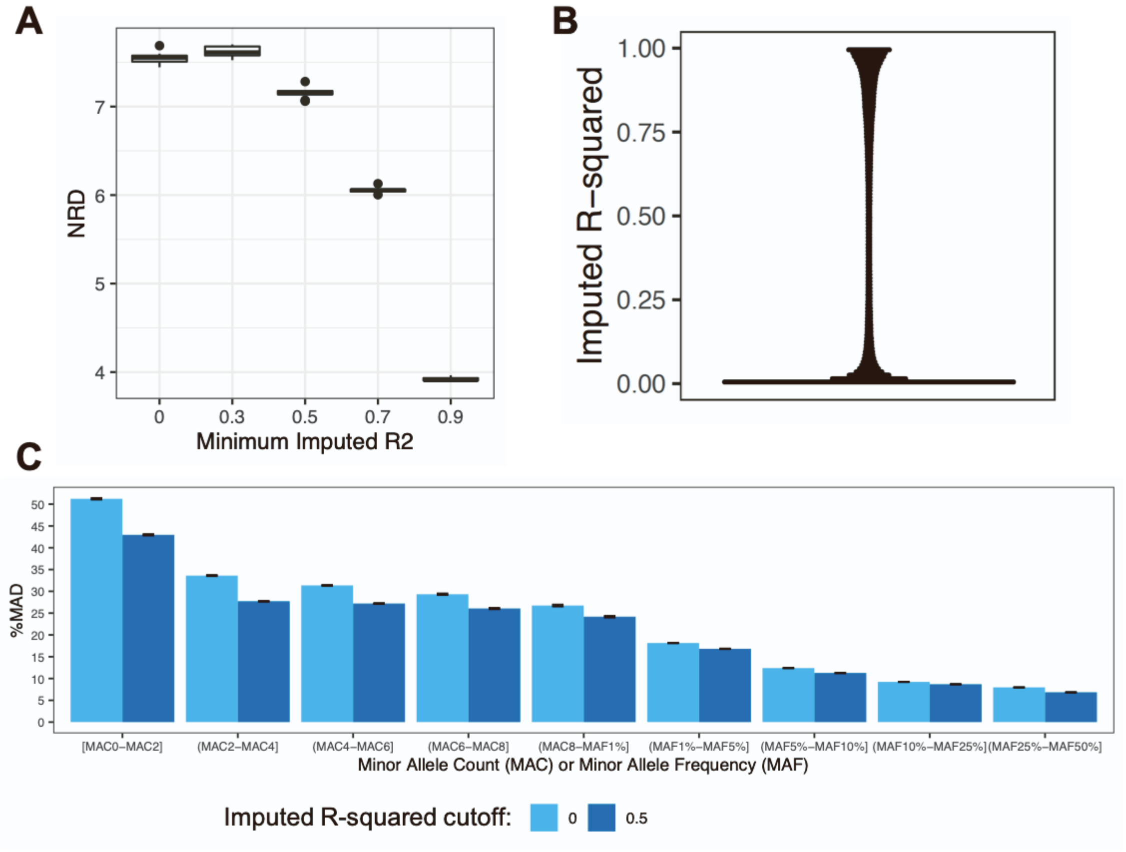

Figure S9

Results of the leave-10%-out trials. A: The distribution of NRD(%) across 10 trials when different minimum imputed r^2 cutoffs are applied. B: The distribution of imputed r^2 scores for a representative trial. C: The MAD before and after applying an imputed r^2 ≥0.5 cut-off, stratified by MAF. (Error bars are the standard errors in %MAD across the 10 trials.) As the sample size is 10% each time, i.e. N~500, the MAC of 2 converts to ~$2x10^{-3}$ (or ~0.2%) in MAF.

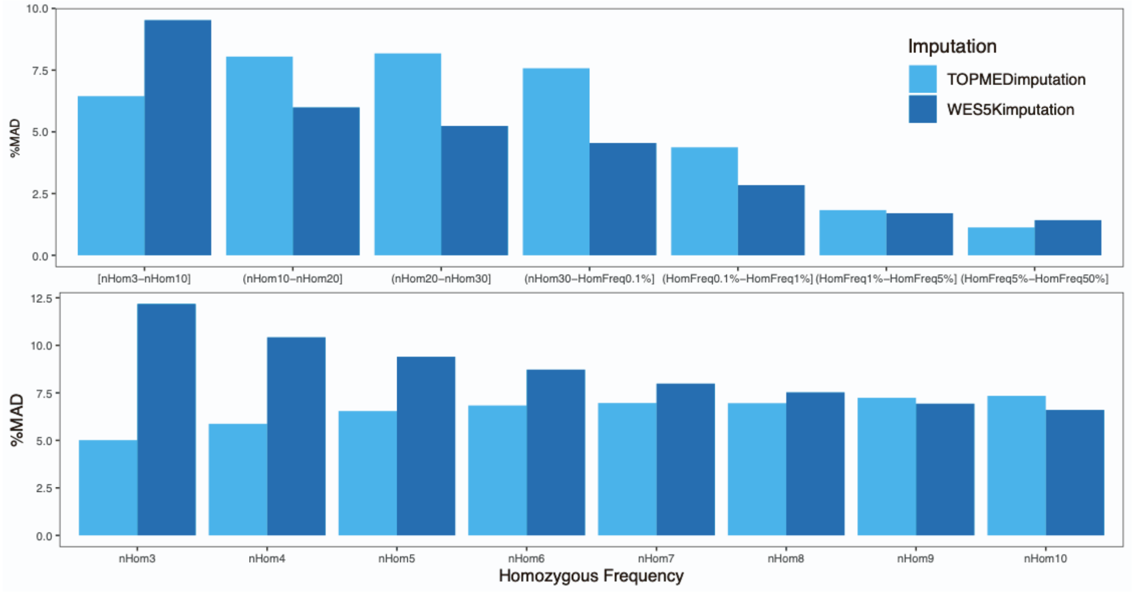

Figure S10

Minor allele discordance (MAD) of the TOPMEDimputation compared to the WES, and the mean MAD across ten leave-10%-out trials compared to the WES (represented as the WES5Kimputation for simplicity), stratified by the number or frequency of homozygous genotypes in the WES data. The variant counts contributing to each bin is tabulated below.

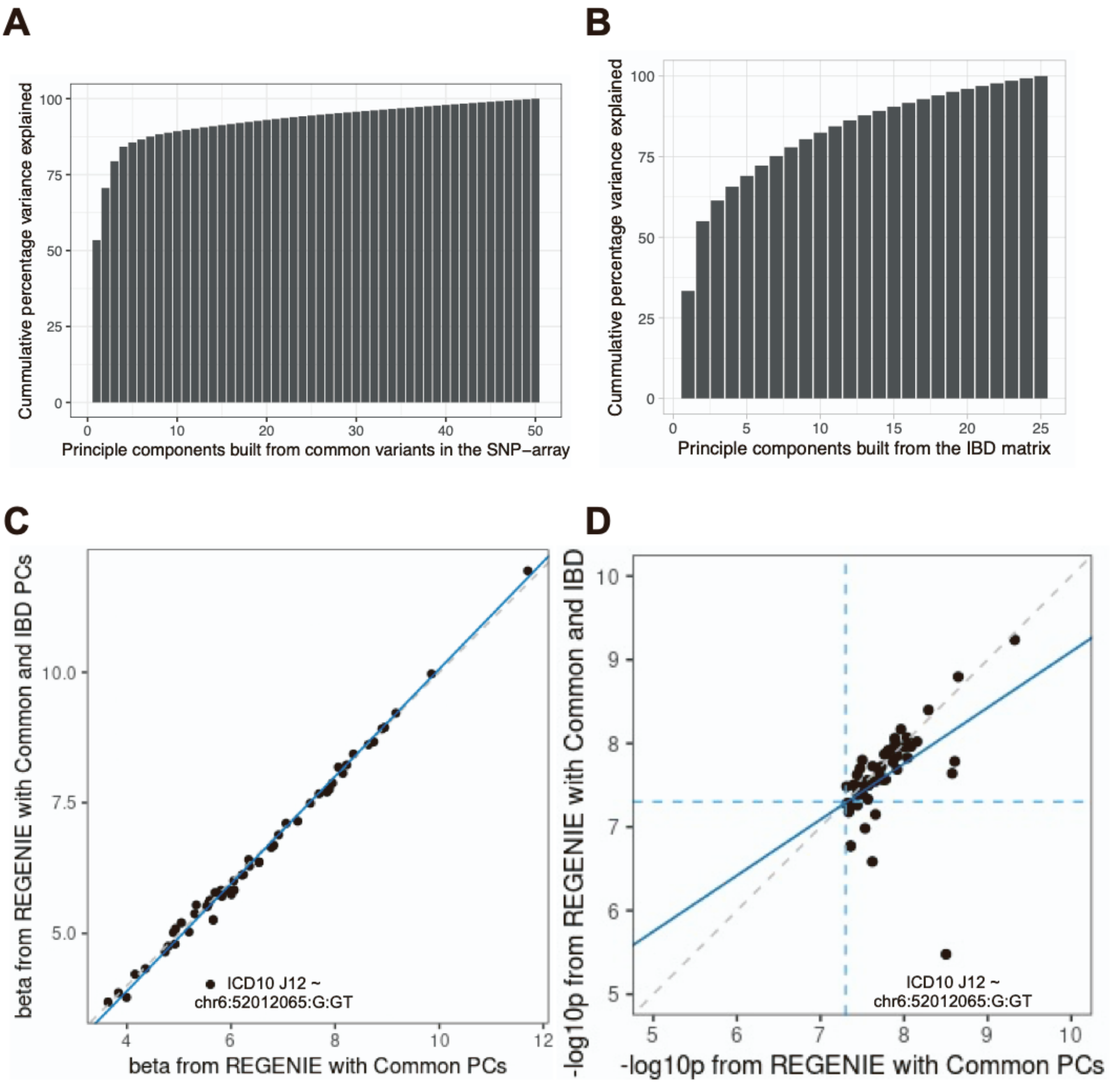

Figure S11

Principal component analyses of population structure. A: The cumulative percentage variance explained across the 50PCs generated from common variants in the SNP-array, with the variance explained by all 50PCs scaled to 100%. B: The cumulative percentage variance explained across the 25PCs generated from the IBD matrix, with the variance explained by all 25PCs scaled to 100%. C&D: For the 56 recessive loci associated with rare (AF 1% and lower) variants, the association analysis was repeated controlling for 10 common variant PCs and 15 IBD PCs, and the betas (C) p-values (D) were

compared. The blue solid line represents the line of best fit, the grey dashed line represents the x=y line, the blue dashed line represents the $p=5x10^{-8}$ cutoff. The outlier finding has been labelled.

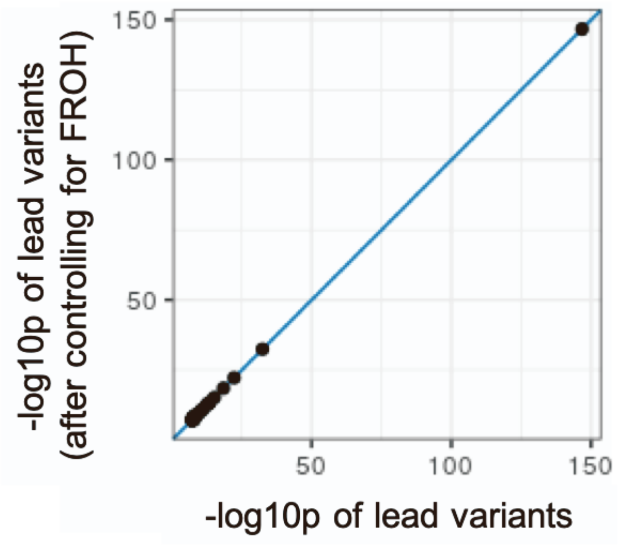

Figure S12

Scatter plot of the WES5Kimputation lead variants p-values (-log10p) compared to their p-values when the recessive test is rerun to control for F_{ROH}.

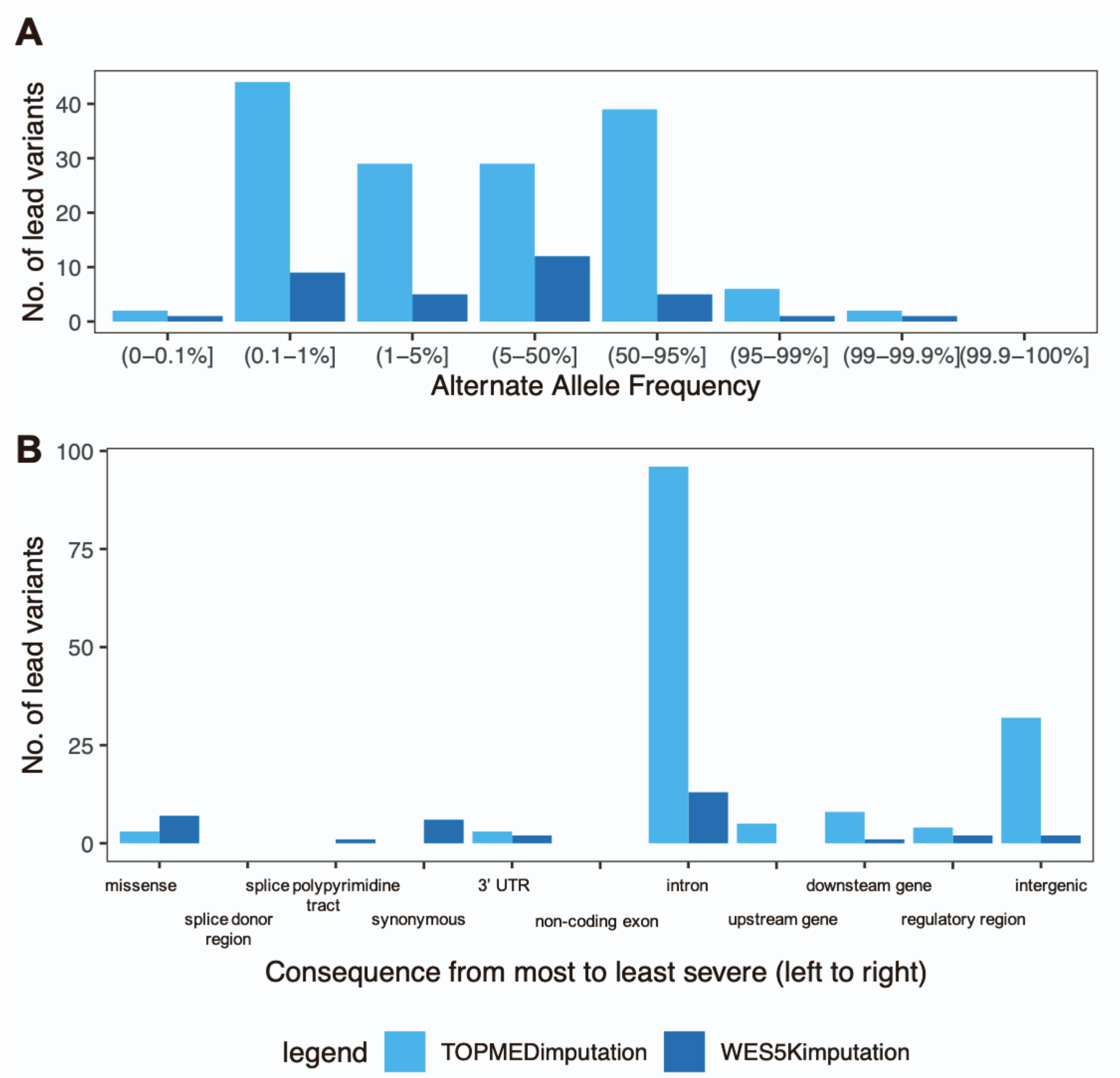

Figure S13

Characteristics of lead SNPs in the recessive findings. A: Distribution of the allele frequencies of the lead SNPs. B: Distribution of the variant consequences of the lead SNPs.

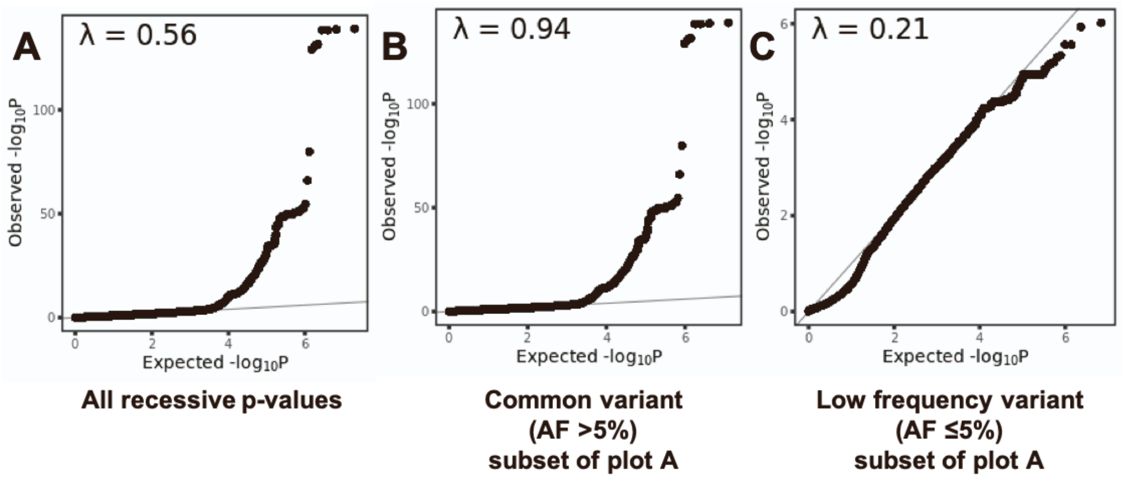

Figure S14

Quantile-quantile (QQ) plots (B-D) for the phenotypes that have Bonferroni-significant findings. In A, all the recessive p-values in the D58[Other hereditary haemolytic anaemias] run have been plotted. We then split the QQ-plot into common variants (AF > 5%, B) and low frequency variants (C).

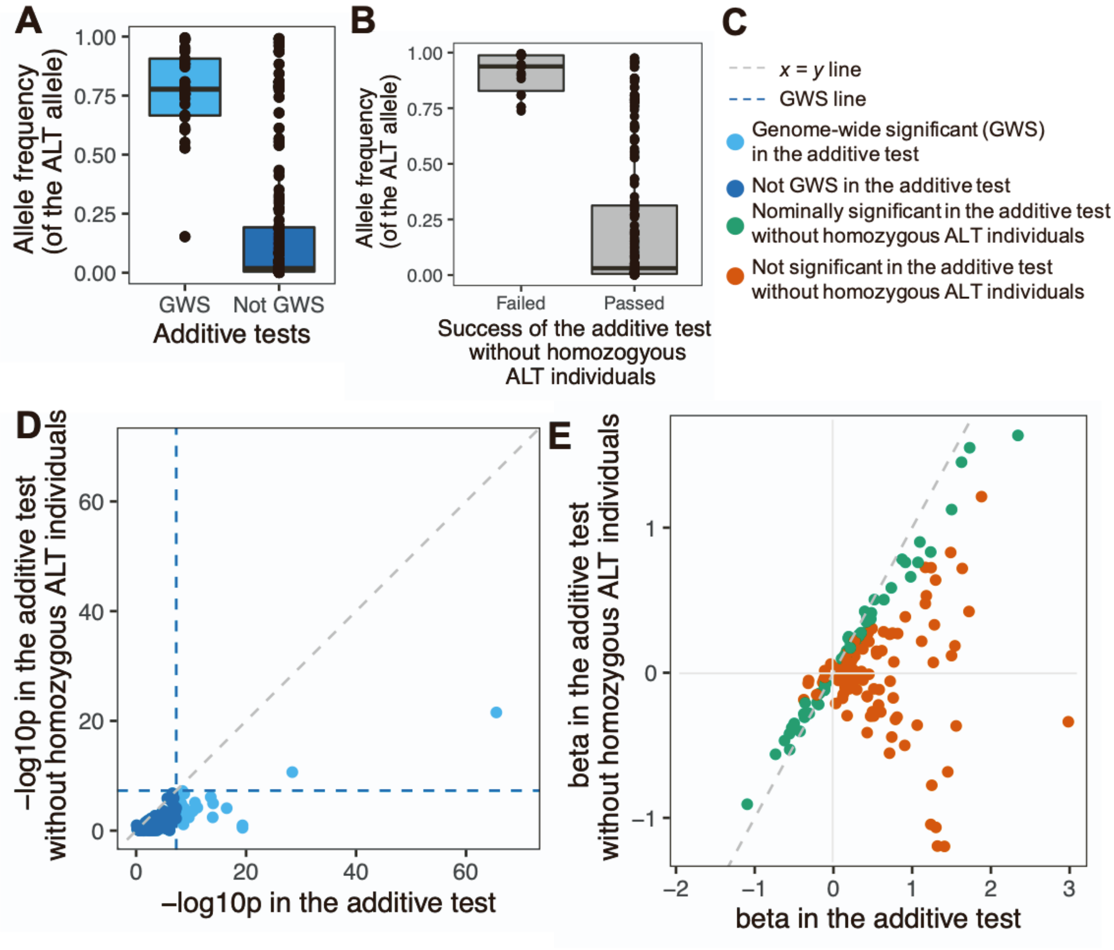

Figure S15

Exploring the recessive lead variants with different models of testing in REGENIE. A: AF distribution of recessive hits that were GWS and not GWS in additive tests. B: AF distribution of recessive hits that could and could not run under the additive model without homozygous individuals. C: legend for the figure. D, E: P-values (-log10p, D) and betas (E) in the additive tests compared to the additive tests without homozygotes.

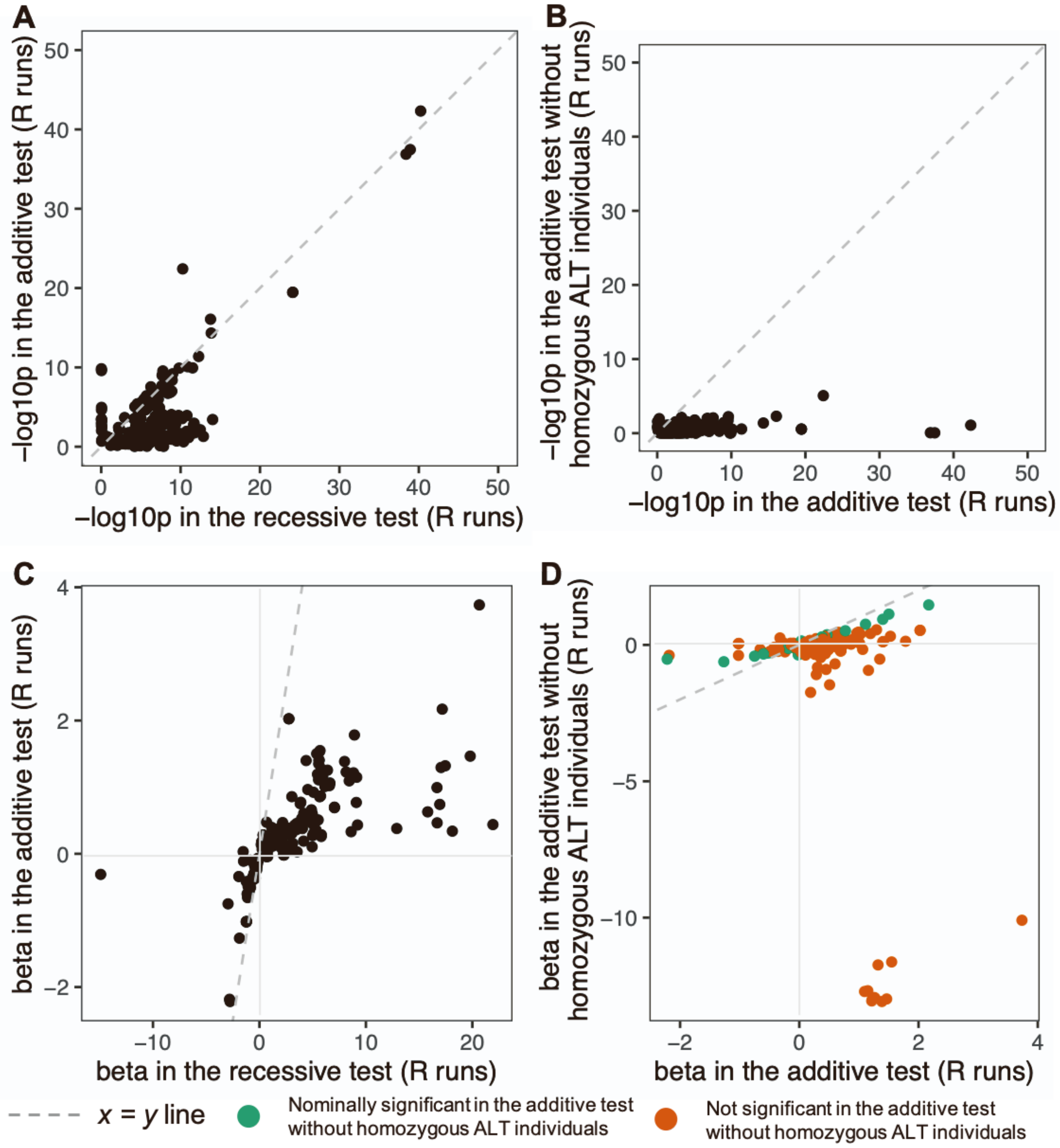

Figure S16

Exploring the recessive lead variants with different models of testing in R.

A,C: P-values (-log10p, A) and betas (C) in the recessive tests compared to the additive tests in R. B,D: P-values (-log10p, B) and betas (D) in the additive tests compared to the additive tests without homozygotes in R.

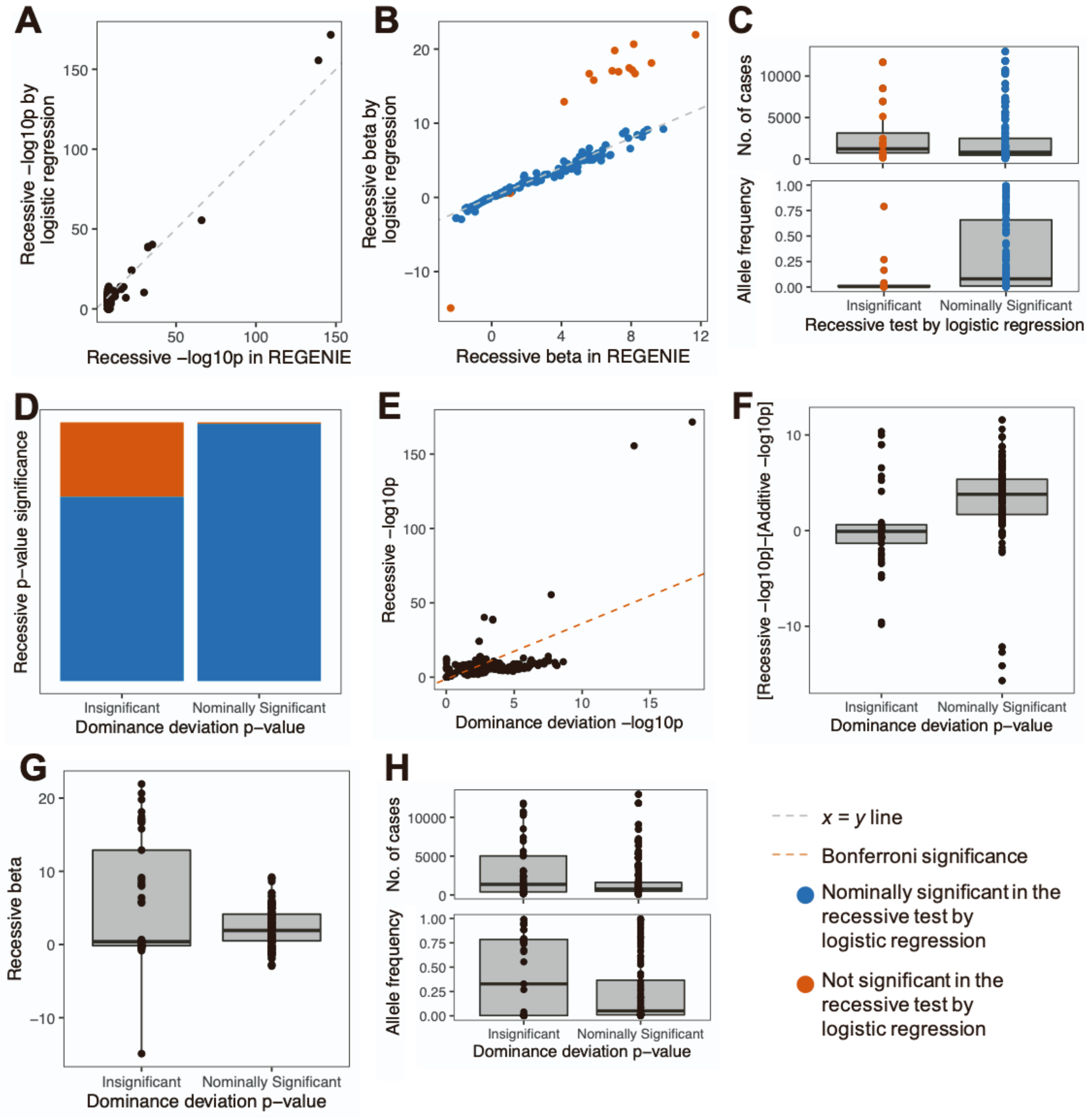

Figure S17

Logistic regression testing in R for the recessive findings. A,B: P-values (-log10p, A) and betas (B) in REGENIE compared to logistic regression testing. C: Distributions of AF and case counts between nominally significant and insignificant (p-value > 0.05) tests in the recessive logistic regression. D-F: Distribution of recessive logistic regression p-values(D-E), the differences between the recessive -log10p and the additive -log10p (F), betas (G), and AF and case counts (H) across the dominance deviation tests.

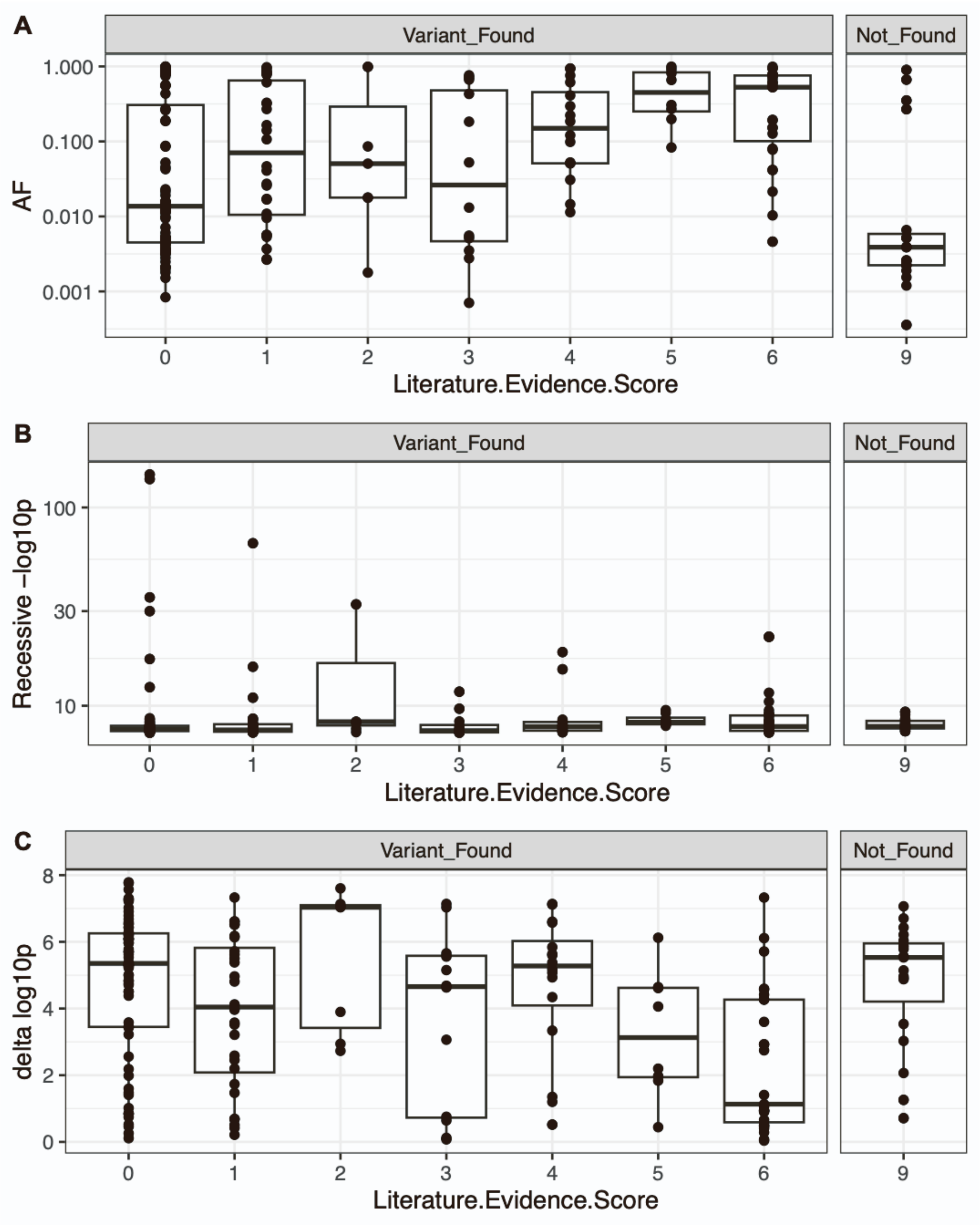

Figure S18

Lead variant characteristics plotted across Literature.Evidence.Scores. A: Allele frequencies (AFs) have been plotted. B: Recessive -log10p values have been plotted. C: Delta log10p (Subtracting the additive -log10p from the recessive -log10p) have been plotted.

Supplemental tables

<table>
<tr><th></th><th>SNP-Array Data</th><th>WES Data</th></tr>
<tr><td>Initial QC</td><td>Initial genotype QC
44,396 individuals
Variant QC in GenomeStudio
Sample QC (exclude samples with low call rate or failed sex checks)</td><td>5,236 individuals after light sample QC (exclude samples with low coverage or sex discrepancies)</td></tr>
<tr><td>Genetically infer ancestry</td><td>44,190 individuals assigned as Pakistani or Bangladeshi</td><td>5,073 individuals with array data and assigned as Pakistani or Bangladeshi</td></tr>
<tr><td>Genotype QC</td><td></td><td>Set genotype as missing when: GQ <20, binomAD <10^{-2}, DP ≤7</td></tr>
<tr><td rowspan="4">Variant QC</td><td>534,806 autosomal SNPs</td><td>2,245,547 autosomal SNPs and indels, after excluding:
For SNPs: "QD < 2.0 || FS > 30 || MQ < 40.0 || MQRankSum < -12.5 || ReadPosRankSum < -8.0"
For indels: "QD < 2.0 || FS > 30 || ReadPosRankSum < -20.0"</td></tr>
<tr><td>534,531 variants with a call rate ^{3}99%</td><td>1,645,161 variants with a post-genotype QC call rate ^{3}70%</td></tr>
<tr><td>533,362 variants with HWE p>=10^{-6} in the Bangladeshi subgroup</td><td></td></tr>
<tr><td>533,166 biallelic variants</td><td></td></tr>
<tr><td colspan="3">Evaluate Concordance</td></tr>
<tr><td>MAF cut-offs</td><td>Keep 469,678 variants with MAF >0.1%</td><td>Remove singletons</td></tr>
<tr><td rowspan="3">Resolve Overlapping Positions</td><td>Exclude 25 common palindromic variants with MAF >40%</td><td>Keep, as no problem of allele-switching</td></tr>
<tr><td>Exclude 515 variants with overlapping positions but unmatched alleles</td><td>Keep, as some are indels that have been genotyped incorrectly on the array</td></tr>
<tr><td>Keep 38,248 variants with matched alleles and MAF >0.1%</td><td>Exclude, as the array has a higher overall call rate
(Note: rare variants with MAF ≤0.1% that overlapped with the array are retained)</td></tr>
<tr><td>Final Sample QC</td><td></td><td>4,982 individuals after excluding: Pre-genotype QC call rate <4SD from mean, Post-genotype QC call rate <2SD from mean, Post-genotype QC NRD >4SD from mean</td></tr>
</table>

Table S1

QC steps for the SNP-Array and WES Data

	Raw Genotypes (GTs)	GQ >=20	Call rate >=70% after: GQ >=20 binomAD >=10^{-2} DP>7	With raw GT at sites with call rate >=70% after: GQ >=20 binomAD >=10^{-2} DP>7
**After initial genotype QC**	5.018% (46,778 SNPs)			
**Call rate >=99% HWE pval >=10-6**	5.000% (46,656 SNPs)	0.500 % (46,401 SNPs)	0.398% (38,249 SNPs)	1.258 % (38,249 SNPs)

Table S2

Overall non-reference discordance (NRD) between the array and WES data with different QC filters applied to the SNP-array data and the WES data. Row names describe stages of SNP-array filtering, column names describe stages of WES filtering. “Initial genotype QC” is described in the first row of Table S1.

	**n variants (TOPMEDimputation)**	**n variants (WES5Kimputation)**	**WES5Kimputation / TOPMEDimputation**
**nHom 3**	10,806	26,001	2.41
**nHom 4**	10,855	19,588	1.80
**nHom 5**	9,771	15,532	1.59
**nHom 6**	8,725	12,615	1.45
**nHom 7**	7,701	10,546	1.37
**nHom 8**	6,875	9,044	1.32
**nHom 9**	6,233	7,864	1.26
**nHom 10**	5,519	6,774	1.23
**(nHom 10 – nHom 20]**	35,924	41,259	1.15
**(nHom 20 – nHom 30]**	18,717	20,156	1.08
**(nHom 30 – HomFreq 0.1%]**	17,065	17,907	1.05
**(HomFreq 0.1% - HomFreq 1%]**	109,350	111,916	1.02
**(HomFreq 1% - HomFreq 5%]**	106,049	109,146	1.03
**(HomFreq 5% - HomFreq 10%]**	151,338	173,978	1.15

Table S3

Number of imputed variants (that can be compared to WES positions for concordance analyses) binned by the number or frequency of homozygous genotypes in the WES data.

Phenotypes	n phenotypes	n individuals	n variants (WES5KImputation)	n variants (TOPMEDImputation)
Custom phenotypes (In both sexes)	199	44,186	605,263	10,045,406
Custom phenotypes (Female only)	15	24,387	553,716	9,215,675
Custom phenotypes (Male only)	5	19,799	529,480	8,855,245
ICD10 codes (In both sexes)	565	42,027	599,758	9,948,708
ICD10 codes (Female only)	99	23,710	550,988	9,171,960
ICD10 codes (Male only)	15	18,317	522,117	8,741,181

Table S4

The number of phenotypes, individuals, and variants being tested. The number of variants tested varied with the number of individuals tested, as different subsets of individuals would result in different numbers of variants having at least three homozygotes.

	coding	non-coding
All tested	91,930	9,953,476
Recessive variants	40	1,090

Table S6

Table of the number of coding and non-coding variants tested in the TOPMEDImputation, compared to the number of coding and non-coding variants among the TOPMEDImputation variants with a genome-wide-significant recessive p-value that was smaller than their additive p-value, before defining loci and filtering by LD.

Genotype	Additive	Recessive	Dominance deviation
RR	0	0	0
RA	1	0	1
AA	2	1	0

Table S7

Genotype encodings for the additive, recessive and dominance deviation tests performed as logistic regression tests in R. “RR” refers to the homozygous wild type, “RA” the heterozygous genotype, and “AA” the homozygous alternate genotype.

Gene	Lead variant	AF	CSQ	Phenotype	OR	p-value	DD p-value
F_{ROH}-Associated Phenotypes:							
**A09[Other gastroenteritis and colitis of infectious and unspecified origin]**							
.	2:171680912:G:A	0.3	intergenic	A09[Other gastroenteritis and colitis of infectious and unspecified origin]	0.6	2.7E-08	6.0E-02
**E11[Type 2 diabetes mellitus]**							
.	6:104459545:G:A	0.01	regulatory region	E11[Type 2 diabetes mellitus]	38	3.6E-08	7.2E-06
Y_RNA	10:92706738:G:C	0.7	downstream gene	GNH0242 Type 2 Diabetes narrow	0.8	8.8E-09	1.0E-01
Y_RNA	10:92706682:A:G	0.8	downstream gene	Type 2 Diabetes	0.8	4.3E-09	5.6E-02
ADAMTS 16	5:5261658:G:A	0.01	intron	E14[Unspecified diabetes mellitus]	296	5.1E-09	6.9E-06
UBE2E2	3:23530638:C:A	0.001	intron	E14[Unspecified diabetes mellitus]	2664	4.6E-08	9.6E-01
SNX5	20:17949555:G:T	0.01	intron	GNH0244 Unspecified or Rare Diabetes narrow	63	1.4E-08	3.5E-03
**E78[Disorders of lipoprotein metabolism and other lipidaemias]**							
APOA5	11:116796367:A:G	0.9	upstream gene	E78[Disorders of lipoprotein metabolism and other lipidaemias]	0.8	3.3E-11	7.1E-03
**F41[Other anxiety disorders]**							
.	2:236834949:C:T	0.01	regulatory region	F40[Phobic anxiety disorders]	138	2.7E-08	2.6E-03
.	7:131782270:G:A	0.08	intergenic	Anxiety and phobia	1.8	3.5E-08	4.8E-03
.	7:131780486:G:A	0.08	intergenic	F41[Other anxiety disorders]	1.9	7.7E-10	2.7E-03
.	7:131773247:A:G	0.1	regulatory region	F41[Other anxiety disorders]	1.8	1.8E-08	7.7E-05
**H61[Other disorders of external ear]**							
ESRRG	1:216911690:AG:A	0.01	intron	H60[Otitis externa]	54	2.5E-09	4.7E-04
**J11[Influenza, virus not identified]**							
PKHD1	6:52012065:G:GT	0.004	intron	J12[Viral pneumonia, not elsewhere classified]	271	3.1E-09	6.6E-06
PKHD1	6:52012065:G:GT	0.004	intron	B97[Viral agents as the cause of diseases classified to other chapters]	180	2.7E-09	1.9E-05
.	6:51529735:A:G	0.002	intergenic	B97[Viral agents as the cause of diseases classified to other chapters]	430	2.4E-08	8.0E-04
**J34[Other disorders of nose and nasal sinuses]**							
ADAMTS 9-AS2	3:64802433:A:G	0.03	intron	J34[Other disorders of nose and nasal sinuses]	8	1.5E-08	1.8E-07
.	7:48916122:T:C	0.02	intergenic	J34[Other disorders of nose and nasal sinuses]	13	5.0E-09	1.5E-03

.	5:91760313:C:G	0.01	intron	J34[Other disorders of nose and nasal sinuses]	11	2.4E-08	1.5E-03
**L30[Other dermatitis]**							
PTPRN2	7:157970755:T:G	0.005	intron	L20[Atopic dermatitis]	64	1.1E-08	8.6E-01
NKAIN1	1:31215264:T:C	0.9	intron	L21[Seborrhoeic dermatitis]	1.3	6.2E-09	8.4E-01
BCAR3	1:93847011:TCGGGCGCGGCGG:*	0.4	intron	Dermatitis (atopic, contact, other, unspecified)	1.2	1.6E-08	6.3E-08

Table S10

Single recessive associations involving phenotypes found to be significantly associated with genome-wide homozygosity in Malawsky et al. (2023) [22].

Supplemental acknowledgements

We thank the Human Genetics Informatics team at the Wellcome Sanger Institute for support with variant annotations.

This research was funded in part by Wellcome (grant no. 220540/Z/20/A, “Wellcome Sanger Institute Quinquennial Review 2021–2026”). For the purpose of open access, the authors have applied a CC-BY public copyright licence to any author accepted manuscript version arising from this submission.

Genes & Health is/has recently been core-funded by Wellcome (WT102627, WT210561), the Medical Research Council (UK) (M009017, MR/X009777/1, MR/X009920/1), Higher Education Funding Council for England Catalyst, Barts Charity (845/1796), Health Data Research UK (for London substantive site), and research delivery support from the NHS National Institute for Health Research Clinical Research Network (North Thames). Genes & Health is/has recently been funded by Alnylam Pharmaceuticals, Genomics PLC; and a Life Sciences Industry Consortium of Astra Zeneca PLC, Bristol-Myers Squibb Company, GlaxoSmithKline Research and Development Limited, Maze Therapeutics Inc, Merck Sharp & Dohme LLC, Novo Nordisk A/S, Pfizer Inc, Takeda Development Centre Americas Inc.

T. H. Heng is supported by the Agency for Science, Technology, and Research (A∗STAR) National Science Scholarship.

We thank Social Action for Health, Centre of The Cell, members of our Community Advisory Group, and staff who have recruited and collected data

from volunteers. We thank the NIHR National Biosample Centre (UK Biocentre), the Social Genetic & Developmental Psychiatry Centre (King's College London), Wellcome Sanger Institute, and Broad Institute for sample processing, genotyping, sequencing and variant annotation.

This work uses data provided by patients and collected by the NHS as part of their care and support.

We thank: Barts Health NHS Trust, NHS Clinical Commissioning Groups (City and Hackney, Waltham Forest, Tower Hamlets, Newham, Redbridge, Havering, Barking and Dagenham), East London NHS Foundation Trust, Bradford Teaching Hospitals NHS Foundation Trust, Public Health England (especially David Wyllie), Discovery Data Service/Endeavour Health Charitable Trust (especially David Stables), Voror Health Technologies Ltd (especially Sophie Don), NHS England (for what was NHS Digital) - for GDPR-compliant data sharing backed by individual written informed consent.

We want to acknowledge the participants and investigators of the FinnGen study. The FinnGen project is funded by two grants from Business Finland (HUS 4685/31/2016 and UH 4386/31/2016) and the following industry partners: AbbVie Inc., AstraZeneca UK Ltd, Biogen MA Inc., Bristol Myers Squibb (and Celgene Corporation & Celgene International II Sàrl), Genentech Inc., Merck Sharp & Dohme LCC, Pfizer Inc., GlaxoSmithKline Intellectual Property Development Ltd., Sanofi US Services Inc., Maze Therapeutics Inc., Janssen Biotech Inc, Novartis Pharma AG, and Boehringer Ingelheim International GmbH. Following biobanks are acknowledged for delivering biobank samples to

FinnGen: Auria Biobank (www.auria.fi/biopankki), THL Biobank (www.thl.fi/biobank), Helsinki Biobank (www.helsinginbiopankki.fi), Biobank Borealis of Northern Finland (https://www.ppshp.fi/Tutkimus-ja-opetus/Biopankki/Pages/Biobank-Borealis-briefly-in-English.aspx), Finnish Clinical Biobank Tampere (www.tays.fi/en-US/Research_and_development/Finnish_Clinical_Biobank_Tampere), Biobank of Eastern Finland (www.ita-suomenbiopankki.fi/en), Central Finland Biobank (www.ksshp.fi/fi-FI/Potilaalle/Biopankki), Finnish Red Cross Blood Service Biobank (www.veripalvelu.fi/verenluovutus/biopankkitoiminta), Terveystalo Biobank (www.terveystalo.com/fi/Yritystietoa/Terveystalo-Biopankki/Biopankki/) and Arctic Biobank (https://www.oulu.fi/en/university/faculties-and-units/faculty-medicine/northern-finland-birth-cohorts-and-arctic-biobank). All Finnish Biobanks are members of BBMRI.fi infrastructure (www.bbmri.fi). Finnish Biobank Cooperative -FINBB (https://finbb.fi/) is the coordinator of BBMRI-ERIC operations in Finland. The Finnish biobank data can be accessed through the Fingenious® services (https://site.fingenious.fi/en/) managed by FINBB. The team of investigators in FinnGen are listed in Table S11.

We want to thank the Genes & Health Research Team (in alphabetical order by surname): Shaheen Akhtar, Mohammad Anwar, Omar Asgar, Samina Ashraf, Saeed Bidi, Gerome Breen, James Broster, Raymond Chung, David Collier, Charles J Curtis, Shabana Chaudhary, Grainne Colligan, Panos Deloukas, Ceri Durham, Faiza Durrani, Fabiola Eto, Sarah Finer, Joseph Gafton, Ana Angel,

Chris Griffiths, Joanne Harvey, Teng Heng, Sam Hodgson, Qin Qin Huang, Matt Hurles, Karen A Hunt, Shapna Hussain, Kamrul Islam, Vivek Iyer, Benjamin M Jacobs, Georgios Kalantzis, Ahsan Khan, Claudia Langenberg, Cath Lavery, Sang Hyuck Lee, Daniel MacArthur, Sidra Malik, Daniel Malawsky, Hilary Martin, Dan Mason, Rohini Mathur, Mohammed Bodrul Mazid, John McDermott, Caroline Morton, Bill Newman, Elizabeth Owor, Asma Qureshi, Shwetha Ramachandrappa, Mehru Raza, Jessry Russell, Nishat Safa, Miriam Samuel, Moneeza Siddiqui, Michael Simpson, John Solly, Marie Spreckley. Daniel Stow, Michael Taylor, Richard C Trembath, Karen Tricker, David A van Heel, Klaudia Walter, Caroline Winckley, Suzanne Wood, John Wright, Ishevanhu Zengeya, Julia Zöllner.

Most of all we thank all of the volunteers participating in Genes & Health.

Supplemental references

1. Moore, C.M., Jacobson, S.A., and Fingerlin, T.E. (2019). Power and Sample Size Calculations for Genetic Association Studies in the Presence of Genetic Model Misspecification. Hum. Hered. *84*, 256–271.

2. Futuyma, D.J. (1986). Evolutionary Biology (Sinauer Associates).

3. Heyne, H.O., Karjalainen, J., Karczewski, K.J., Lemmelä, S.M., Zhou, W., FinnGen, Havulinna, A.S., Kurki, M., Rehm, H.L., Palotie, A., et al. (2023). Mono- and biallelic variant effects on disease at biobank scale. Nature *613*, 519–525.

4. Kurki, M.I., Karjalainen, J., Palta, P., Sipilä, T.P., Kristiansson, K., Donner, K.M., Reeve, M.P., Laivuori, H., Aavikko, M., Kaunisto, M.A., et al. (2023). FinnGen provides genetic insights from a well-phenotyped isolated population. Nature *613*, 508–518.

5. Narasimhan, V.M., Hunt, K.A., Mason, D., Baker, C.L., Karczewski, K.J., Barnes, M.R., Barnett, A.H., Bates, C., Bellary, S., Bockett, N.A., et al. (2016). Health and population effects of rare gene knockouts in adult humans with related parents. Science *352*, 474–477.

6. Li, H. (2013). Aligning sequence reads, clone sequences and assembly contigs with BWA-MEM. arXiv [q-bio.GN].

7. Poplin, R., Ruano-Rubio, V., DePristo, M.A., Fennell, T.J., Carneiro, M.O., Van der Auwera, G.A., Kling, D.E., Gauthier, L.D., Levy-Moonshine, A., Roazen, D., et al. (2017). Scaling accurate genetic variant discovery to tens of thousands of samples. Genomics.

8. McLaren, W., Gil, L., Hunt, S.E., Riat, H.S., Ritchie, G.R.S., Thormann, A., Flicek, P., and Cunningham, F. (2016). The Ensembl Variant Effect Predictor. Genome Biol. *17*, 122.

9. 1000 Genomes Project Consortium, Auton, A., Brooks, L.D., Durbin, R.M., Garrison, E.P., Kang, H.M., Korbel, J.O., Marchini, J.L., McCarthy, S., McVean, G.A., et al. (2015). A global reference for human genetic variation. Nature *526*, 68–74.

10. Bergström, A., McCarthy, S.A., Hui, R., Almarri, M.A., Ayub, Q., Danecek, P., Chen, Y., Felkel, S., Hallast, P., Kamm, J., et al. (2020). Insights into human genetic variation and population history from 929 diverse genomes. Science *367*. https://doi.org/10.1126/science.aay5012.

11. Meyer, H.V. (2020). plinkQC: R package for quality control of plink genetic datasets (Github).

12. McInnes, L., Healy, J., and Melville, J. (2018). UMAP: Uniform Manifold Approximation and Projection for Dimension Reduction. arXiv [stat.ML].

13. Manichaikul, A., Mychaleckyj, J.C., Rich, S.S., Daly, K., Sale, M., and Chen, W.-M. (2010). Robust relationship inference in genome-wide association studies. Bioinformatics *26*, 2867–2873.

14. Wright, C.F., West, B., Tuke, M., Jones, S.E., Patel, K., Laver, T.W., Beaumont,

R.N., Tyrrell, J., Wood, A.R., Frayling, T.M., et al. (2019). Assessing the Pathogenicity, Penetrance, and Expressivity of Putative Disease-Causing Variants in a Population Setting. Am. J. Hum. Genet. *104*, 275–286.

15. Loh, P.-R., Danecek, P., Palamara, P.F., Fuchsberger, C., A Reshef, Y., K Finucane, H., Schoenherr, S., Forer, L., McCarthy, S., Abecasis, G.R., et al. (2016). Reference-based phasing using the Haplotype Reference Consortium panel. Nat. Genet. *48*, 1443–1448.

16. Malawsky, D.S., van Walree, E., Jacobs, B.M., Heng, T.H., Huang, Q.Q., Sabir, A.H., Rahman, S., Sharif, S.M., Khan, A., Mirkov, M.U., et al. (2023). Influence of autozygosity on common disease risk across the phenotypic spectrum. bioRxiv. https://doi.org/10.1101/2023.02.01.23285346.

17. Jacobs, B.M., Stow, D., Hodgson, S., Zöllner, J., Samuel, M., Kanoni, S., Bidi, S., Genes & Health Research Team, Walter, K., Langenberg, C., et al. (2024). Genetic architecture of routinely acquired blood tests in a British South Asian cohort. Nat. Commun. *15*, 8929.

18. Akbaş, A., Kılınç, F., Şener, S., and Hayran, Y. (2023). Vitamin D levels in patients with seborrheic dermatitis. Rev. Assoc. Med. Bras. *69*, e20230022.

19. Dimitrova, J. (2013). Study of the level of 25-hydroxyvitamin D in patients with seborrheic dermatitis. Scr. Sci. Medica *45*, 75.

20. Rahimi, S., Nemati, N., and Shafaei-Tonekaboni, S.S. (2021). Serum levels of 25-hydroxyvitamin D in patients with seborrheic dermatitis: A case-control study. Dermatol. Res. Pract. *2021*, 6623271.

21. Ao, T., Kikuta, J., and Ishii, M. (2021). The effects of vitamin D on immune system and inflammatory diseases. Biomolecules *11*, 1624.

22. Malawsky, D.S., van Walree, E., Jacobs, B.M., Heng, T.H., Huang, Q.Q., Sabir, A.H., Rahman, S., Sharif, S.M., Khan, A., Mirkov, M.U., et al. (2023). Influence of autozygosity on common disease risk across the phenotypic spectrum. Cell *186*, 4514–4527.e14.
